# New Deoxyisoaustamide Derivatives from the Coral-Derived Fungus *Penicillium dimorphosporum* KMM 4689

**DOI:** 10.3390/md19010032

**Published:** 2021-01-12

**Authors:** Olesya I. Zhuravleva, Alexandr S. Antonov, Vo Thi Dieu Trang, Mikhail V. Pivkin, Yuliya V. Khudyakova, Vladimir A. Denisenko, Roman S. Popov, Natalya Y. Kim, Ekaterina A. Yurchenko, Andrey V. Gerasimenko, Anatoly A. Udovenko, Gunhild von Amsberg, Sergey A. Dyshlovoy, Shamil S. Afiyatullov

**Affiliations:** 1 G.B. Elyakov Pacific Institute of Bioorganic Chemistry, Far Eastern Branch of the Russian Academy of Sciences, Prospect 100-letiya Vladivostoka, 159, 690022 Vladivostok, Russia; anotonov_as@piboc.dvo.ru (A.S.A.); pivkin_mv@piboc.dvo.ru (M.V.P.); hudyakova_yv@piboc.dvo.ru (Y.V.K.); vladenis@pidoc.dvo.ru (V.A.D.); popov_rs@piboc.dvo.ru (R.S.P.); kim_ny@piboc.dvo.ru (N.Y.K.); eyurch@piboc.dvo.ru (E.A.Y.); s.dyshlovoy@uke.de (S.A.D.); afiyat@piboc.dvo.ru (S.S.A.); 2 School of Natural Science, Far Eastern Federal University, Sukhanova St., 8, 690000 Vladivostok, Russia; 3 Department of Marine Biotechnology, Nhatrang Institute of Technology Research and Application, Vietnam Academy of Science and Technology, Nha Trang 650000, Vietnam; votrang@nitra.vast.vn; 4 Institute of Chemistry, Far Eastern Branch of the Russian Academy of Sciences, Prospect 100-letiya Vladivostoka, 159, 690022 Vladivostok, Russia; gerasimenko@ich.dvo.ru (A.V.G.); udovenko@ich.dvo.ru (A.A.U.); 5 Laboratory of Experimental Oncology, Department of Oncology, University Medical Center Hamburg-Eppendorf, Hematology and Bone Marrow Transplantation with Section Pneumology, Hubertus Wald-Tumorzentrum, 20246 Hamburg, Germany; g.von-amsberg@uke.de; 6 Martini-Klinik Prostate Cancer Center, University Hospital Hamburg-Eppendorf, 20246 Hamburg, Germany

**Keywords:** *Penicillium dimorphosporum*, secondary metabolites, prenylated indole diketopiperazines, deoxyisoaustamide, neuroprotective activity, paraquat

## Abstract

Seven new deoxyisoaustamide derivatives (**1**–**7**) together with known compounds (**8**–**10**) were isolated from the coral-derived fungus *Penicillium dimorphosporum* KMM 4689. Their structures were established using spectroscopic methods, X-ray diffraction analysis and by comparison with related known compounds. The absolute configurations of some alkaloids were determined based on CD and NOESY data as well as biogenetic considerations. The cytotoxic and neuroprotective activities of some of the isolated compounds were examined and structure-activity relationships were pointed out. New deoxyisoaustamides **4**–**6** at concentration of 1 µM revealed a statistical increase of PQ(paraquat)-treated Neuro-2a cell viability by 30–39%.

## 1. Introduction

Marine-derived fungi are a known source of diketopiperazine-type alkaloids. Indole diketopiperazine alkaloids are characterized by certain products of condensation, including a complete tryptophan and a second amino acid such as L-tryptophan, L-proline, L-phenylalanine, L-histidine, or L-leucine, forming an indole diketopiperazine unit [1]. Among the cyclo-(Try-Pro) prenylated alkaloids, there is a small group containing the rare 6/5/8/6/5 pentacyclic ring system, which includes (+)-deoxyisoaustamide [2], deoxydihydroisoaustamide [3], 16β-hydroxy-17β-methoxy-deoxydihydroisoaustamide [3], carneamides B and C [4], and versicamides A–F [5].

In our search for fungal secondary metabolites possessing novel chemical structures and/or biological activity, we have investigated the strain *Penicillium dimorphosporum* KMM 4689.

Fungi of the genus *Penicillium* have a special position in nature and human life. The marine environment is no exception. These fungi were found in the extremely salty environment of the Dead Sea [6]. The species *Penicillium dimorphosporum* H.J. Swart was described upon the type strain, isolated from a mangrove swamp soil near Westernport Bay near Melbourne, Victoria, Australia, by H.J. Swart, in 1969. Until recently, the species *P. dimorphosporum* assigned to the *P. restrictum* series, the Monoverticillata section on account of the slow growth on most media, the short stipes arising from aerial hyphae, the presence of strongly echinulate globose conidia and monoverticillata conidiophores [7]. Polyphasic taxonomy methods have shown that this species belongs to subgenus *Aspergilloides*, section *Exilicaulis*, series *Erubescentia* [8]. The strain *P. dimorphosporum* KMM 4689 (Collection of Marine Microorganisms, RAS, WDCM #644) was isolated from an unidentified soft coral at South China Sea.

Herein, we report the isolation, structure determination, and biological assay results of the new prenylated indole alkaloids belonging to deoxyisoaustamide family (**1**–**7**), the known (+)-deoxyisoaustamide (**8**) [2,9], deoxydihydroisoaustamide (**9**) [3] and desoxybrevianamide E (**10**) [2] produced by this fungus.

Diketopiperazine alkaloids have shown various biological activities, including cytotoxic [10,11,12,13], antiphytopathogenic [14], anticancer [15] and neuroprotective effects [16]. Earlier it was reported that cryptoechinulin B and other echinuline-related compounds increased the viability of paraquat (PQ)-treated Neuro-2a cells, suppressing the upregulation of intracellular reactive oxygen species [17]. PQ is a bipyridyl quaternary ammonium herbicide and PQ poisoning is one of the leading intoxications worldwide. PQ-induced toxicity has been associated with the induction of oxidative and endoplasmic reticulum stresses, apoptosis, mitochondrial damage, and inflammation, mediated by deregulation of various protein/signaling pathways [18]. These pathological processes result in decreased cell viability and, finally, cell death. In this work, we evaluated the protective effects of the isolated compounds **1**–**10** against the acute toxicity of PQ in murine neuroblastoma Neuro-2a cells which are commonly used for neuroprotective investigations [19].

## 2. Results and Discussion

**Figure 1:**
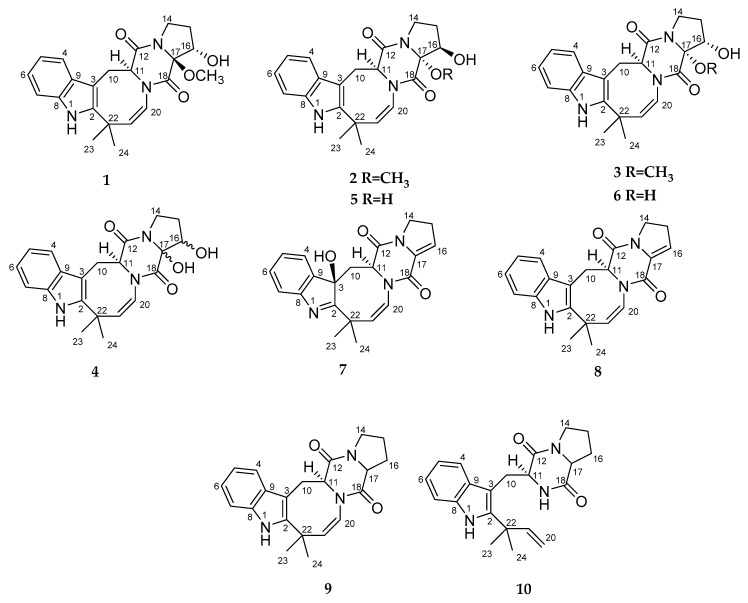
Chemical structures of **1**–**10**.

The fungus was cultured for 21 days on solid rice medium. The EtOAc extract of the mycelium was purified by a combination of a Si gel and an ODS-A column chromatography, and a reversed phase HPLC to yield compounds **1**–**10** (Figure 1).

The molecular formula of **1** was determined as C_22_H_25_N_3_O_4_ from the HRESIMS peak at m/z 394.1771 [M − H]^−^ and was in accordance with the ^13^C NMR data. The ^1^H and ^13^C NMR (Table 1 and Table 2; Figures S21–S27), DEPT and HSQC spectra showed the presence of an NH proton (δ_H_ 10.69), a hydroxy proton (δ_H_ 5.39), three methyl groups (δ_H_ 1.34, 1.46, 1.53, δ_C_ 32.3, 47.6, 28.3), three sp^3^ methylenes (δ_H_ 3.26, 3.60, 1.50, 1.83, 2.69, 3.94, δ_C_ 26.0, 28.8 and 42.6), two sp^3^ methines (δ_H_ 3.90, 4.20, δ_C_ 74.2, 58.6), six olefinic methines (δ_H_ 5.81 (2), 6.90, 6.95, 7.19, 7.32, δ_C_ 140.6, 121.9, 118.6, 120.5, 110.4, 117.4), four sp^2^(δ_C_ 102.8, 128.3, 134.9 and 141.1) and a sp^3^(δ_C_ 37.3) quaternary carbons, an oxygenated quaternary carbon (δ_C_ 93.9) and two amide carbonyls (δ_C_ 162.1 and 166.3).

**Figure 2:**
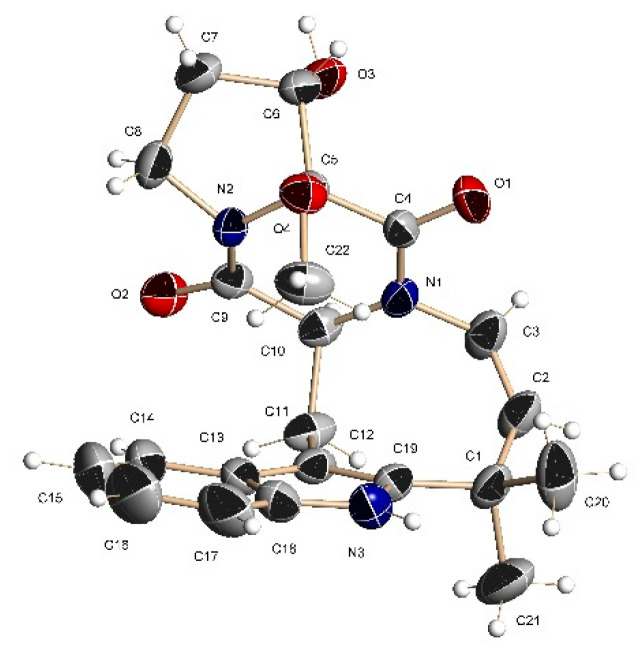
X-ray crystallographic structure of compound **1**.

The ^1^H-^13^C HMBC correlations (Figure S26) from H-1 to C-2 (*δ*_C_ 141.1), C-3 (*δ*_C_ 102.8), C-8 (*δ*_C_ 134.9) and C-9 (*δ*_C_ 128.3), from H-4 to C-3, C-6 (*δ*_C_ 120.5), C-8, С-9, and from H-7 to C-5 (*δ*_C_ 118.6), and C-9 together with the ^1^H–^1^H COSY correlations of H-4/H-5/H-6/H-7 indicated the presence of a disubstituted indole core in **1**. The characteristic NMR data and HMBC correlations from H-11 (δ_H_ 4.20)to C-12 (*δ*_C_ 166.3) and C-18 (*δ*_C_ 162.1) suggested the presence of diketopiperazine ring in **1**. The correlations observed in the COSY and HSQC spectra and long-range correlations from H-14α (δ_H_ 3.94) to C-12 and C-15 (δ_C_ 28.8), from H-16 (δ_H_ 3.90) to C-14 (*δ*_C_ 42.6), C-15 and C-17 (δ_C_ 93.9), and from 17-OCH_3_(δ_H_ 1.46, δ_C_ 47.6) to C-17 indicated that the oxidized proline moiety forms a diketopiperazine ring. The HMBC correlations from H_2_-10 (δ_H_ 3.26, 3.60) to C-2, C-3, C-9, C-11 (δ_C_ 58.6) and C-12, from H-21 (δ_H_ 5.81) to C-2, C-20 (*δ*_C_ 121.9), C-22 (*δ*_C_ 37.3) and C-24 (*δ*_C_ 32.3), and COSY correlations H-11/H_2_-10 and H-20/H21 indicated the formation of a closed cycle between the indole and diketopiperazine parts in **1**. The structure and relative configuration of **1** were further confirmed by X-ray crystallographic analysis carried out for a single crystal obtained by recrystallization from acetonitrile-water (Figure 2 and Tables S1–S3).

The upfield shift of the 17-OCH_3_ signal (*δ*_H_ 1.46) in ^1^H NMR spectrum of **1** is thought to be due to the magnetic anisotropy of the coplanar C-18 carbonyl group.

The configuration of the chiral center C-11 in **1** as *S* was established based on obvious biogenetic relationships with (+)-deoxyisoaustamide (**8**), the optical rotation value of which was in full agreement with the literature data [2,9]. Thus, the absolute configuration of **1** was established as 11*S*,16*S*,17*S*. Compound **1** was named 16α-hydroxy-17β-methoxy-deoxydihydroisoaustamide.

**Figure 3:**
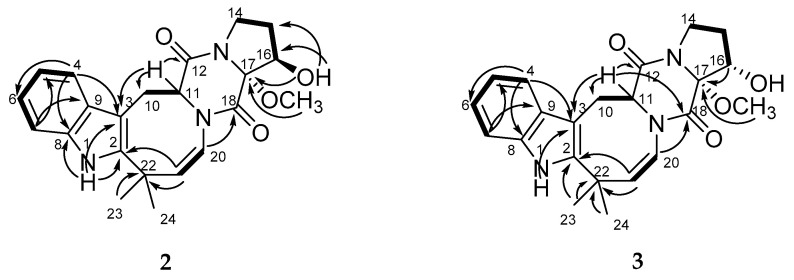
Key COSY and HMBC correlations of **2** and **3**.

The HRESIMS of **2** and **3** showed the quasimolecular ions at m/z 394.1773 [M - H]^−^ and m/z 394.1771 [M - H]^−^, respectively. These data, coupled with ^13^C NMR spectral data (DEPT), established the molecular formula of **2** and **3** as C_22_H_25_N_3_O_4_ for both. A close inspection of the ^1^H and ^13^C NMR data (Table 1 and Table 2 and Figures S28–S41) of **2** and **3** by DEPT and HSQC revealed the presence of disubstituted indole and oxidized proline moieties, diketopiperazine and azocane rings. The main COSY and HMBC correlations showed in Figure 3 indicate that compounds **2** and **3** have the same coplanar structure as **1**.

The absolute configurations of the chiral centers in **2** were defined based on NOESY (Figure S34) correlations 17-OCH_3_(δ_H_ 3.12)/H-11 (δ_H_ 4.56), H-20 (δ_H_ 6.02), H-15α (δ_H_ 1.90), H-16 (δ_H_ 4.01); 16-OH (δ_H_ 4.72)/H-15β (δ_H_ 1.39), H_3_-23(δ_H_ 1.45) and H-11 /H-20, and biogenetic considerations as 11*S*,16*R*,17*R*. Compound **2** was named 16β-hydroxy-17α-methoxy-deoxydihydroisoaustamide.

NOE cross-peaks (Figure S41) 17-OCH_3_(δ_H_ 3.12)/H-11 (δ_H_ 4.30), H-20 (δ_H_ 5.78) and the upfield shift and magnitude of the coupling constant of the H-16 signal (*δ*_H_ 2.01, t, 8.8) suggested the α-orientation of methoxy group at C-17 and α-orientation of hydroxy group at C-16 in **3**. The absolute configurations of the chiral centers in **3** were defined as 11*S,*16*S,*17*R* based on biogenetic consideration. Compound **3** was named 16α-hydroxy-17α-methoxy-deoxydihydroisoaustamide.

The HRESIMS of **4**, **5** and **6** showed the quasimolecular ions at m/z 404.1575 [M + Na]^+^, 404.1583 [M + Na]^+^ and 404.1581 [M + Na]^+^, respectively. These data, coupled with ^13^C NMR spectral data (DEPT), established the molecular formula of all compounds as C_21_H_23_N_3_O_4_.

The general features of ^1^H and ^13^C NMR spectra (Table 1 and Table 2; Figures S42–S49) of **4** showed a close similarity of the proton and carbon chemical shifts to the ones for **1**, with the exception of the C-17 and C-18 carbon signals, and the absence of signals from the methoxy group. The molecular mass difference of 14 mass units between **1** and **4** and ^1^H-^13^C HMBC correlations from 17-OH (δ_H_ 4.21) to C-16 (δ_C_ 74.3) and C-17 (δ_C_ 88.5) and correlation 17-OH/N-13 (δ_N_ 142.3) in ^1^H-^15^N GHMBC spectrum (Figure S48) indicate the presence of a hydroxy group at C-17 in **4**.

NOE cross-peak (Figure S49) 17-OH (δ_H_ 4.21)/H-16 (δ_H_ 3.94) indicated that the hydroxy group at C-17 and the proton at C-16 are on the same side of the proline ring in **4**. The data of NOESY and CD (Figure S4) spectra did not allow to unambiguously establish the configurations of the C-16 and C-17 asymmetric centers in compound **4**. The configuration of the chiral center C-11 in **4** as *S* was established on the basis of biogenetic relationships with (+)-deoxyisoaustamide (**8**). Compound **4** was named 16,17-dihydroxy-deoxydihydroisoaustamide.

The planar structures of the compounds **5** and **6** were found by extensive NMR spectroscopy (^1^H, ^13^C, DEPT, HSQC and HMBC)(Table 1 and Table 2 and Figures S50–S63) to be the same as those of **4**. The general features of ^1^H and ^13^C NMR spectra of **5** and **6** closely resembled those of **2** and **3**, respectively.

**Figure 4:**
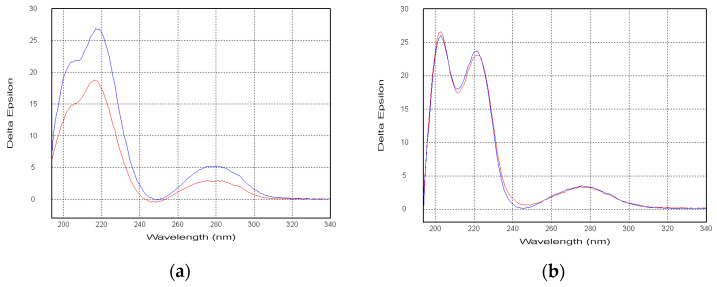
(**a**) CD spectra of **2**(blue) and **5**(red); (**b**) CD spectra of **3**(blue) and **6**(red).

Compounds **5** and **6** exhibited the nearly identical CD spectra to those of **2** and **3**, respectively (Figure 4). Thus, the absolute configurations of the **5** and**6** were established as 11*S*,16*R*,17*R* and 11*S*16*S*17*R*, respectively. Compounds **5** and **6** were named 16β,17α-dihydroxy-deoxydihydroisoaustamide and 16α,17α-dihydroxy-deoxydihydroisoaustamide, respectively.

The HRESIMS of **7** showed the quasimolecular ion at m/z 362.1511 [M - H]^−^. These data, coupled with ^13^C NMR spectral data (DEPT), established the molecular formula of **7** as C_21_H_21_N_3_O_3_. The ^1^H and ^13^C NMR (Table 1 and Table 2 and Figures S64–S70), DEPT and HSQC spectra showed the presence of a hydroxy proton (δ_H_ 5.78), two methyl groups (δ_H_ 1.36, 1.47, δ_C_ 28.3, 26.2), three sp^3^ methylenes (δ_C_ 27.2, 38.7 and 45.2), a methine (δ_H_ 4.08, δ_C_ 58.5), seven olefinic methines (δ_H_ 5.61, 5.66, 5.84, 7.14, 7.20, 7.25, 7.27, δ_C_ 121.3, 117.7, 141.8, 125.6, 124.3, 120.5, 129.2), six sp^2^(δ_C_ 132.6, 140.2, 153.2, 154.5, 160.5 and 187.5) and one sp^3^(δ_C_ 41.8) quaternary carbon and one oxygenated sp^3^ quaternary carbon (δ_C_ 84.7).

The ^1^H-^13^C HMBC (Figure S69) correlations from H-16 (δ_H_ 5.66) to C-14 (*δ*_C_ 45.2), C-15 (*δ*_C_ 27.2), C-17 (*δ*_C_ 132.6) and C-18 (*δ*_C_ 154.5), from H-11 (δ_H_ 4.08) to C-12 (*δ*_C_ 160.5) and C-18 and ^1^H–^1^H COSY correlations H_2_-14/H_2_-15/H-16 revealed the presence of an unsaturated proline moiety and a diketopiperazine ring in **7**. The long-range correlations from 3-OH to C-2 (*δ*_C_ 187.5), C-3 (*δ*_C_ 84.7), C-9 (*δ*_C_ 140.2) and C-10 (*δ*_C_ 38.7), from H-4 (δ_H_ 7.20) to C-3, C-6 (*δ*_C_ 129.2), C-8 (*δ*_C_ 153.2), from H-7 (δ_H_ 7.25) to C-5 (*δ*_C_ 125.6), C-9 and from H-21 to C-2, C-20 (*δ*_C_ 121.3), C-22 (*δ*_C_ 41.8), C-23 (*δ*_C_ 26.2), С-24 (*δ*_C_ 28.3) and C-18 together with ^1^H–^1^H COSY correlations of H-4/H-5/H-6/H-7, H_2_-10/H-11 and H-20/H-21 indicated the location of the hydroxy group at C-3 and established the structures of disubstituted indole core and azocane ring in **7**.

The absolute configurations of the chiral centers in **7** were defined based on NOESY correlations 3-OH (δ_H_ 5.78)/H-21 (δ_H_ 5.84), H_3_-23 (δ_H_ 1.47); H_3_-23 (δ_H_ 1.47)/H-10β (δ_H_ 2.82), H-21; H-11 (δ_H_ 4.08)/H-20 (δ_H_ 5.61)(Figure S70) and biogenetic considerations as 3*R*,11*S*. Compound **7** was named 3β-hydroxy-deoxyisoaustamide.

Of note, we have tried to determine the absolute configuration of the asymmetric centers at C-16 for compounds **1**, **2**, **4** and **5** using the modified Mosher’s method. Esterification of compounds with (*S*)-MTPA chloride at the C-16 hydroxy groups produced (*R*)-MTPA esters. However, the attempts to obtain (*S*)-MTPA esters from (*R*)-MTPA-Cl were unsuccessful.

The structures of known compounds (+)-deoxyisoaustamide (**8**) [2], deoxydihydroisoaustamide (**9**) [3] and desoxybrevianamide E (**10**) [2] were determined based on HRESIMS and NMR data as well as comparison with literature data.

**Figure 5:**
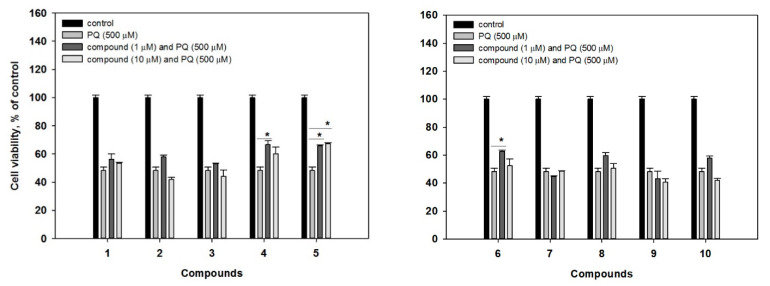
Effects of compounds **1**–**10** on viability of PQ (paraquat)-treated Neuro-2a cells. Each bar represents the mean ± SEM of three independent replicates. (*) indicate *p* < 0.05 versus PQ-treated cells. The difference between control and PQ-treated cells was significant (*p* < 0.05).

We further examined potential protective effects of the isolated compounds **1**–**10** against the acute toxicity of paraquat (PQ) in murine neuroblastoma Neuro-2a cells, which is a well-established model for neuroprotective activity studies [16]. In our experiments, treatment of Neuro-2a cells with 500 μM of PQ induced a decrease of cell viability by 51.8% (Figure 5). Whereas co-treatment with 1 μM of **4** and **6** could increase a viability of PQ-treated cells by 38.6% and 30.3%, respectively. Compound **5** increased a viability of the cells by 36.5% and 39.4% at concentrations at 1 μM and 10 μM, respectively. At the same time, the investigated compounds were non-cytotoxic to Neuro-2a cells, as well as to human prostate epithelial PNT-2 cells (compounds **1**, **2**, **4**, **8**–**10**, Figure S71), used as non-cancer human cell lines to demonstrate the lack of cytotoxic activity to human cells (IC_50_s > 100 μM, data not shown).

This is the first report on neuroprotective activity of deoxyisoaustamides. Earlier, it was reported that (+)-deoxyisoaustamide (**8**) and deoxydihydroisoaustamide (**9**) were studied in glutamate and t-BHP-induced cytotoxicity assays [3]. In addition, inhibitory effects of the metabolites on nitrite production of LPS-stimulated RAW264.7 and BV2 cells were evaluated. These compounds showed no significant effects on cytoprotection or nitrite inhibition [3]. In our experiments, new deoxyisoaustamides **4**–**6** revealed a statistical increase of PQ-treated Neuro-2a cell viability.

The analyses of structure-activity relationships suggest a key role of both hydroxy groups at C-16 and C-17 for the neuroprotective activity of investigated deoxyisoaustamide alkaloids. Indeed, the presence of the mentioned feature in structures **4**–**6** correlates with a higher activity of the compounds.

## 3. Materials and Methods

### 3.1. General Experimental Procedures

Optical rotations were measured on a Perkin-Elmer 343 polarimeter (Perkin Elmer, Waltham, MA, USA). UV spectra were recorded on a Shimadzu UV-1601PC spectrometer (Shimadzu Corporation, Kyoto, Japan) in methanol. CD spectra were measured with a Chirascan-Plus CD spectrometer (Leatherhead, UK) in methanol. NMR spectra were recorded in CD_3_OD and DMSO-d_6_, on a Bruker DPX-500 (Bruker BioSpin GmbH, Rheinstetten, Germany) and a Bruker DRX-700 (Bruker BioSpin GmbH, Rheinstetten, Germany) spectrometer, using TMS as an internal standard. HRESIMS spectra were measured on a Maxis impact mass spectrometer (Bruker Daltonics GmbH, Rheinstetten, Germany). Microscopic examination and photography of fungal cultures were performed with Olympus CX41 microscope fitted with (equipped with) an Olympus SC30 digital camera. Detailed examination of ornamentation of the fungal conidia was performed by scanning electron microscopy (SEM) EVO 40.

Low-pressure liquid column chromatography was performed using Si gel (60/100 μm, Imid Ltd., Russia) and Gel ODS-A (12 nm, S—75 um, YMC Co., Ishikawa, Japan). Plates precoated with Si gel (5–17 μm, 4.5 × 6.0 cm, Imid Ltd., Russia) and Si gel 60 RP-18 F_254_S (20 × 20 cm, Merck KGaA, Germany) were used for thin-layer chromatography. Preparative HPLC was carried out on an Agilent 1100 chromatograph (Agilent Technologies, USA) using a YMC ODS-AM (YMC Co., Ishikawa, Japan)(5 µm, 10 × 250 mm), YMC ODS-A (YMC Co., Ishikawa, Japan)(5 µm, 4.6 × 250 mm) and Supelco Discovery C-18 (5 μm, 250 × 4.6 mm) columns with an Agilent 1100 refractometer (Agilent Technologies, Santa Clara, CA, USA).

### 3.2. Fungal Strain

Soft coral samples were collected using a Van Veen bottom grab at various points in the South China Sea. The samples were collected in individual sterile plastic bags and stored frozen (−18 °C) before use. Isolation of fungal colonies was made by the plating methods using Tubaki agar. Isolation of pure cultures was made by transferring of inoculums to the slant wort agar. Macroscopical characters were studied on the agar media Czapek yeast extract agar (CYA), yeast extract agar (YES) and malt extract agar (MEA). Preparation of these media is detailed by Frisvad and Samson (2004). The strains were inoculated at three points on 9-cm Petri dishes and incubated for 7 d at 25 °C in darkness. In addition, inoculated CYA plates were incubated for 7 d at 37 °C according to the recommendations of Pitt. Color names are from Ridgway. To determine the degree of halotolerance, the strains were grown on MEA supplemented with 5, 10, 15 and 20% NaCl at 25 °C for 7 days.

The cultures used for the molecular studies were grown on malt extract agar under 25 °C for 7 d. DNA extraction was performed by DNA kit (DNA-TechnologyLtd., Moscow, Russia) according to the manufacturer’s instructions. Fragments containing the ITS regions were amplified using primers ITS1 and ITS4 [20].Newly generated sequences were compared to the available sequences of the National Center for Biotechnology Informatic (NCBI) by using BLAST. BLAST search results indicated that the sequence was 99% identical with the sequenceof *Penicillium dimorphosporum* strain CBS 456.70(GenBank accession numberMH859796.1). The sequence was deposited in GenBank nucleotide sequence database under MW325972 code.

### 3.3. Cultivation of Fungus

The fungus was cultured at 22 °C for three weeks in 60 × 500 mL Erlenmeyer flasks, each containing rice (20.0 g), yeast extract (20.0 mg), KH_2_PO_4_(10 mg) and natural sea water from the Marine Experimental Station of PIBOC, Troitsa (Trinity) Bay, Sea of Japan (40 mL).

### 3.4. Extraction and Isolation

At the end of the incubation period, the mycelia and medium were homogenized and extracted with EtOAc (1 L). The obtained extract was concentrated to dryness. The residue (2.8 g) was dissolved in H_2_O−EtOH (4:1)(100 mL) and was extracted with *n*-hexane (0.2 L × 3) and EtOAc (0.2 L × 3). After evaporation of the EtOAc layer, the residual material (1.5 g) was passed over a silica column (3 × 14 cm), which was eluted first with *n*-hexane (200 mL) followed by a step gradient from 5% to 50% EtOAc in *n*-hexane (total volume 20 L). Fractions of 250 mL were collected and combined on the basis of TLC (Si gel, toluene–isopropanol 6:1 and 3:1, *v/v*).

The *n*-hexane-EtOAc fraction (70:30, 250 mg) was separated on a Gel ODS-A column (1.5 × 8 cm), which was eluted by a step gradient from 40% to 80% CH_3_CN in H_2_O (total volume 1 L) to yield subfractions I and II. Subfraction I (60% CH_3_CN, 150 mg) was purified by RP HPLC on a YMC ODS-AM column eluting with CH_3_CN-H_2_O (50:50) to yield subfraction I.I (50 mg) and compounds **8**(17 mg) and **10**(40 mg). Subfraction I.I was purified and separated by RP HPLC on a YMC ODS-A column eluting at first with CH_3_CN-H_2_O (45:55) to yield **1**(19 mg) and **2**(5 mg) and then with CH_3_CN-H_2_O (40:60) to yield **3**(7.5 mg) and **9**(5.5 mg). Subfraction II (40% CH_3_CN, 150 mg) was purified by RP HPLC on a YMC ODS-AM column eluting with CH_3_CN-H_2_O (35:65) and then on a Supelco Discovery C-18 column eluting at first with CH_3_CN-H_2_O (35:65) to yield **4** (8 mg), **5**(5.2 mg), **6**(3.5 mg) and then with CH_3_CN-H_2_O (30:70) to yield **7**(6 mg).

### 3.5. Spectral Data

16α-hydroxy-17β-methoxy-deoxydihydroisoaustamide (**1**): colorless crystal; mp 309–310 °C; [α]_D_^20^ + 171.4 (*c* 0.07 CH_3_OH); CD (*c* 3.0 × 10^−4^, CH_3_OH), λ_max_(∆ε) 206 (+20.77), 213 (+19.90), 276 (+5.32) nm, see Supplementary Figure S1; UV (CH_3_OH)*λ*_max_(log *ε*) 283 (3.83), 223 (4.48) and 198 (4.46) nm, see Supplementary Figure S11; ^1^H and ^13^C NMR data, see Table 1 and Table 2, Supplementary Figures S21–S27; HRESIMS m/z 394.1771 [M − H]^−^ (calcd. for C_22_H_24_N_3_O_4_, 394.1772, Δ +0.3 ppm), 418.1733 [M + Na]^+^ (calcd. for C_22_H_25_N_3_O_4_Na, 418.1737, Δ +1.0 ppm).

16β-hydroxy-17α-methoxy-deoxydihydroisoaustamide (**2**): amorphous solids; [α]_D_^20^ +211.1 (*c* 0.09 CH_3_OH); CD (*c* 3.8 × 10^−4^, CH_3_OH), λ_max_(∆ε) 207 (+21.88), 218 (+26.66), 280 (+5.17) nm, see Supplementary Figure S2; UV (CH_3_OH)*λ*_max_(log *ε*) 283 (3.79), 224 (4.43) and 198 (4.40) nm, see Supplementary Figure S12; ^1^H and ^13^C NMR data, see Table 1 and Table 2, Supplementary Figures S28–S34; HRESIMS m/z 394.1773 [M − H]^−^ (calcd. for C_22_H_24_N_3_O_4_, 394.1772, Δ −0.1 ppm), 418.1733 [M + Na]^+^(calcd. for C_22_H_25_N_3_O_4_Na, 418.1737, Δ +1.0 ppm).

16α-hydroxy-17α-methoxy-deoxydihydroisoaustamide (**3**): amorphous solids; [α]_D_^20^ +198.0 (*c* 0.1 CH_3_OH); CD (*c* 4.2 × 10^−4^, CH_3_OH), λ_max_(∆ε) 203 (+26.54), 222 (+23.02), 275 (+3.29) nm, see Supplementary Figure S3; UV (CH_3_OH)*λ*_max_(log *ε*) 283 (3.65), 224 (4.33) and 198 (4.23) nm, see Supplementary Figure S13; ^1^H and ^13^C NMR data, see Table 1 and Table 2, Supplementary Figures S35–S41; HRESIMS m/z 394.1771 [M − H]− (calcd. for C_22_H_24_N_3_O_4_, 394.1772, Δ +0.4 ppm), 418.1733 [M + Na]^+^(calcd. for C_22_H_25_N_3_O_4_Na, 418.1737, Δ +0.5 ppm).

16,17-dihydroxy-deoxydihydroisoaustamide (**4**): amorphous solids; [α]_D_^20^ +153.7 (*c* 0.08 CH_3_OH); CD (*c* 4.4 × 10^−4^, CH_3_OH), λ_max_(∆ε) 200 (+27.59), 223 (+14.08), 274 (+3.26) nm, see Supplementary Figure S4; UV (CH_3_OH)*λ*_max_(log *ε*) 282 (3.79), 223 (4.41) and 198 (4.35) nm, see Supplementary Figure S14; ^1^H and ^13^C NMR data, see Table 1 and Table 2, Supplementary Figures S42–S49; HRESIMS m/z 380.1617 [M − H]^−^ (calcd. for C_21_H_22_N_3_O_4_, 380.1616, Δ −0.2 ppm), 404.1575 [M + Na]^+^(calcd. for C_21_H_23_N_3_O_4_Na, 404.1581, Δ +1.5 ppm).

16β,17α-dihydroxy-deoxydihydroisoaustamide (**5**)*:* amorphous solids; [α]_D_^20^ +141.7 (*c* 0.12 CH_3_OH); CD (*c* 5.2 × 10^−4^, CH_3_OH), λ_max_(∆ε) 206 (+15.03), 216 (+18.72), 281 (+2.92) nm, see Supplementary Figure S5; UV (CH_3_OH)*λ*_max_(log *ε*) 282 (3.68), 276 (3.65) and 223 (4.23) nm, see Supplementary Figure S15; ^1^H and ^13^C NMR data, see Table 1 and Table 2, Supplementary Figures S50–S56; HRESIMS m/z 380.1617 [M − H]^−^ (calcd. for C_21_H_22_N_3_O_4_, 380.1616, Δ −0.2 ppm), 404.1575 [M + Na]^+^(calcd. for C_21_H_23_N_3_O_4_Na, 404.1581, Δ +1.5 ppm).

16α,17α-dihydroxy-deoxydihydroisoaustamide (**6**): amorphous solids; [α]_D_^20^ +195.4 (*c* 0.13 CH_3_OH); CD (*c* 5.2 × 10^−4^, CH_3_OH), λ_max_(∆ε) 203 (+26.02), 222 (+19.85), 276 (+3.43) nm, see Supplementary Figure S6; UV (CH_3_OH)*λ*_max_(log *ε*) 283 (3.84), 275 (3.80) and 223 (4.41) nm, see Supplementary Figure S16; ^1^H and ^13^C NMR data, see Table 1 and Table 2, Supplementary Figures S57–S63; HRESIMS m/z 382.1764 [M + H]^+^ (calcd. for C_21_H_24_N_3_O_4_, 382.1761, Δ −0.6 ppm), 404.1583 [M + Na]^+^(calcd. for C_21_H_23_N_3_O_4_Na, 404.1581, Δ −0.4 ppm).

3β-hydroxy-deoxyisoaustamide (**7**): amorphous solids; [α]_D_^20^ +182.0 (*c* 0.08 CH_3_OH); CD (*c* 3.7 × 10^−4^, CH_3_OH), λ_max_(∆ε) 219 (–53.92), 237 (+25.27), 272 (+17.00), 295 (+15.50) nm, see Supplementary Figure S7; UV (CH_3_OH)*λ*_max_(log *ε*) 274 (3.85), 223 (4.34) and 195 (4.18) nm, see Supplementary Figure S17; ^1^H and ^13^C NMR data, see Supplementary Figures S64–S70; HRESIMS m/z 404.1581 [M + Na]^+^(calcd. for C_21_H_23_N_3_O_4_Na, 404.1581, Δ +0.0 ppm).

(+)-deoxyisoaustamide (**8**): amorphous solids; [α]_D_^20^ +192.0 (*c* 0.13 CH_3_OH); CD (*c* 4.8 × 10^−4^, CH_3_OH), λ_max_(∆ε) 203 (+4.82), 226 (+11.44), 371 (+7.31) nm, see Supplementary Figure S8; UV (CH_3_OH)*λ*_max_(log *ε*) 273 (3.76), 222 (4.37) and 199 (4.35) nm, see Supplementary Figure S18.

deoxydihydroisoaustamide (**9**): amorphous solids; CD (*c* 4.8 × 10^−4^, CH_3_OH), λ_max_(∆ε) 203 (+9.24), 223 (+20.09), 274 (+5.59) nm, see Supplementary Figure S9; UV (CH_3_OH)*λ*_max_(log *ε*) 283 (3.68), 224 (4.34) and 201 (4.26) nm, see Supplementary Figure S19.

desoxybrevianamide E (**10**): amorphous solids; CD (*c* 4.7 × 10^−4^, CH_3_OH), λ_max_(∆ε) 215 (+7.55), 234 (+2.87), 283 (+2.77) nm, see Supplementary Figure S10; UV (CH_3_OH)*λ*_max_(log *ε*) 283 (3.96), 224 (4.53) and 200 (4.49) nm, see Supplementary Figure S20.

### 3.6. X-ray Crystal Data for **1**

Experimental intensity data for **1** were collected at T = 298(2) K on a BRUKER Kappa APEX2 diffractometer with graphite monochromated Mo Kα radiation (λ = 0.71073 Å). Intensity data were corrected for absorption using the multi-scan method. The structure was solved using direct methods and refined by least-squares calculation in anisotropic approximation for non-hydrogen atoms. Hydrogen atoms were placed in geometrically idealized positions and refined in the riding-model approximation. Data collection, reduction, and refinement of the lattice parameters were performed using the Apex2 software package [21]. All calculations were performed with SHELXL/PC program [22,23]. Main crystallographic data and details of refinement of the crystal structure of 1 are shown in Tables S1–S3. Supplementary crystallographic data (accession numbers CCDC 2048738) can be obtained free of charge from the Cambridge Crystallographic Data Center via http://www.ccdc.cam.ac. uk/data_request/cif (or from the Cambridge Crystallographic Data Centre, 12 Union Road, Cambridge, UK; Fax: +44-1223-336-033 or Email: deposit@ccdc.cam.uk).

### 3.7. Cell Lines and Culture Conditions

The cells of mouse neuroblastoma cell line Neuro-2a and human prostate non-cancer cells PNT-2 were purchased from ATCC (American Type Culture Collection, Manassas, VA, USA). Neuro-2a were cultured in DMEM medium containing 10% fetal bovine serum (Biolot, St. Petersburg, Russia) and 1% penicillin/streptomycin (Biolot, St. Petersburg, Russia) at 37 °C in a humidified atmosphere with 5% (*v/v*) CO_2_. PNT-2 were cultured and handled as previously described [24]. The cells were incubated in cultural flasks until sub-confluent (~80%).

### 3.8. MTT Cell Viability Assay

The Neuro-2a cells (1 × 10^4^ cells/well) or PNT-2 cells (0.6 × 10^4^ cells/well)were seeded in 96-well plate, incubated overnight. Then, the culture medium was exchanged to the fresh corresponding medium containing the different concentrations of the investigated compounds and the cells were further incubated for an additional 24 h. After that, cell viability was determined by MTT (3-(4,5-dimethylthiazol-2-yl)-2,5-diphenyltetrazolium bromide) method according to the manufacturer’s instructions (Sigma-Aldrich, St. Louis, MO, USA). Absorbance of the converted formazan was measured using a Multiskan FC microplate photometer (Thermo Scientific, Waltham, MA, USA) at λ = 570 nm with background subtraction at λ = 630–690 nm. The results were presented as percentages of control data.

### 3.9. Paraquat-Induced Neurotoxicity

The Neuro-2a cells (1 × 10^4^ cells/well of 96-well plate) were pretreated with the studied compounds at concentrations of 1 and 10 µM for 1 h, and then 500 µM of PQ (Sigma-Aldrich, St. Louis, MO, USA) was added (500 µM, final concentration) to the neuroblastoma cells. Cells incubated without PQ and compounds, and with PQ alone, were used as positive and negative controls, respectively. The viability of cells was measured after 24 h using MTT method. The results were presented as percentages of positive control data.

All data were obtained in three independent replicates and calculated values were expressed as mean ± SEM. Student’s t-test was performed using SigmaPlot 14.0 (Systat Software Inc., San Jose, CA, USA) to determine statistical significance.

## 4. Conclusions

Seven new deoxyisoaustamidederivatives (**1**–**7**) together with known compounds (**8**–**10**) were isolated from the fungus *Penicillium dimorphosporum* KMM 4689 associated with unidentified marine soft coral. The absolute configurations of compounds **1**–**3**, **5**–**7** were determined using combined CD and NOESY data as well as biogenetic considerations. All new compounds were found to have an indoloazocine tricyclic subunit which is rare in natural alkaloids. New deoxyisoaustamides **4**–**6** at concentration of 1 µM revealed a statistical increase of PQ-treated Neuro-2a cell viability by 30–39%.

## Figures and Tables

**Table 1:** ^1^H NMR data (*δ* in ppm, *J* in Hz) for compounds (**1**–**7**).

Position	1 ^a^	2 ^b^	3 ^c^	4 ^a^	5 ^b^	6 ^d^	7 ^b^
1	10.69, s	10.55, s	8.13, s	10.70, s	10.60, s		
4	7.32, d (7.7)	7.57, d (8.1)	7.46, d (7.8)	7.27, d (7.7)	7.60, d (7.6)	7.37, d (7.9)	7.20, d (7.1)
5	6.90, t (7.5)	6.88, t (7.6)	7.07, t (7.6)	6.90, t (7.7)	6.88, t (6.6)	6.95, td (7.0, 1.1)	7.14, t (7.1)
6	6.95, t (7.5)	6.94, t (7.6)	7.13, t (7.6)	6.97, t (7.7)	6.94, t (6.6)	7.04, td (7.0, 1.2)	7.27, t (7.1)
7	7.19, d (7.8)	7.17, d (8.1)	7.29, d (8.1)	7.25, d (7.9)	7.17, d (7.6)	7.32, d (8.0)	7.25, d (7.1)
10	α: 3.60, d (14.6) β: 3.26, dd (14.9, 7.1)	a: 3.21, dd (14.6, 5.7) b: 3.65, dd (14.7, 1.8)	a:3.47, dd (14.4, 6.1) b: 3.74, d (14.4)	α: 3.48, dd (14.7, 3.0) β: 3.27, dd (14.7, 6.6)	a: 3.20, m b: 3.61, d (14.7)	α: 3.50, dd (14.5, 6.0) β: 3.60, d (14.5)	α: 2.96, dd (14.5, 1.2) β: 2.82, dd (14.5, 7.1)
11	4.20, d (6.6)	4.56, d (5.4)	4.30, (6.2)	4.20, dd (6.6, 3.0)	4.38, brs	4.30, d (5.8)	4.08, brd (6.7)
14	α: 3.94, m β: 2.69, td (11.0, 3.3)	a: 3.17, m b: 3.27, m	a: 3.09, td (11.0, 3.3) b: 3.19, m	α: 3.68, dt (11.5, 8.4) β: 2.98, ddd (11.7, 10.0, 2.2)	α: 3.29, m β: 3.16, m	α: 3.18, td (10.8, 3.8) β: 3.06, ddd (11.6, 9.2, 2.7)	a: 3.36, td 11.6, (7.7) b: 3.92, td (11.7, 5.5)
15	α: 1.5, m β: 1.83, m	α: 1.90, m β: 1.39, m	α: 1.44, m β: 1.19, m	α: 1.51, ddd (13.3, 8.4, 2.2) β: 1.85, m	α: 2.02, m β: 1.49, m	α: 1.42, m β: 1.20, ddd (12.2, 9.0, 3.5)	a: 2.10, m b: 2.37, m
16	3.90, t (4.9)	4.01, t (4.7)	2.01, t (8.8)	3.94, t (4.4)	4.04, brs	2.47, t (8.5)	5.66, t (2.9)
20	5.81, s	6.02, d (8.6)	5.78, d (8.8)	5.86, d (8.8)	5.94, d (8.5)	5.78, d (8.8)	5.61, d (8.6)
21	5.81, s	5.86, d (8.6)	5.87, d (8.8)	5.73, d (8.8)	5.84, d (8.5)	5.90, d (8.8)	5.84, d (8.6)
23	1.53, s	1.45, s	1.66, s	1.57, s	1.44, s	1.62, s	1.47, s
24	1.34, s	1.42, s	1.38, s	1.38, s	1.44, s	1.37, s	1.36, s
3-OH							5.78, s
16-OH	5.39, d (4.9)	4.72, d (4.3)		5.17, d (4.5)	4.76, d (3.3)		
17-OH				4.21, s	6.63, s		
17-OCH_3_	1.46, s	3.12, s	3.12, s				

^a^ Chemical shifts were measured at 500.13 in DMSO-d_6_. ^b^ Chemical shifts were measured at 700.13 in DMSO-d_6_. ^c^ Chemical shifts were measured at 700.13 in CD_3_OD. ^d^ Chemical shifts were measured at 500.13 in CD_3_OD.

**Table 2:** ^13^С NMR data (*δ* in ppm) for compounds **1**–**7**.

Position	1 ^a^	2 ^b^	3 ^c^	4 ^a^	5 ^b^	6 ^d^	7 ^b^
2	141.1, C	140.7, C	140.8, C	141.3, C	140.5, C	143.4, C	187.5, C
3	102.8, C	102.3, C	103.5, C	102.7, C	102.9, C	104.3, C	84.7, C
4	117.4, CH	119.2, CH	118.4, CH	117.3, CH	119.5, CH	119.7, CH	124.3, CH
5	118.6, CH	117.6, CH	119.8, CH	118.5, CH	117.6, CH	120.4, CH	125.6, CH
6	120.5, CH	120.0, CH	121.9, CH	120.4, CH	120.1, CH	122.6, CH	129.2, CH
7	110.4, CH	110.0, CH	110.6, CH	110.8, CH	109.9, CH	112.4, CH	120.5, CH
8	134.9, C	134.8, C	134.2, C	134.5, C	134.7, C	137.0, C	153.2, C
9	128.3, C	128.6, C	128.0, C	128.2, C	128.8, C	130.0, C	140.2, C
10	26.0, CH_2_	23.8, CH_2_	28.4, CH_2_	26.5, CH_2_	23.2, CH_2_	29.2, CH_2_	38.7, CH_2_
11	58.6, CH	59.2, CH	59.1, CH	59.7, CH	59.4, CH	61.0, CH	58.5, CH
12	166.3, C	165.3, C	165.6, C	165.6, C	164.8, C	168.3, C	160.5, C
14	42.6, CH_2_	42.9, CH_2_	41.1, CH_2_	43.0, CH_2_	42.7, CH_2_	42.4, CH_2_	45.2, CH_2_
15	28.8, CH_2_	28.2, CH_2_	26.1, CH_2_	27.9, CH_2_	28.0, CH_2_	28.1, CH_2_	27.2, CH_2_
16	74.2, CH	73.7, CH	74.2, CH	74.3, CH	74.3, CH	74.8, CH	117.7, CH
17	93.9, C	93.9, C	86.1, C	88.5, C	88.7, C	84.1, C	132.6, C
18	162.1, C	164.1, C	163.4, C	164.5, C	166.8, C	168.2, C	154.5, C
20	121.9, CH	125.1, CH	119.9, CH	123.1, CH	126.4, CH	122.6, CH	121.3, CH
21	140.6, CH	139.6, CH	142.9, CH	139.4, CH	139.2, CH	144.6, CH	141.8, CH
22	37.3, C	36.6, C	37.6, C	37.0, C	36.5, C	39.4, C	41.8, C
23	28.3, CH_3_	30.4, CH_3_	26.0, CH_3_	27.7, CH_3_	30.7, CH_3_	27.8, CH_3_	26.2, CH_3_
24	32.3, CH_3_	32.4, CH_3_	32.4, CH_3_	32.1, CH_3_	32.2, CH_3_	33.3, CH_3_	28.3, CH_3_
17-OCH_3_	47.6, CH_3_	50.7, CH_3_	50.3, CH_3_				

^a^ Chemical shifts were measured at 125.77 in DMSO-d_6_. ^b^ Chemical shifts were measured at 176.04 in DMSO-d_6_. ^c^ Chemical shifts were measured at 176.04 in CD_3_OD. ^d^ Chemical shifts were measured at 125.77in CD_3_OD.

## Data Availability

Data is contained within the article or supplementary material.

## Supplementary Materials

The following are available online at https://www.mdpi.com/1660-3397/19/1/32/s1, 1D and 2D NMR spectra of compounds **1**–**7**; CD and UV spectra of compounds **1**–**10**.
