# Supplementary material for: New Deoxyisoaustamide Derivatives from the Coral-Derived Fungus Penicillium dimorphosporum KMM 4689
Source: Mar Drugs. 2021 Jan 12;19(1):32. doi: 10.3390/md19010032 (PMC7826743; doi:10.3390/md19010032)
Supplement: Supplementary file 1 [file marinedrugs-19-00032-s001.pdf]

SUPPLEMENTARY MATERIAL

New Deoxyisoaustamide Derivatives from the Coral-Derived Fungus *Penicillium dimorphosporum* KMM 4689

Olesya I. Zhuravleva[1,2,*], Alexandr S. Antonov[1], Vo Thi Dieu Trang[3], Mikhail V. Pivkin[1], Yuliya V. Khudyakova[1], Vladimir A. Denisenko[1], Roman S. Popov[1], Natalya Yu. Kim[1], Ekaterina A. Yurchenko[1], Andrey V. Gerasimenko[4], Anatoly A. Udovenko[4], Gunhild von Amsberg[5,6], Sergey A. Dyshlovoy[2,5,6] and Shamil Sh. Afiyatullov[1]

1 G.B. Elyakov Pacific Institute of Bioorganic Chemistry, Far Eastern Branch of the Russian Academy of Sciences, Prospect 100-letiya Vladivostoka, 159, Vladivostok 690022, Russia; anotonov_as@piboc.dvo.ru(A.S.A.); pivkin_mv@piboc.dvo.ru (M.V.P.); hudyakova_yv@piboc.dvo.ru (Y.V.K); vladenis@pidoc.dvo.ru(V.A.D.); popov_rs@piboc.dvo.ru (R.S.P.); kim_ny@piboc.dvo.ru (N.Y.K.); eyurch@piboc.dvo.ru(E.A.Y.); afiyat@piboc.dvo.ru(S.S.A)

2 School of Natural Science, Far Eastern Federal University, Sukhanova St., 8, Vladivostok 690000, Russia; s.dyshlovoy@uke.de

3 Department of Marine Biotechnology, Nhatrang Institute of Technology Research and Application, Vietnam Academy of Science and Technology, Nha Trang 650000, Vietnam; votrang@nitra.vast.vn

4 Institute of Chemistry, Far Eastern Branch of the Russian Academy of Sciences, Prospect 100-letiya Vladivostoka, 159, Vladivostok 690022, Russia; gerasimenko@ich.dvo.ru(A.V.G.); udovenko@ich.dvo.ru(A.A.U.)

5 Laboratory of Experimental Oncology, Department of Oncology, Hematology and Bone Marrow Transplantation with Section Pneumology, Hubertus Wald-Tumorzentrum, University Medical Center Hamburg-Eppendorf, 20246 Hamburg, Germany; g.von-amsberg@uke.de

6 Martini-Klinik Prostate Cancer Center, University Hospital Hamburg-Eppendorf, 20246 Hamburg, Germany

* Correspondence: zhuravleva.oi@dvfu.ru; Tel.: +7-423-231-1168

Abstract: Seven new deoxyisoaustamide derivatives (**1**–**7**) together with known compounds (**8**–**10**) were isolated from the coral-derived fungus *Penicillium dimorphosporum* KMM 4689. Their structures were established using spectroscopic methods, X-ray diffraction analysis and by comparison with related known compounds. The absolute configurations of some alkaloids were determined based on CD and NOESY data as well as biogenetic considerations. The cytotoxic and neuroprotective activities of some of the isolated compounds were examined and structure-activity relationships were discussed.

Keywords: *Penicillium dimorphosporum*, secondary metabolites, prenylated indole diketopiperazines, deoxyisoaustamide, neuroprotective activity; paraquat.

Content

Experimental Section 5
Figure S1. CD spectrum of **1** 5
Figure S2. CD spectrum of **2** 5
Figure S3. CD spectrum of **3** 6
Figure S4. CD spectrum of **4** 6
Figure S5. CD spectrum of **5** 7
Figure S6. CD spectrum of **6** 7
Figure S7. CD spectrum of **7** 8
Figure S8. CD spectrum of **8** 8
Figure S9. CD spectrum of **9** 9
Figure S10. CD spectrum of **10** 9
Figure S11. UV spectrum of **1** 10
Figure S12. UV spectrum of **2** 10
Figure S13. UV spectrum of **3** 11
Figure S14. UV spectrum of **4** 11
Figure S15. UV spectrum of **5** 12
Figure S16. UV spectrum of **6** 12
Figure S17. UV spectrum of **7** 13
Figure S18. UV spectrum of **8** 13
Figure S19. UV spectrum of **9** 14
Figure S20. UV spectrum of **10** 14
Figure S21. ^{1}H NMR spectrum (500 MHz, DMSO-d_6) of **1** 15
Figure S22. ^{13}C NMR spectrum (125 MHz, DMSO-d6) of **1** 16
Figure S23. DEPT-135 NMR spectrum (125 MHz, DMSO-d_6) of **1** 17
Figure S24. COSY-45 spectrum (500 MHz, DMSO-d_6) of **1** 18
Figure S25. HSQC spectrum (500 MHz, DMSO-d_6) of **1** 19
Figure S26. HMBC spectrum (500 MHz, DMSO-d_6) of **1** 20
Figure S27. NOESY spectrum (500 MHz, DMSO-d_6) of **1** 21
Figure S28. ^{1}H NMR spectrum (700 MHz, DMSO-d_6) of **2** 22
Figure S29. ^{13}C NMR spectrum (176 MHz, DMSO-d_6) of **2** 23
Figure S30. DEPT-135 NMR spectrum (176 MHz, DMSO-d_6) of **2** 24
Figure S31. COSY-45 spectrum (700 MHz, DMSO-d_6) of **2** 25
Figure S32. HSQC spectrum (700 MHz, DMSO-d_6) of **2** 26
Figure S33. HMBC spectrum (700 MHz, DMSO-d_6) of **2** 27
Figure S34. NOESY spectrum (700 MHz, DMSO-d_6) of **2** 28
Figure S35. ^{1}H NMR spectrum (700 MHz, CD_3OD) of **3** 29
Figure S36. ^{13}C NMR spectrum (176 MHz, CD_3OD) of **3** 30

Figure S37. DEPT-135 NMR spectrum (176 MHz, CD_3OD) of **3**……………………………………31
Figure S38. COSY-45 spectrum (700 MHz, CD_3OD) of **3**…………………………………………32
Figure S39. HSQC spectrum (700 MHz, CD_3OD) of **3** 33
Figure S40. HMBC spectrum (700 MHz, CD_3OD) of **3** 34
Figure S41. NOESY spectrum (700 MHz, CD_3OD) of **3** 35
Figure S42. ^1H NMR spectrum (500 MHz, DMSO-d_6) of **4**... 36
Figure S43. ^{13}C NMR spectrum (125 MHz, DMSO-d_6) of **4** 37
Figure S44. DEPT-135 NMR spectrum (125 MHz, DMSO-d_6) of **4**………………………………38
Figure S45. COSY-45 spectrum (500 MHz, DMSO-d_6) of **4**………………………………………39
Figure S46. HSQC spectrum (500 MHz, DMSO-d_6) of **4**.. 40
Figure S47. ^1H-^{13}C HMBC spectrum (700 MHz, DMSO-d_6) of **4**.. 41
Figure S48. ^1H-^{15}N GHMBC spectrum (50 MHz, DMSO-d_6) of **4** 42
Figure S49. NOESY spectrum (700 MHz, DMSO-d_6) of **4** 43
Figure S50. ^1H NMR spectrum (700 MHz, DMSO-d_6) of **5**... 44
Figure S51. ^{13}C NMR spectrum (176 MHz, DMSO-d_6) of **5** 45
Figure S52. DEPT-135 NMR spectrum (176 MHz, DMSO-d_6) of **5**………………………………46
Figure S53. COSY-45 spectrum (700 MHz, DMSO-d_6) of **5**……………………………………...47
Figure S54. HSQC spectrum (700 MHz, DMSO-d_6) of **5**.. 48
Figure S55. HMBC spectrum (700 MHz, DMSO-d_6) of **5**.. 49
Figure S56. NOESY spectrum (700 MHz, DMSO-d_6) of **5** 50
Figure S57. ^1H NMR spectrum (500 MHz, CD_3OD) of **6** 51
Figure S58. ^{13}C NMR spectrum (125 MHz, CD_3OD) of **6**... 52
Figure S59. DEPT-135 NMR spectrum (125 MHz, CD_3OD) of **6**…………………………………53
Figure S60. COSY-45 spectrum (500 MHz, CD_3OD) of **6**…………………………………………54
Figure S61. HSQC spectrum (500 MHz, CD_3OD) of **6** 55
Figure S62. HMBC spectrum (500 MHz, CD_3OD) of **6** 56
Figure S63. NOESY spectrum (500 MHz, CD_3OD) of **6**... 57
Figure S64. ^1H NMR spectrum (700 MHz, DMSO-d_6) of **7**... 58
Figure S65. ^{13}C NMR spectrum (176 MHz, DMSO-d_6) of **7**…………….„………………………59
Figure S66. DEPT-135 NMR spectrum (176 MHz, DMSO-d_6) of **7**……………………………...60
Figure S67. COSY-45 spectrum (700 MHz, DMSO-d_6) of **7**………………………………………61
Figure S68. HSQC spectrum (700 MHz, DMSO-d_6) of **7**.. 62
Figure S69. HMBC spectrum (700 MHz, DMSO-d_6) of **7**.. 63
Figure S70. NOESY spectrum (700 MHz, DMSO-d_6) of 7 64
Figure S71. Viability of human prostate PNT2 cells treated with the investigated compounds for 48 h. ……………………………………………………………………………………………65
Table S1. Selected crystal data and refinement parameters for structure **1**………..……………66
Table S2. Selected bond lengths (d, Å) in the structures **1** 67

Table S3. Hydrogen bonds for structure **1** 68

Experimental Section

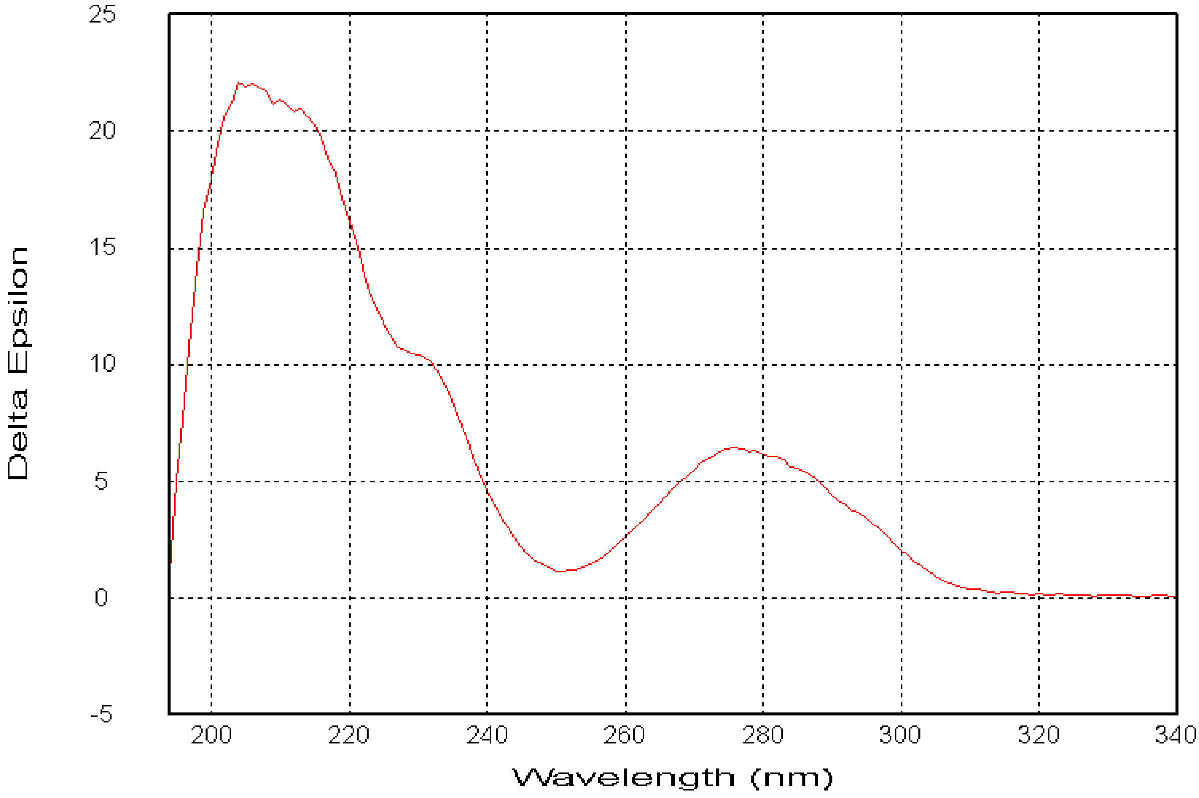

Figure S1. CD spectrum of **1**

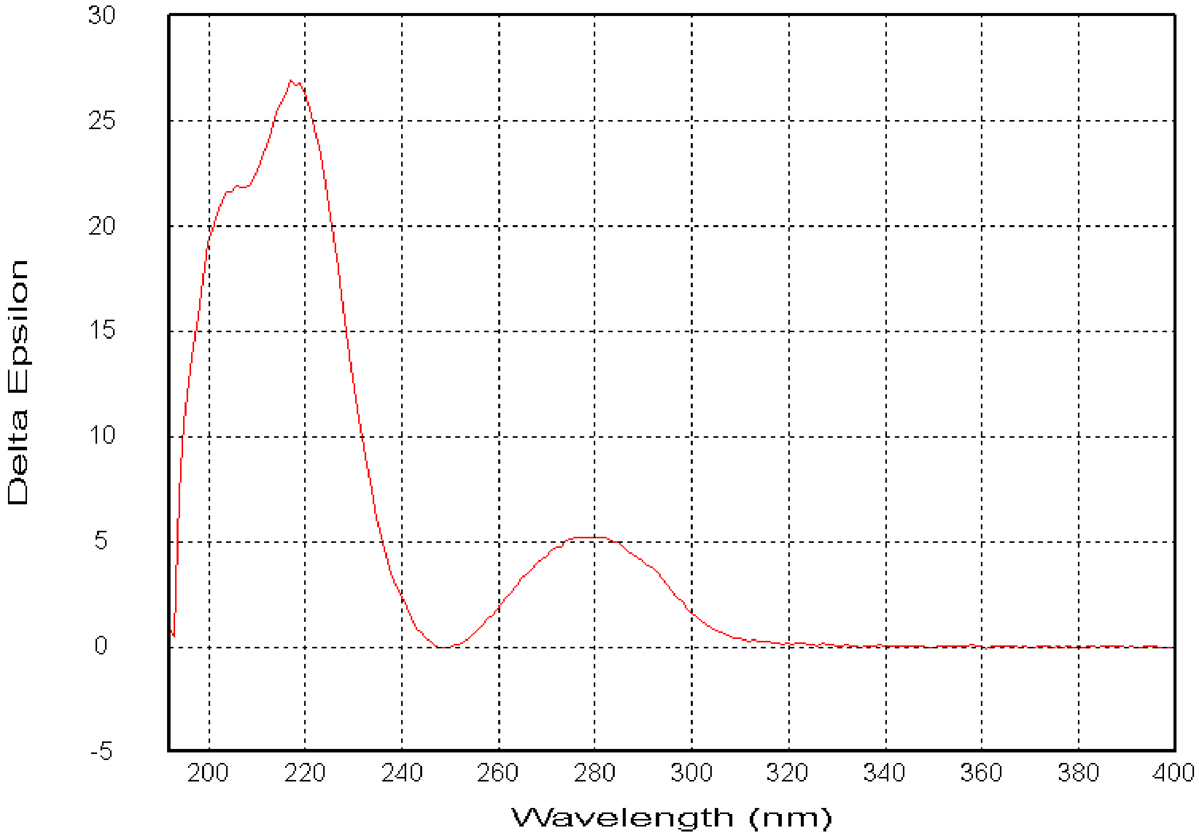

Figure S2. CD spectrum of **2**

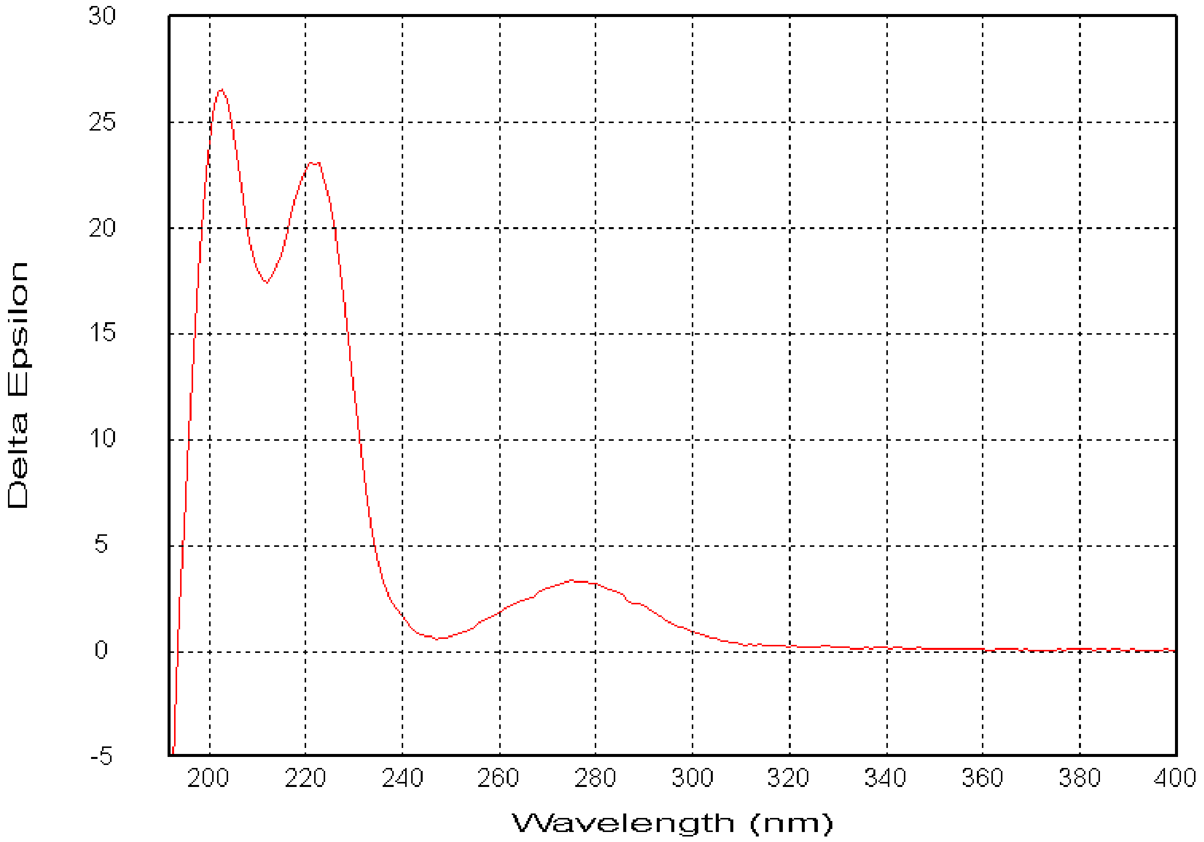

Figure S3.

CD spectrum of **3**

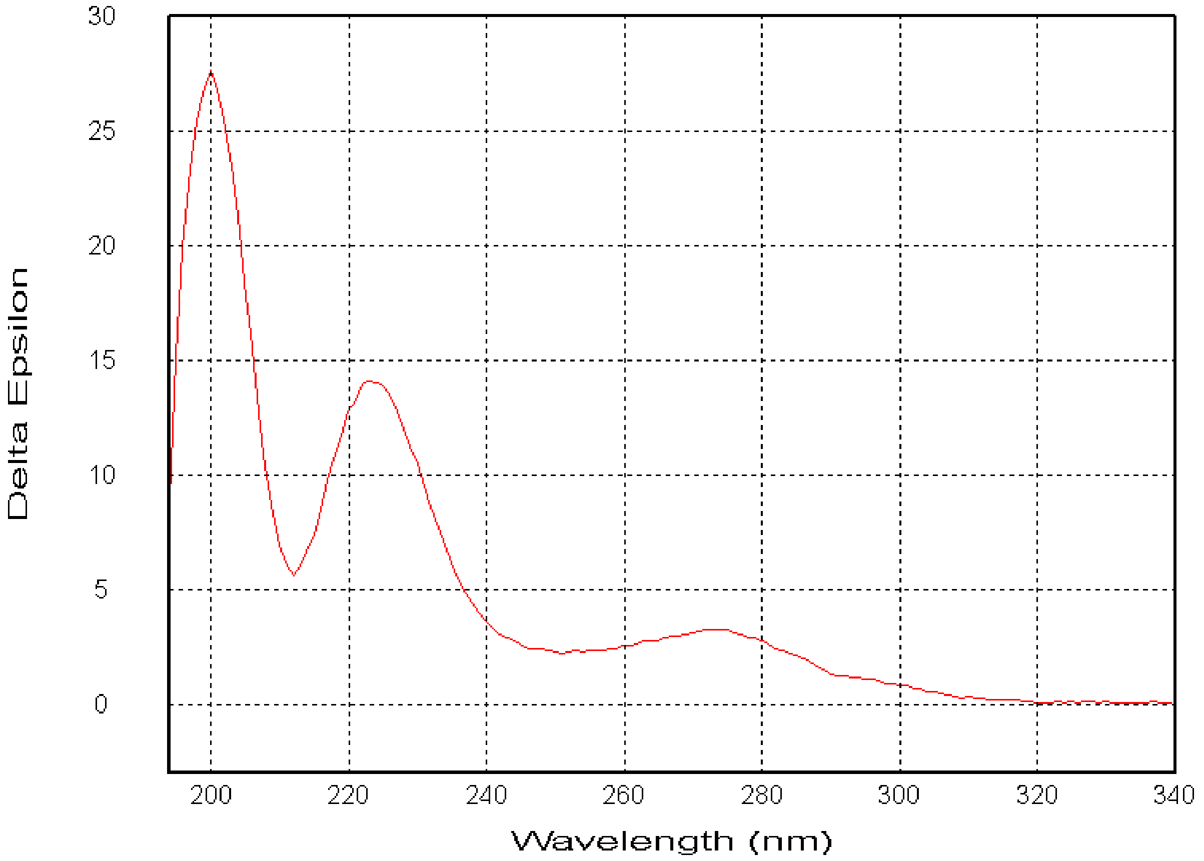

Figure S4. CD spectrum of **4**

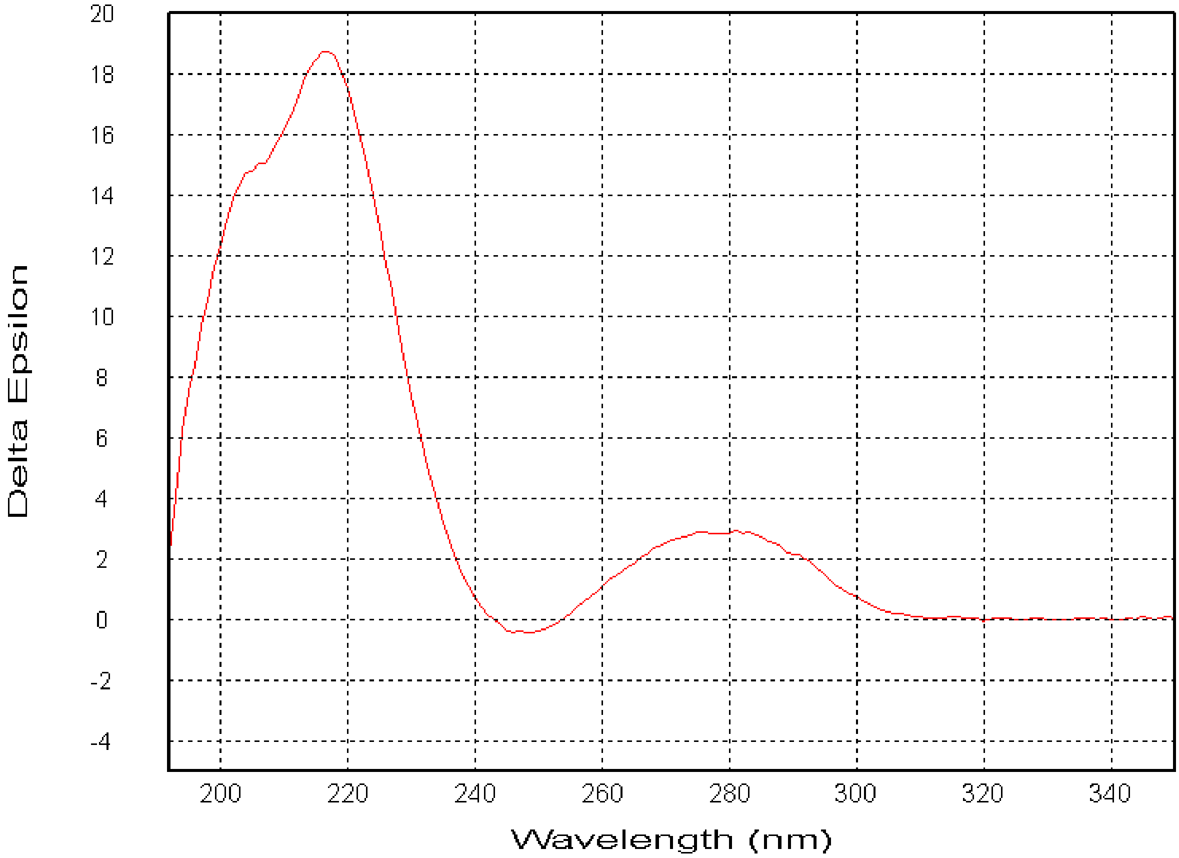

Figure S5. CD spectrum of **5**

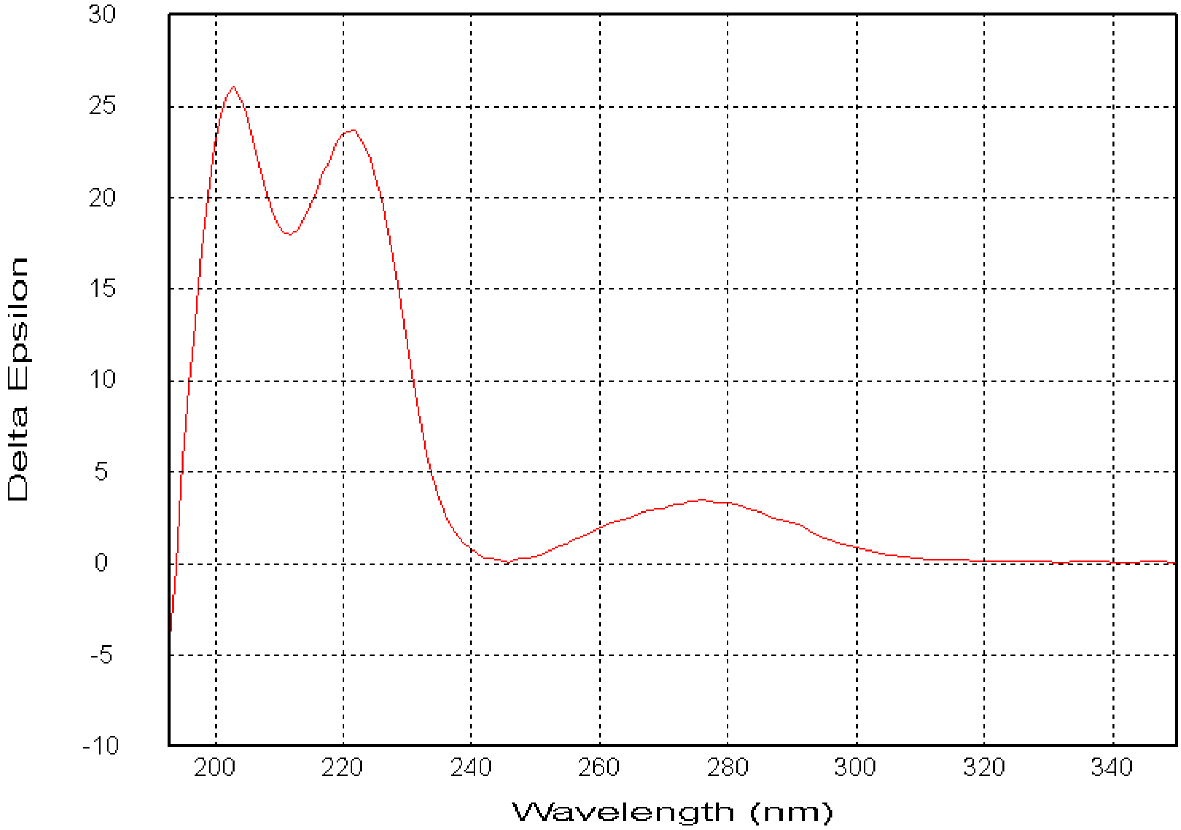

Figure S6. CD spectrum of **6**

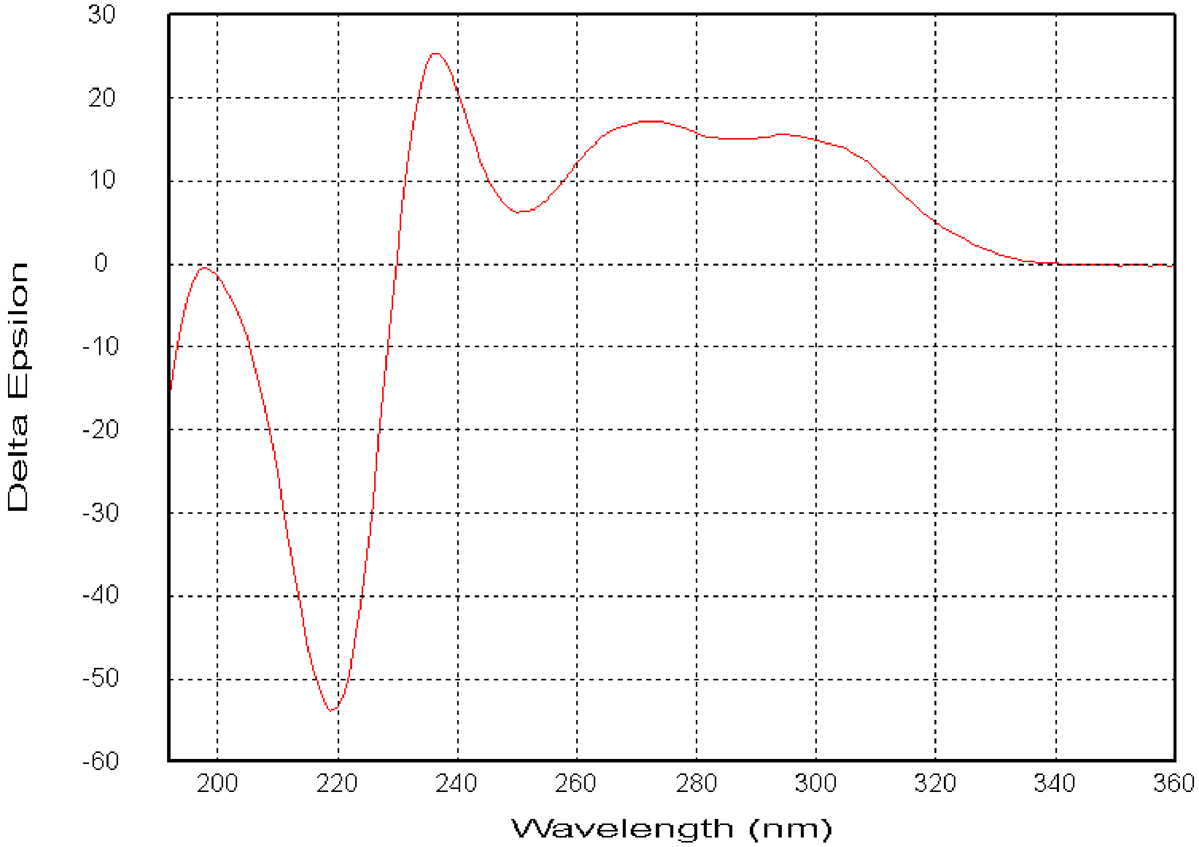

Figure S7.

CD spectrum of **7**

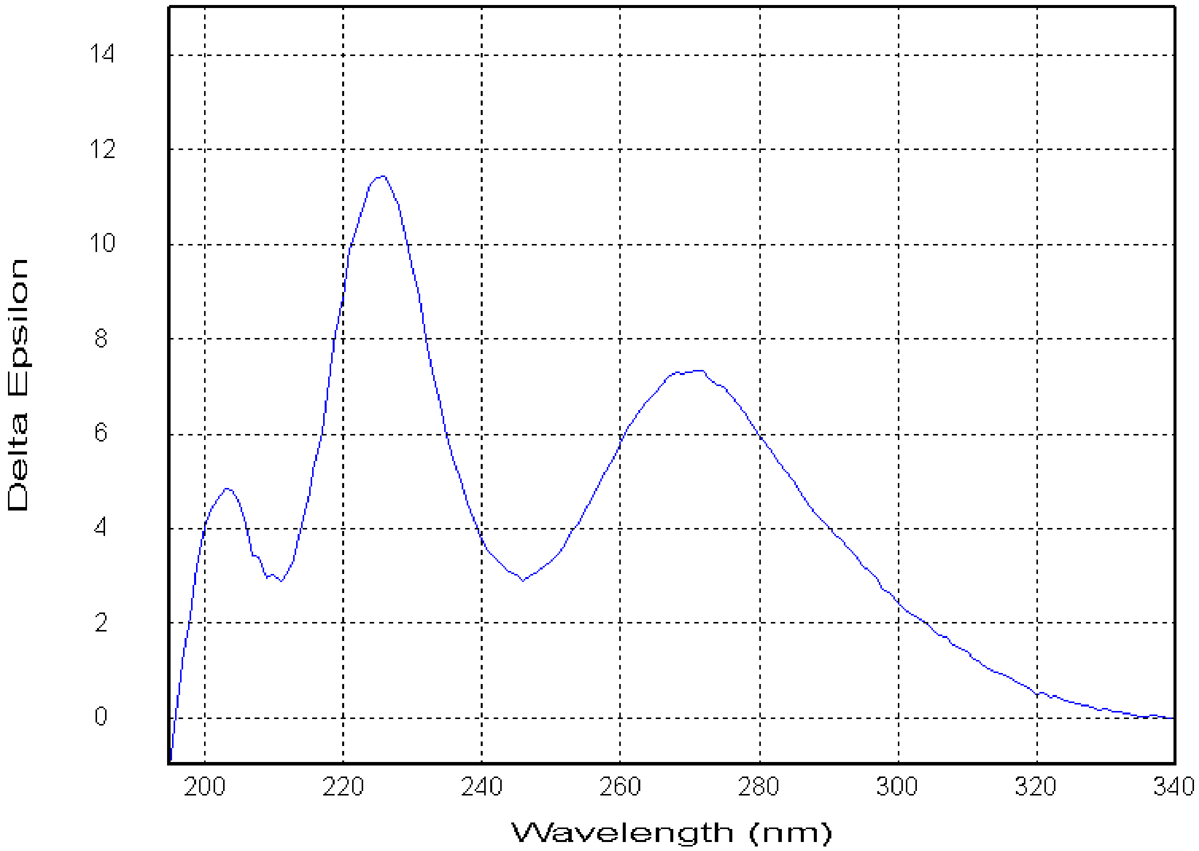

Figure S8.

CD spectrum of **8**

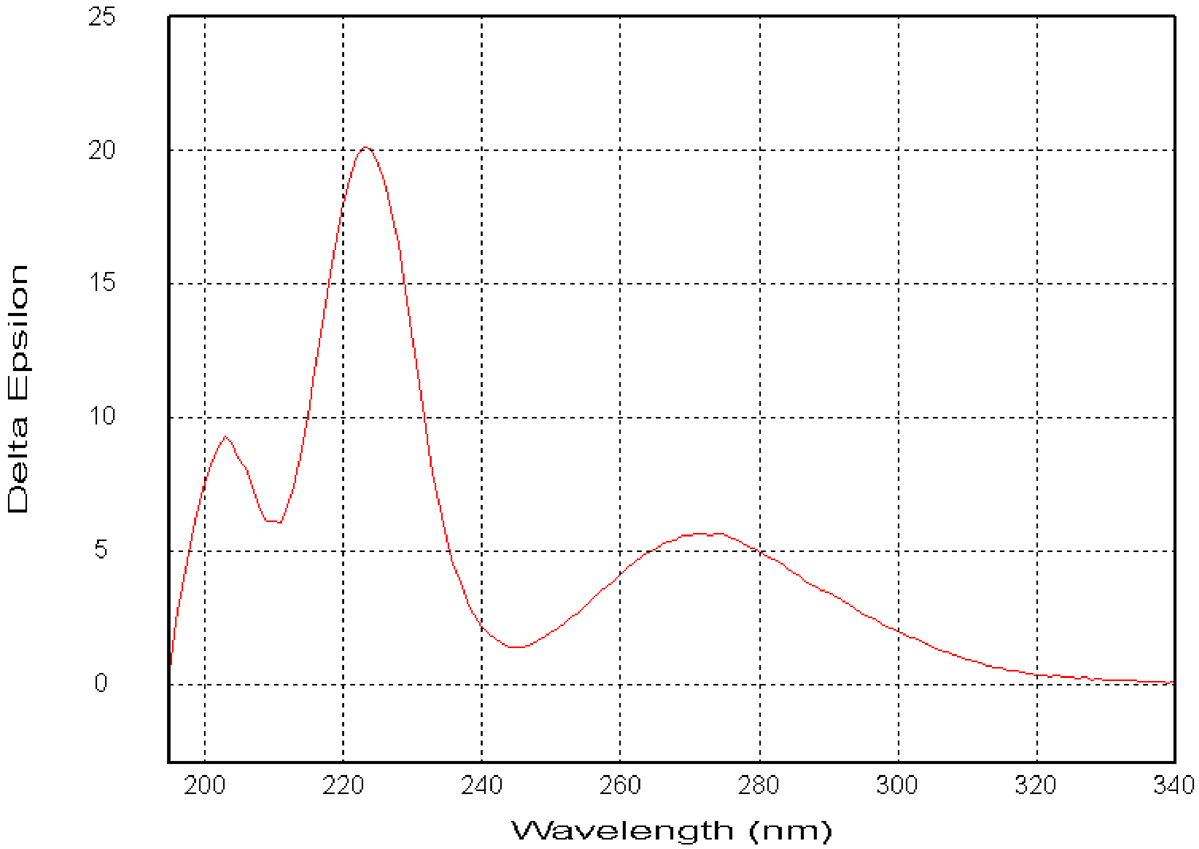

Figure S9. CD spectrum of **9**

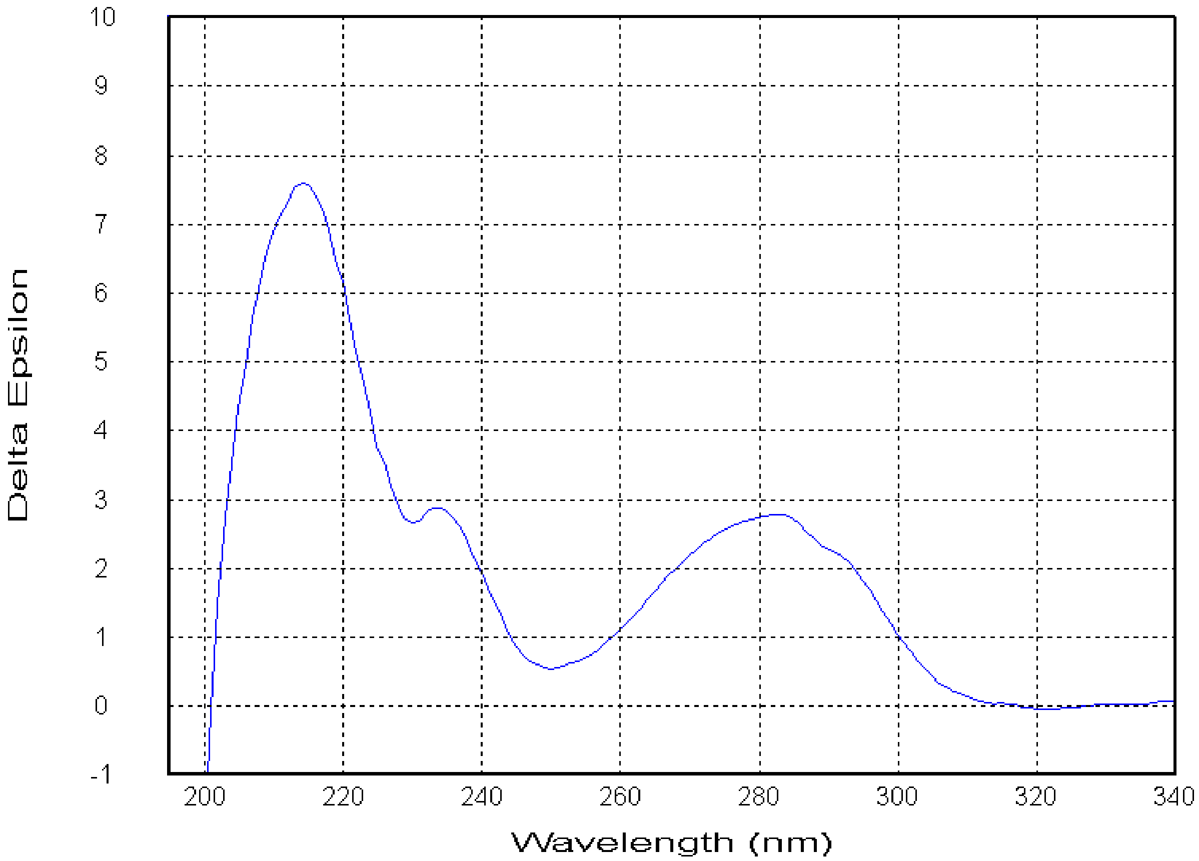

Figure S10. CD spectrum of **10**

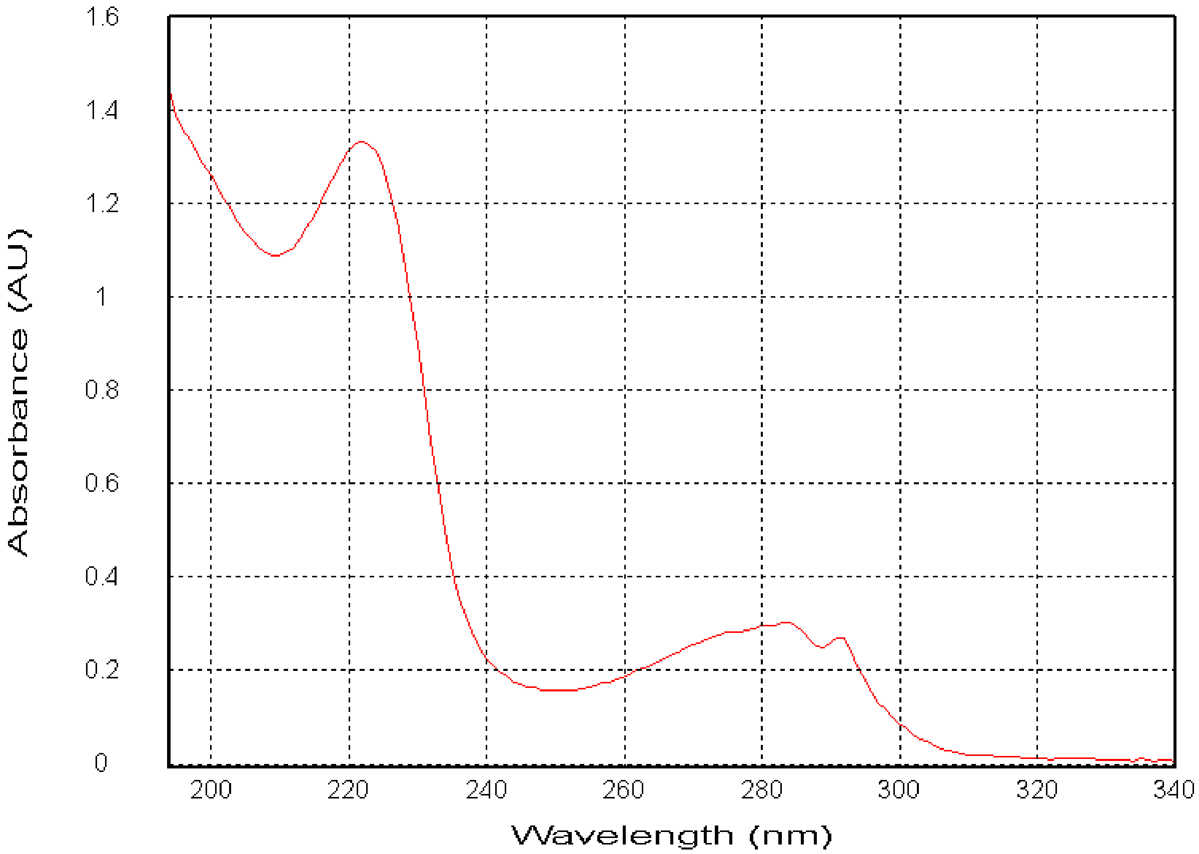

Figure S11. UV spectrum of **1**

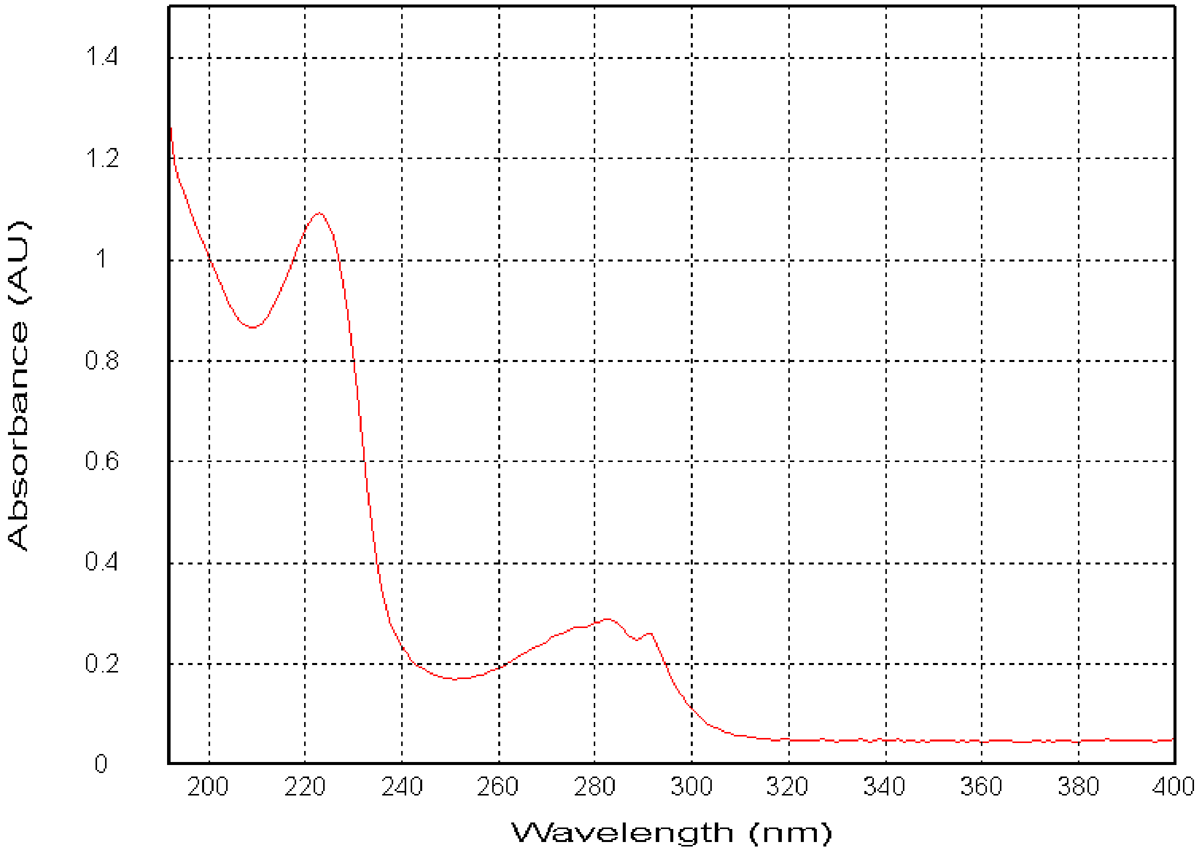

Figure S12. UV spectrum of **2**

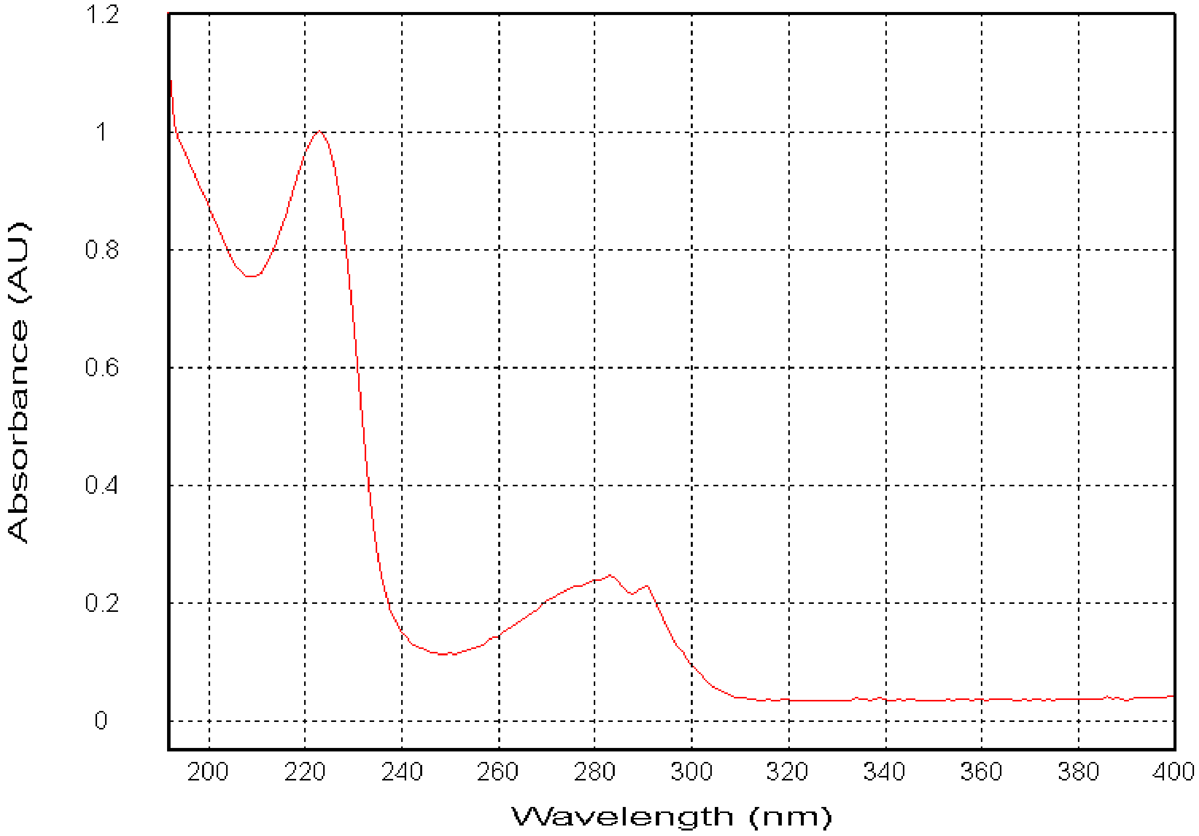

Figure S13. UV spectrum of **3**

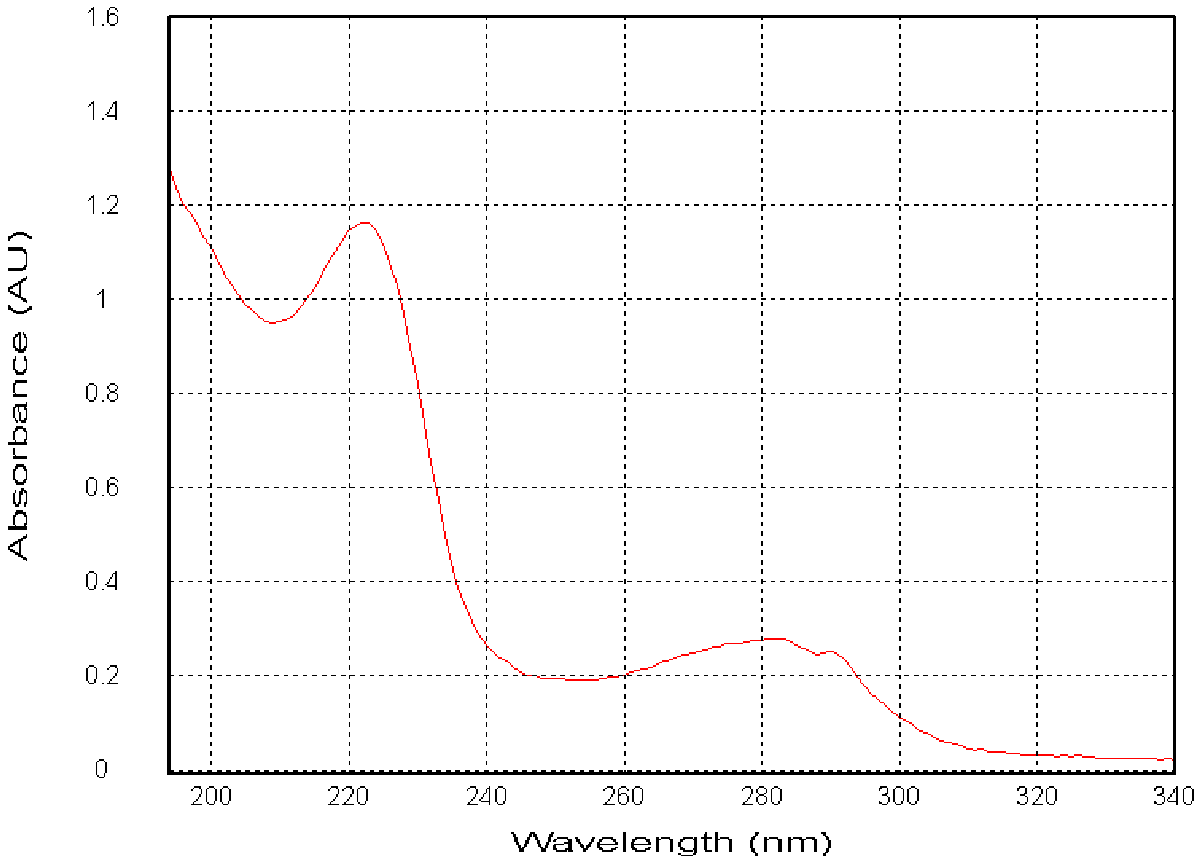

Figure S14. UV spectrum of **4**

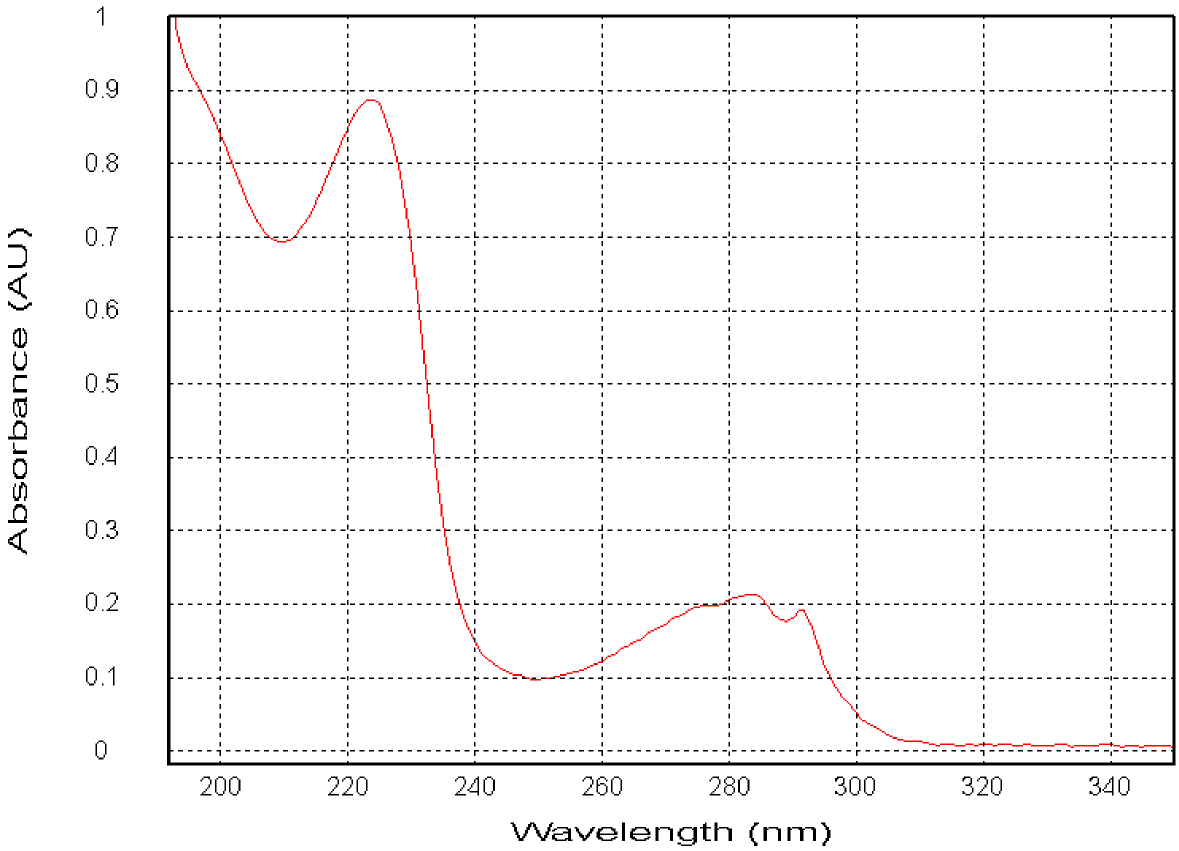

Figure S15. UV spectrum of **5**

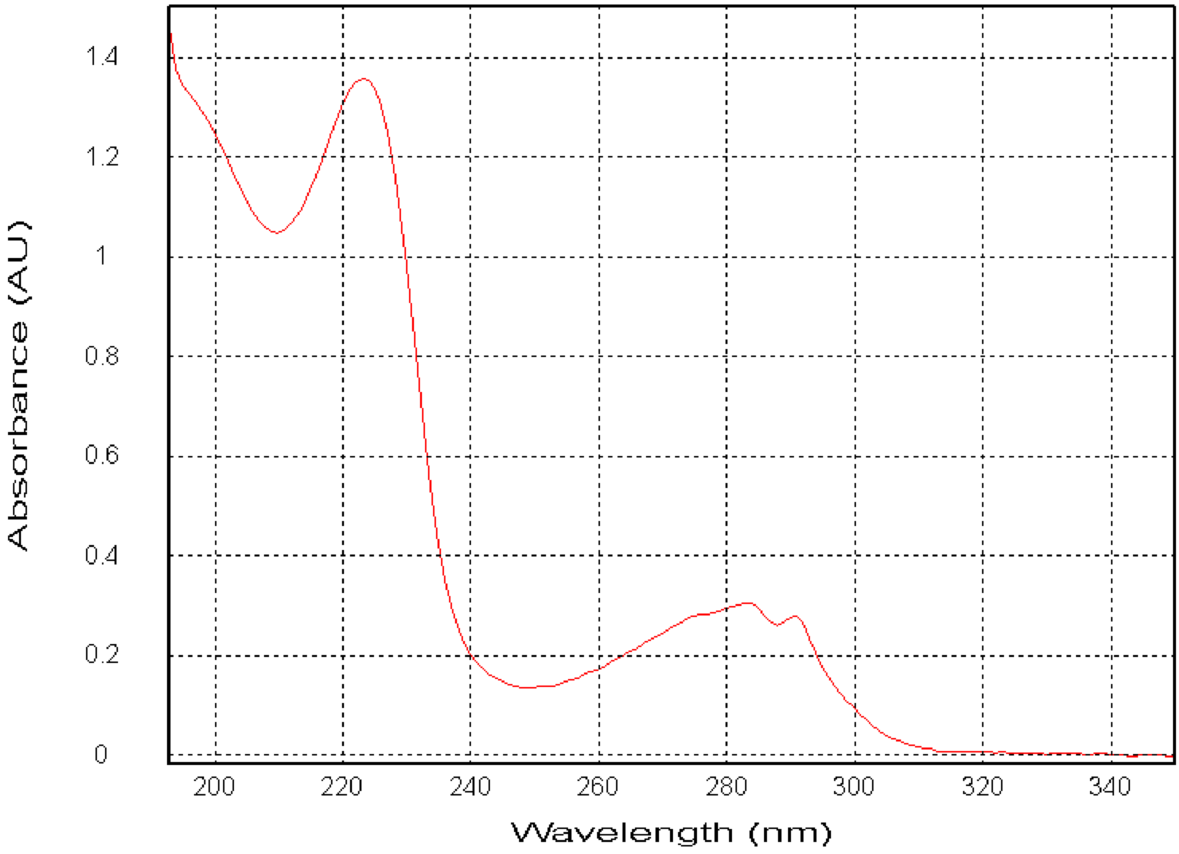

Figure S16. UV spectrum of **6**

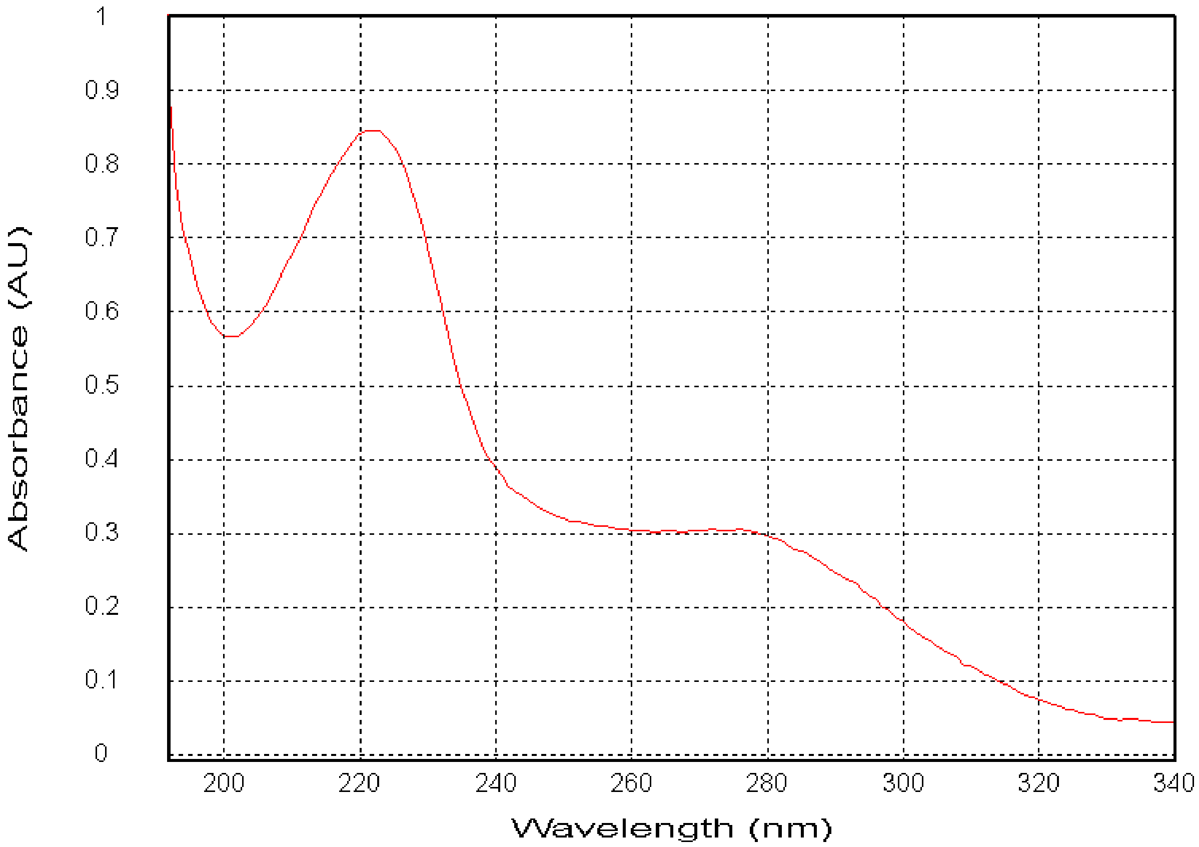

Figure S17. UV spectrum of **7**

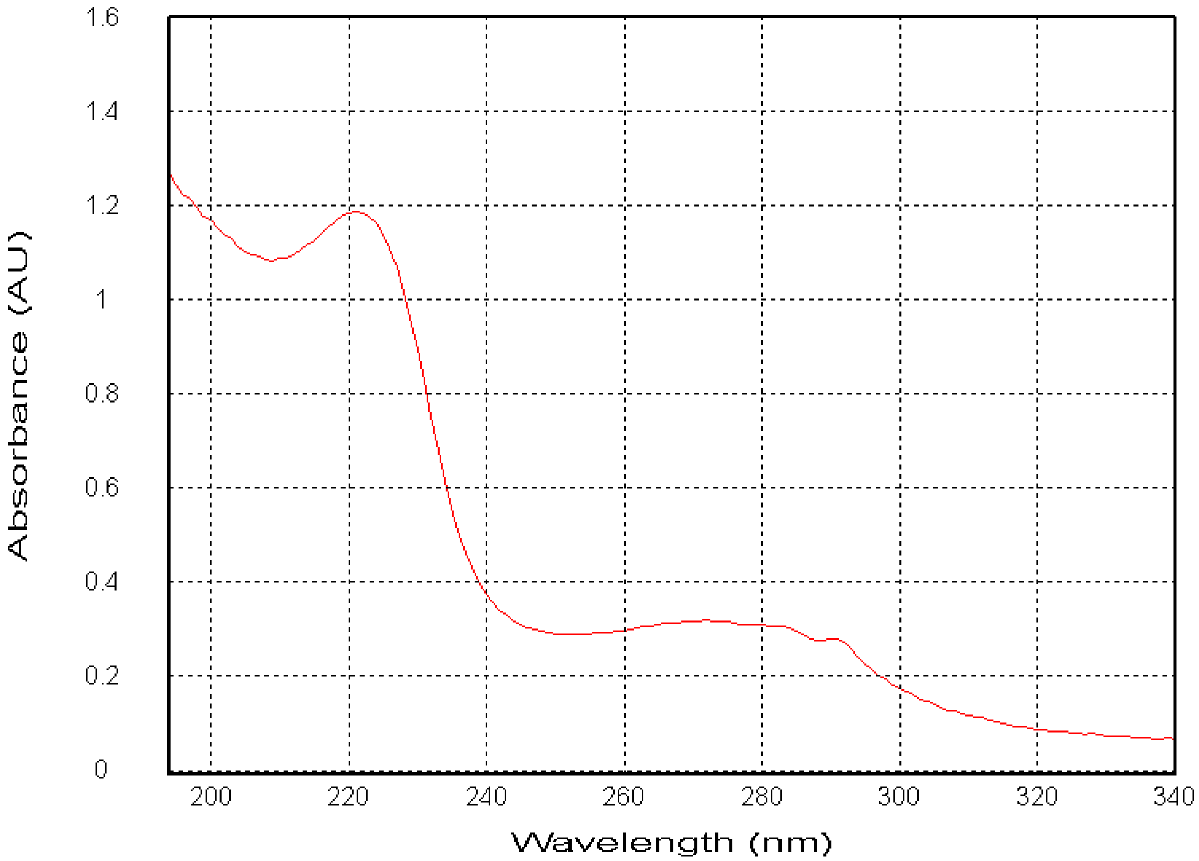

Figure S18. UV spectrum of **8**

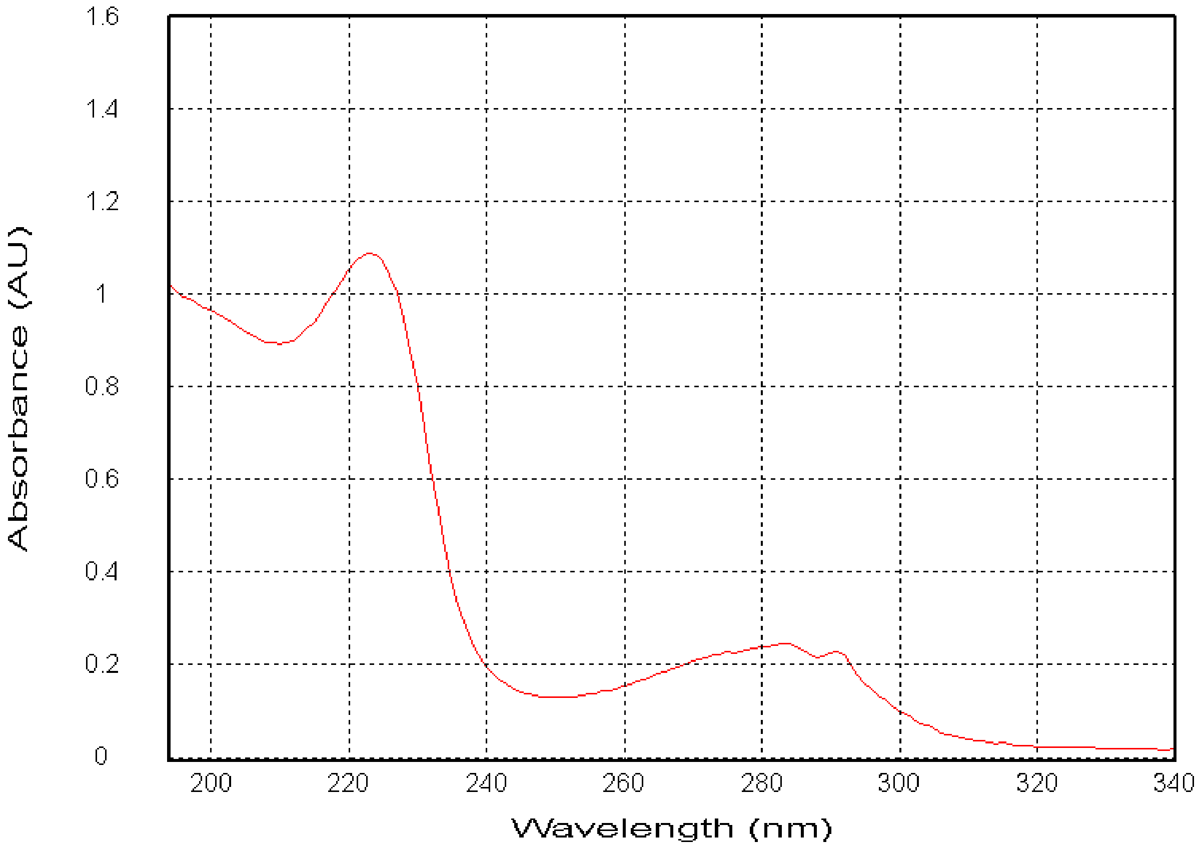

Figure S19. UV spectrum of **9**

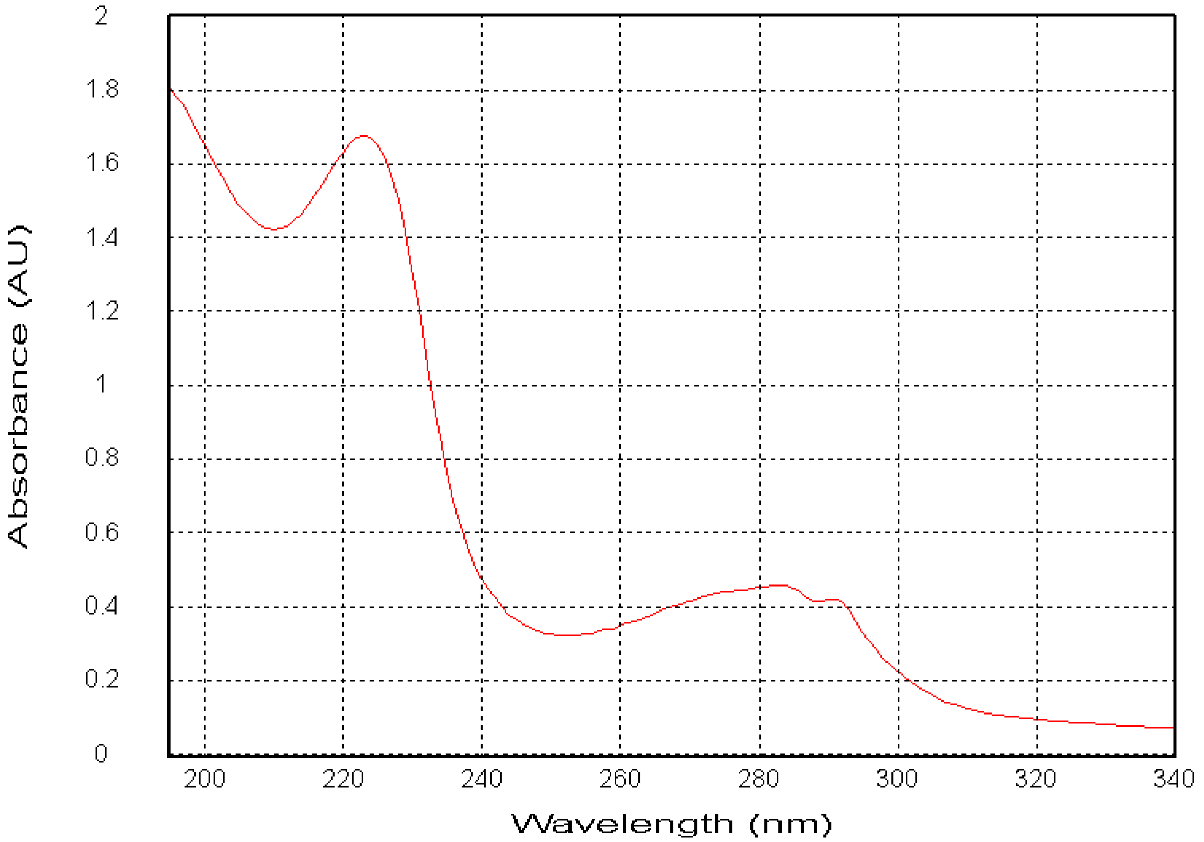

Figure S20. UV spectrum of **10**

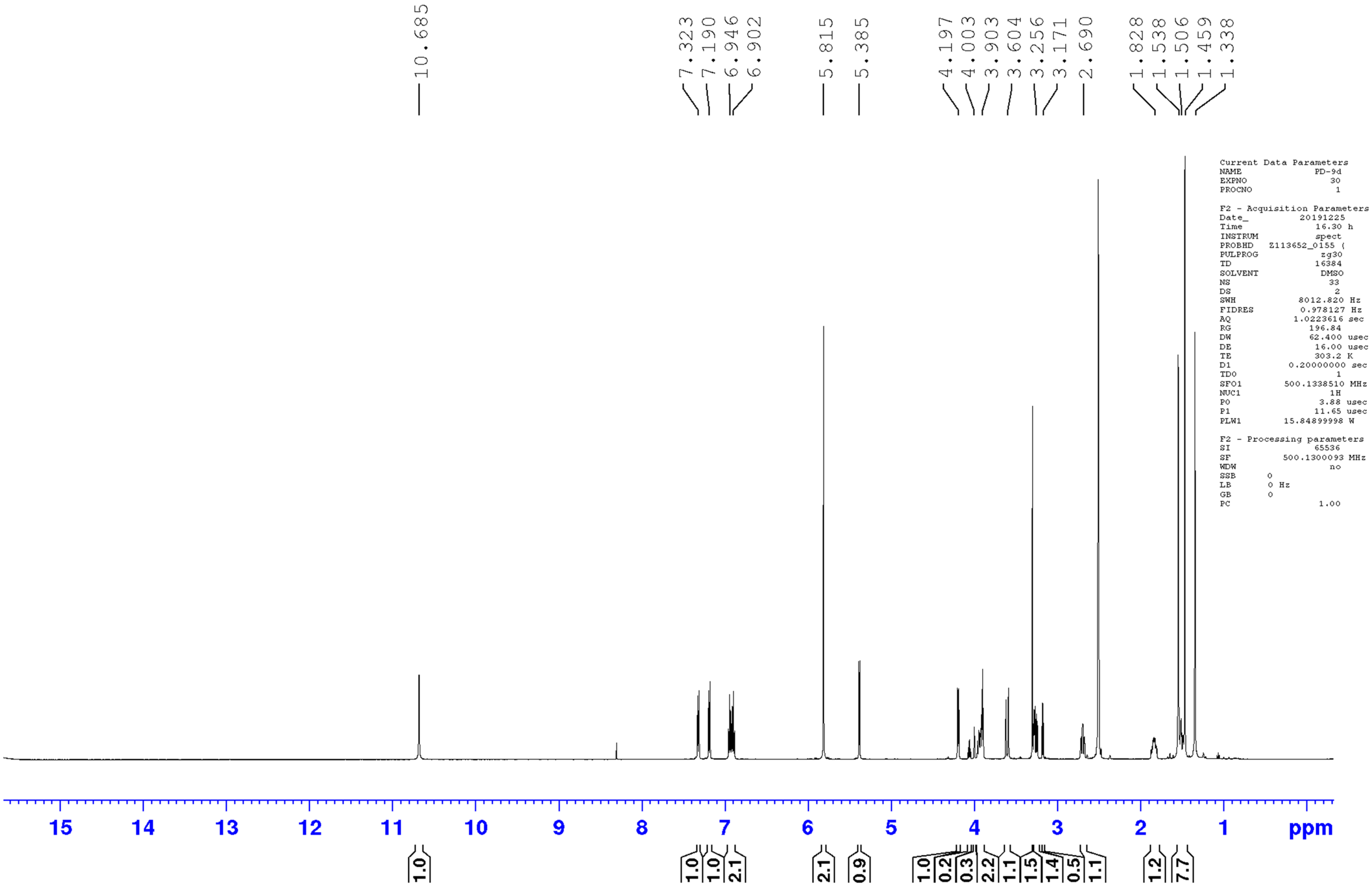

Figure S21. ^{1}H NMR spectrum (500 MHz, DMSO-d6) of **1**

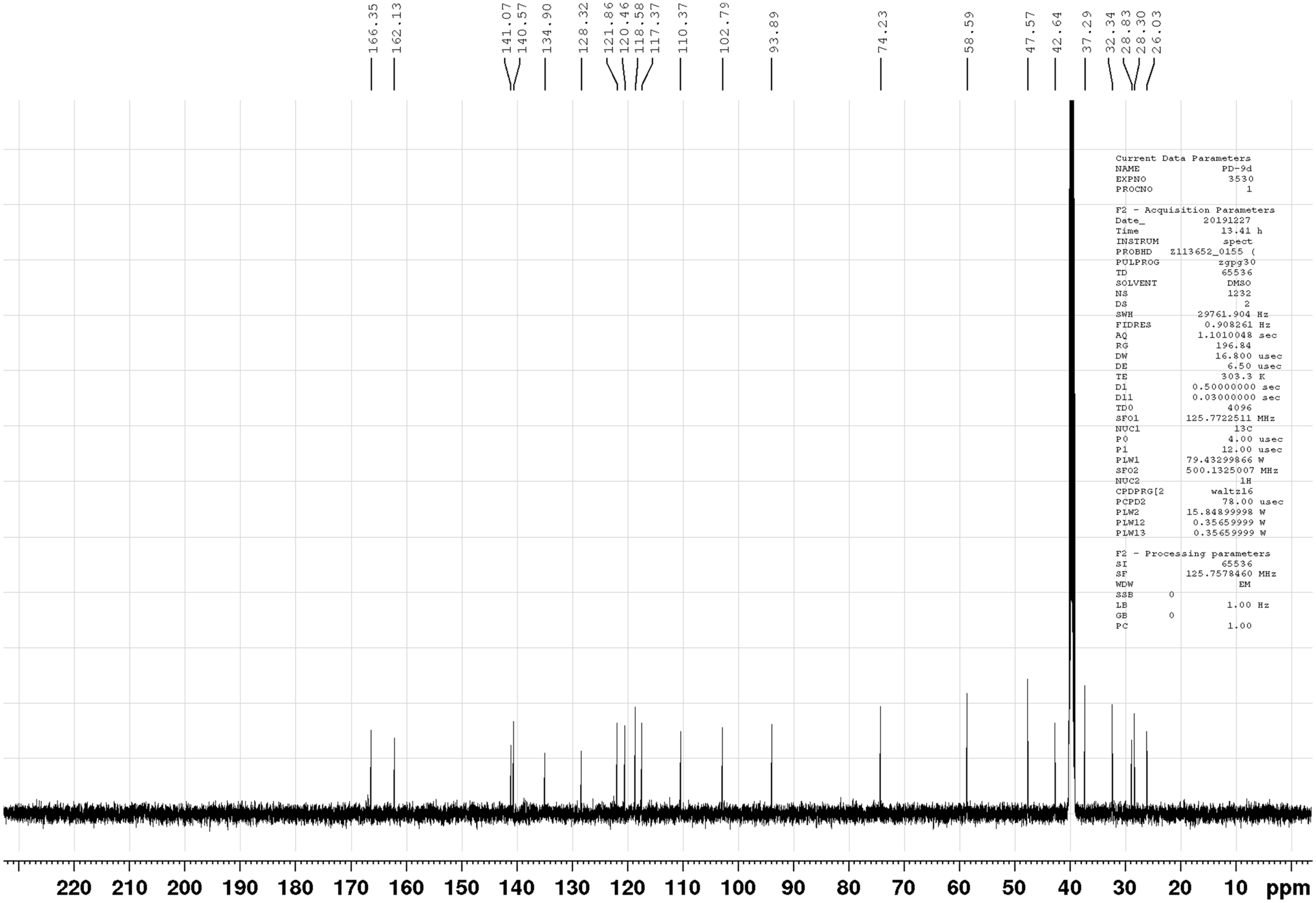

Figure S22. ^{13}C NMR spectrum (125 MHz, DMSO-d6) of **1**

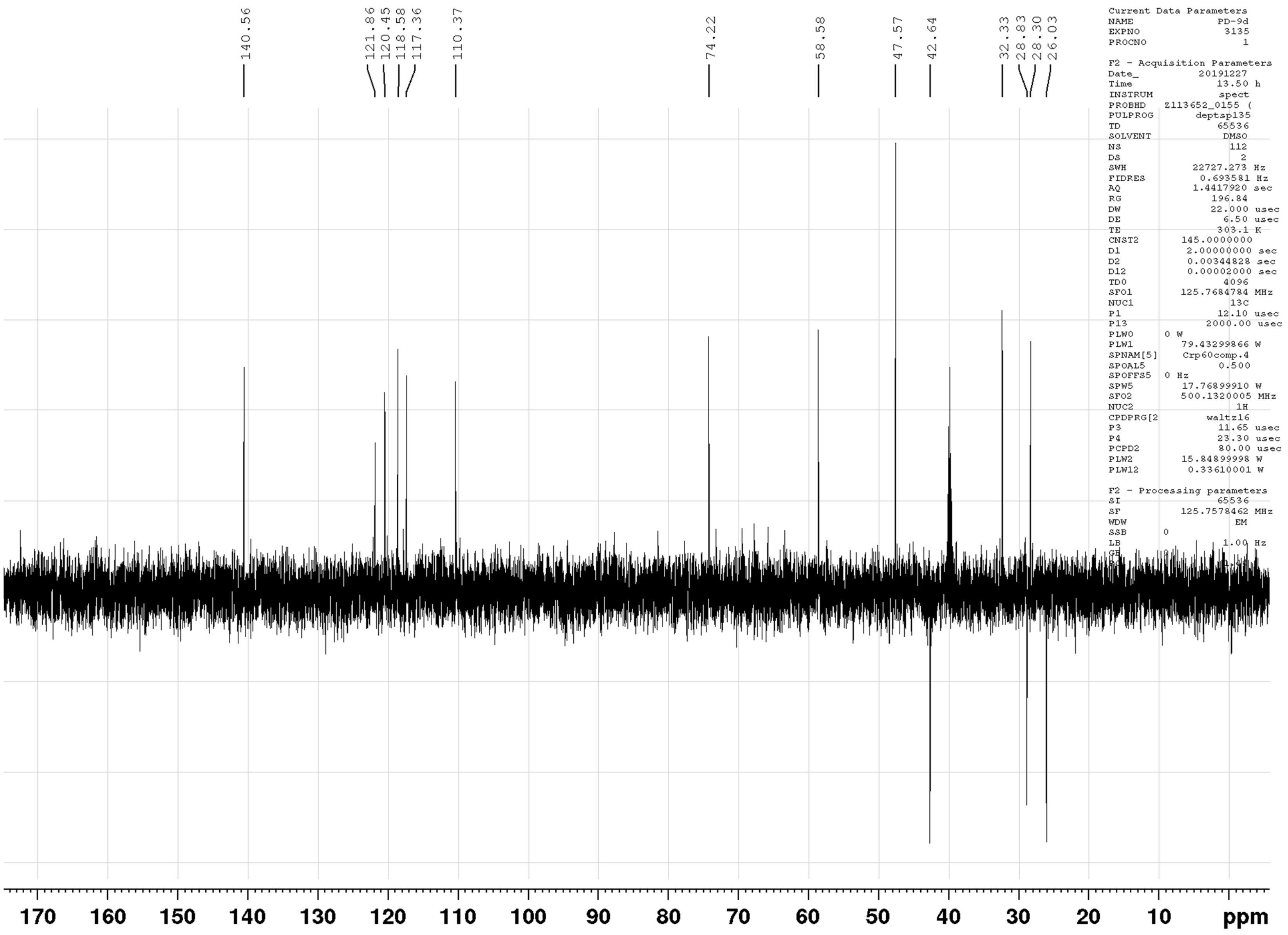

Figure S23. DEPT-135 NMR spectrum (125 MHz, DMSO-d6) of **1**

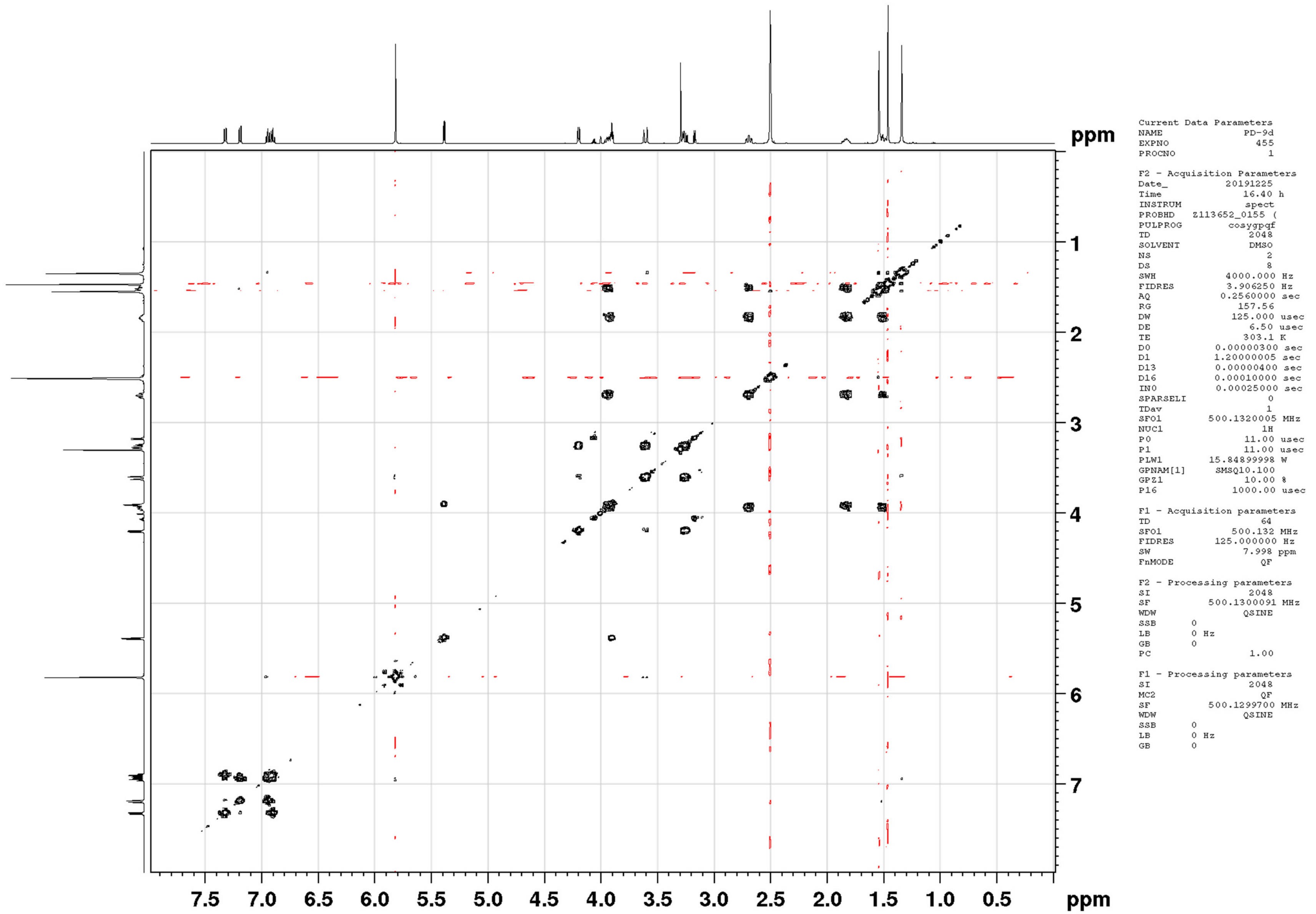

Figure S24. COSY-45 spectrum (500 MHz, DMSO-d6) of **1**

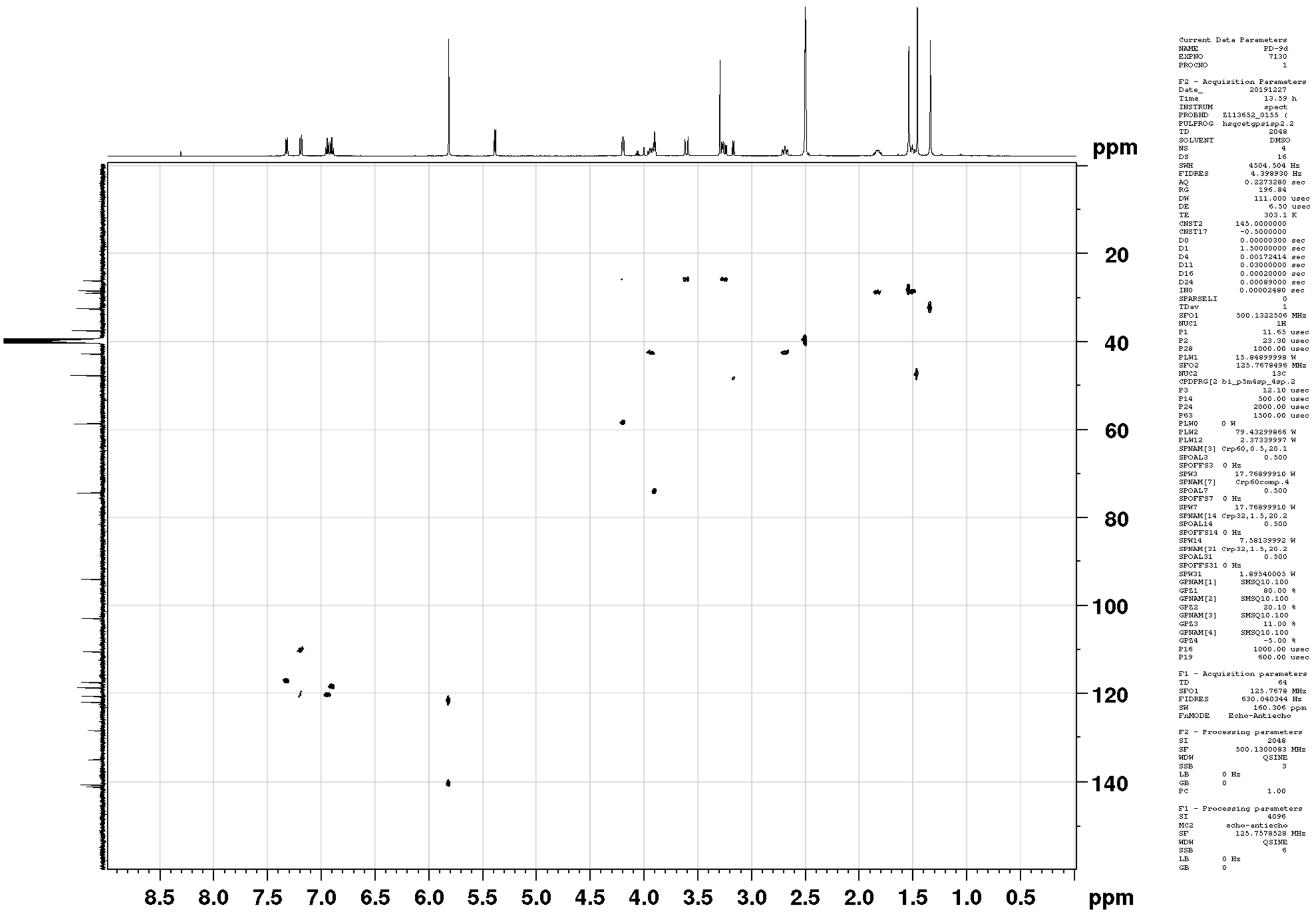

Figure S25. HSQC spectrum (500 MHz, DMSO-d6) of **1**

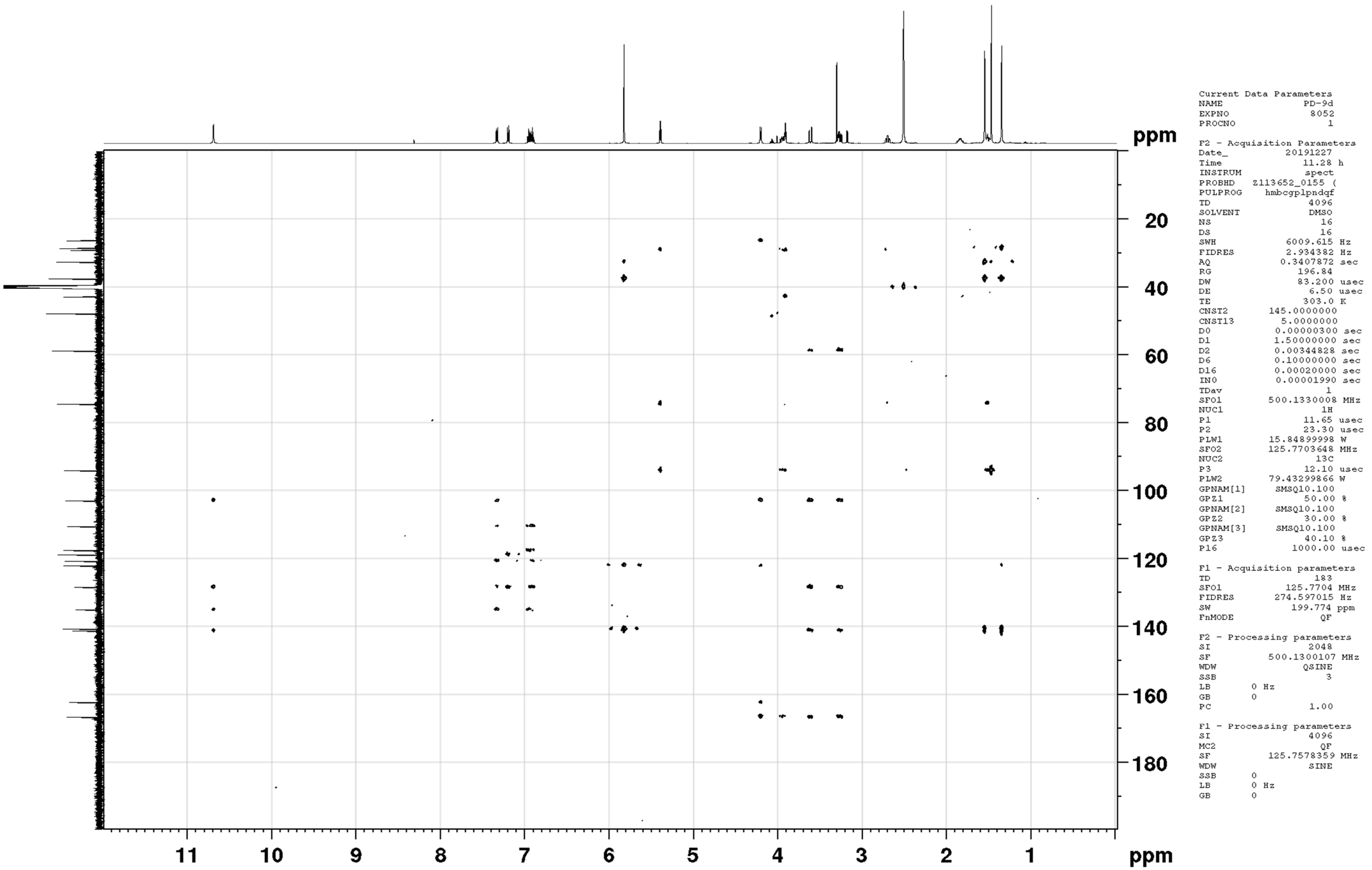

Figure S26. HMBC spectrum (500 MHz, DMSO-d6) of **1**

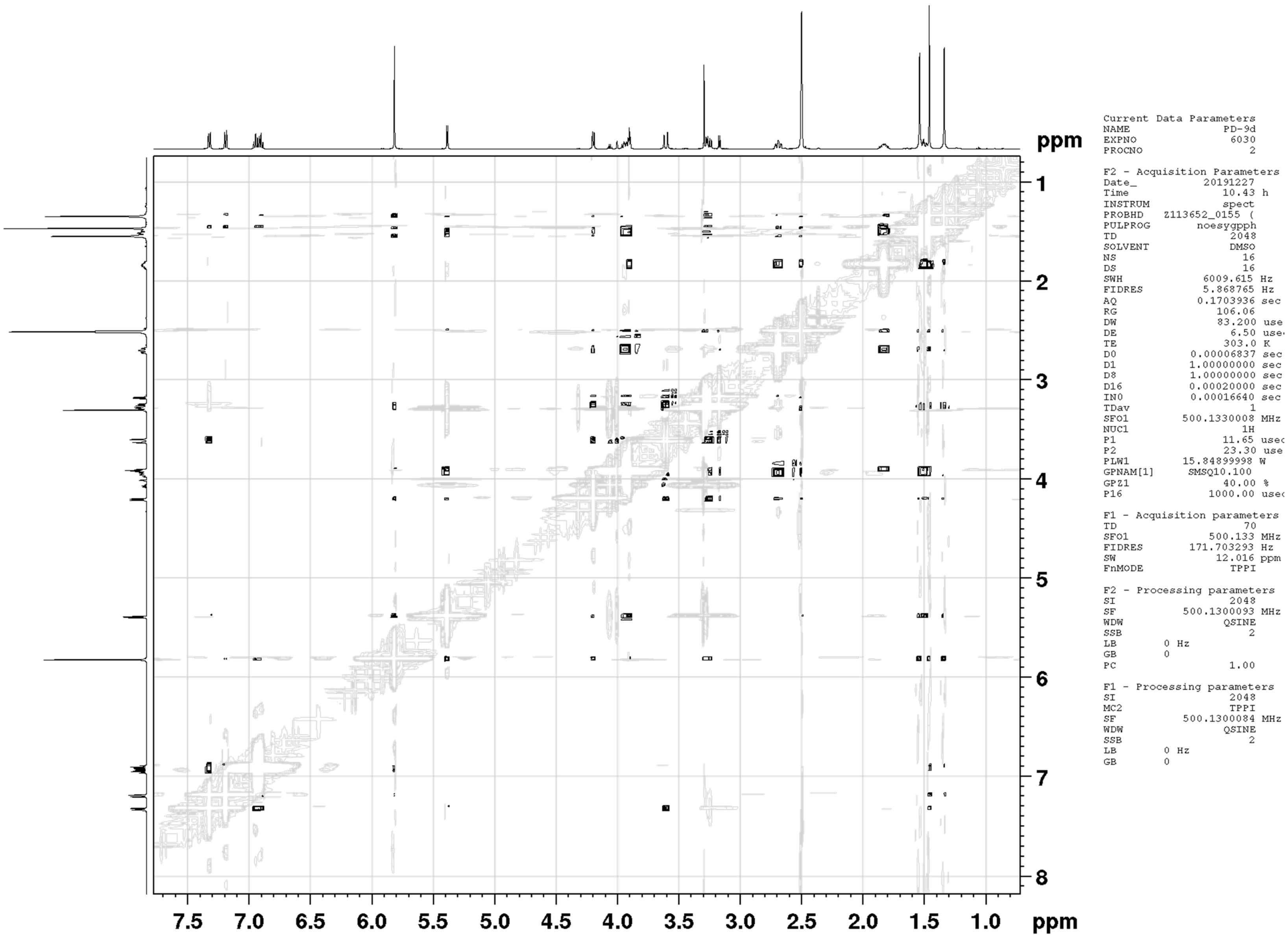

Figure S27. NOESY spectrum (500 MHz, DMSO-d6) of **1**

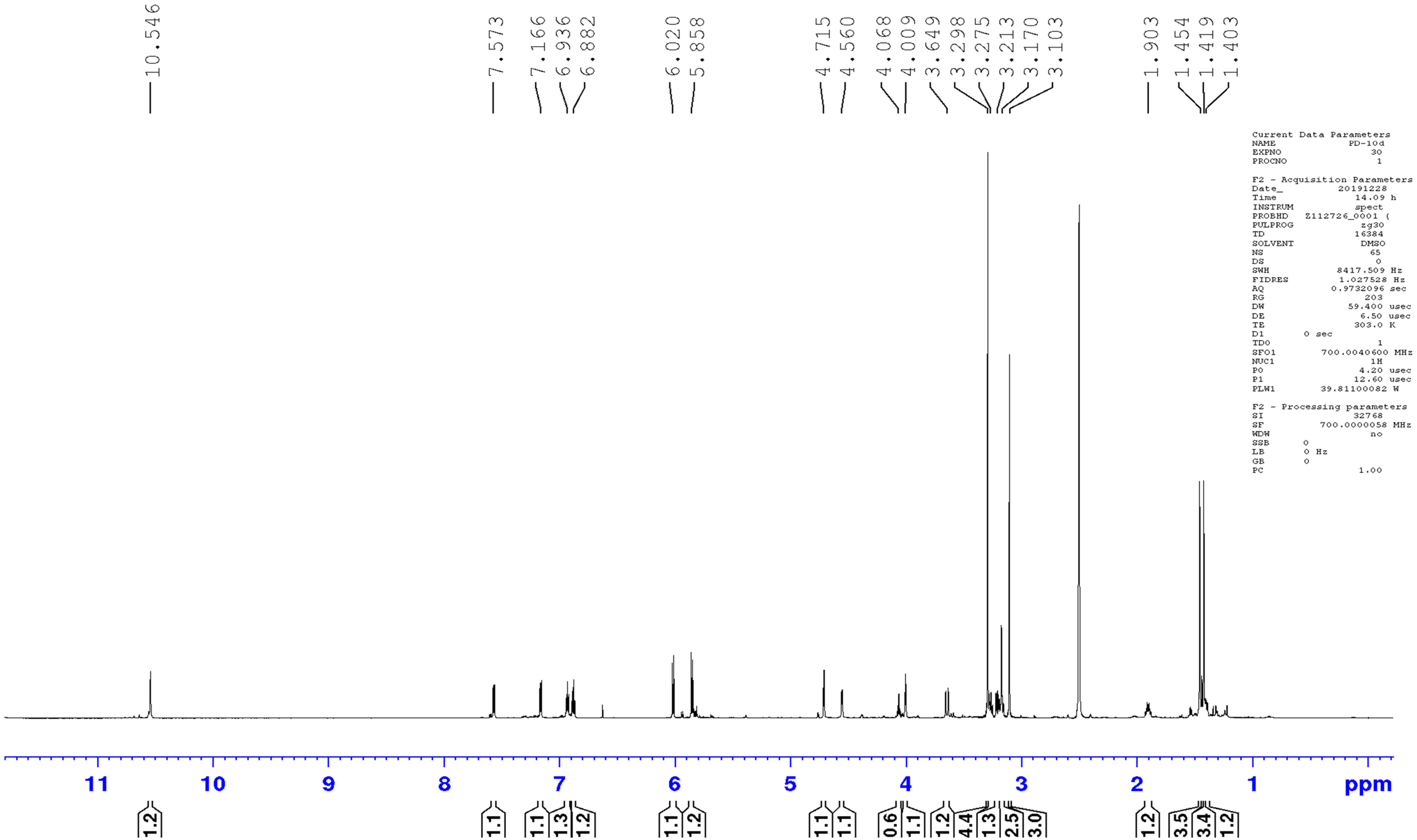

Figure S28. ^{1}H NMR spectrum (700 MHz, DMSO-d6) of **2**

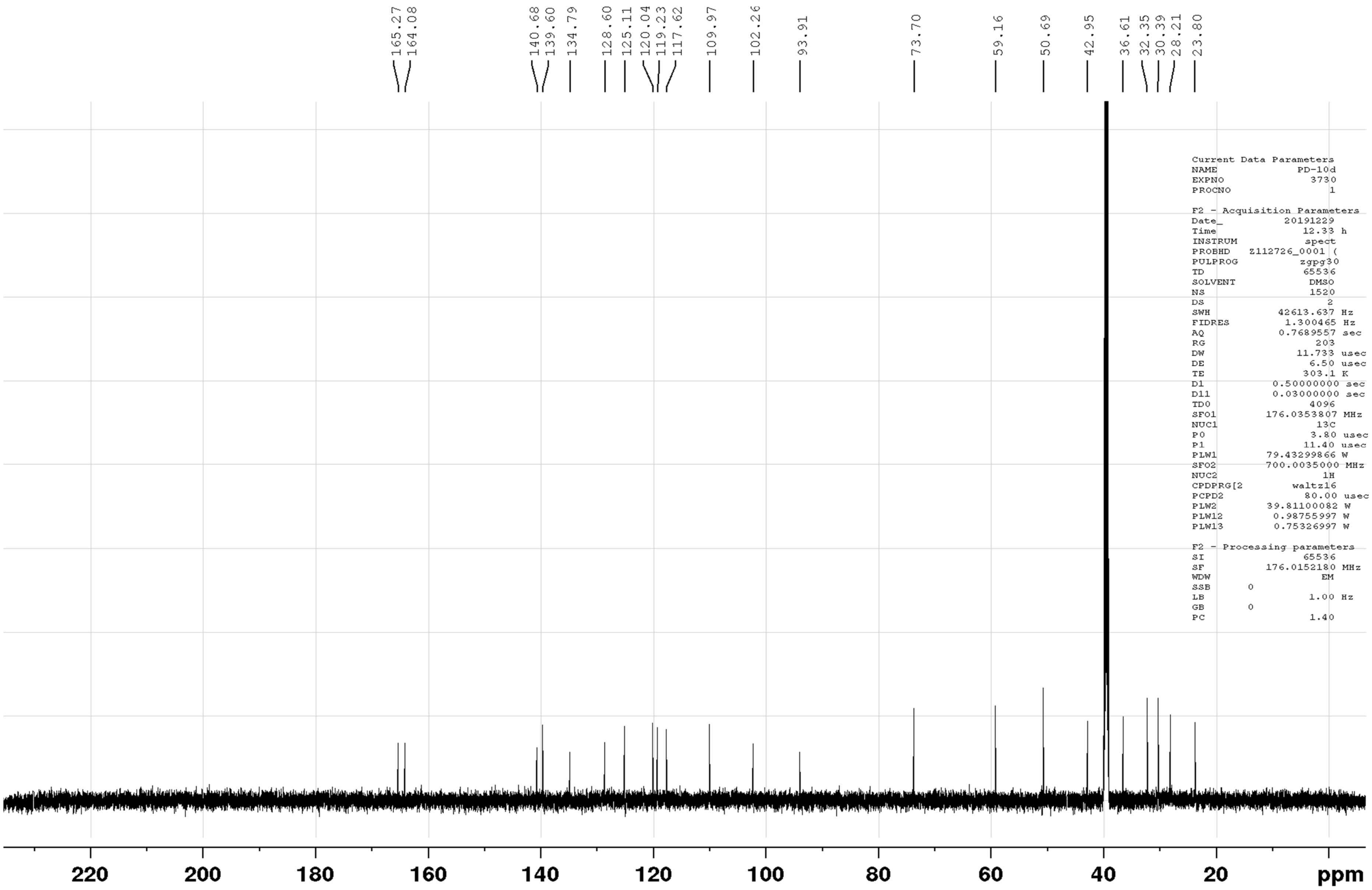

Figure S29. ^{13}C NMR spectrum (176 MHz, DMSO-d6) of **2**

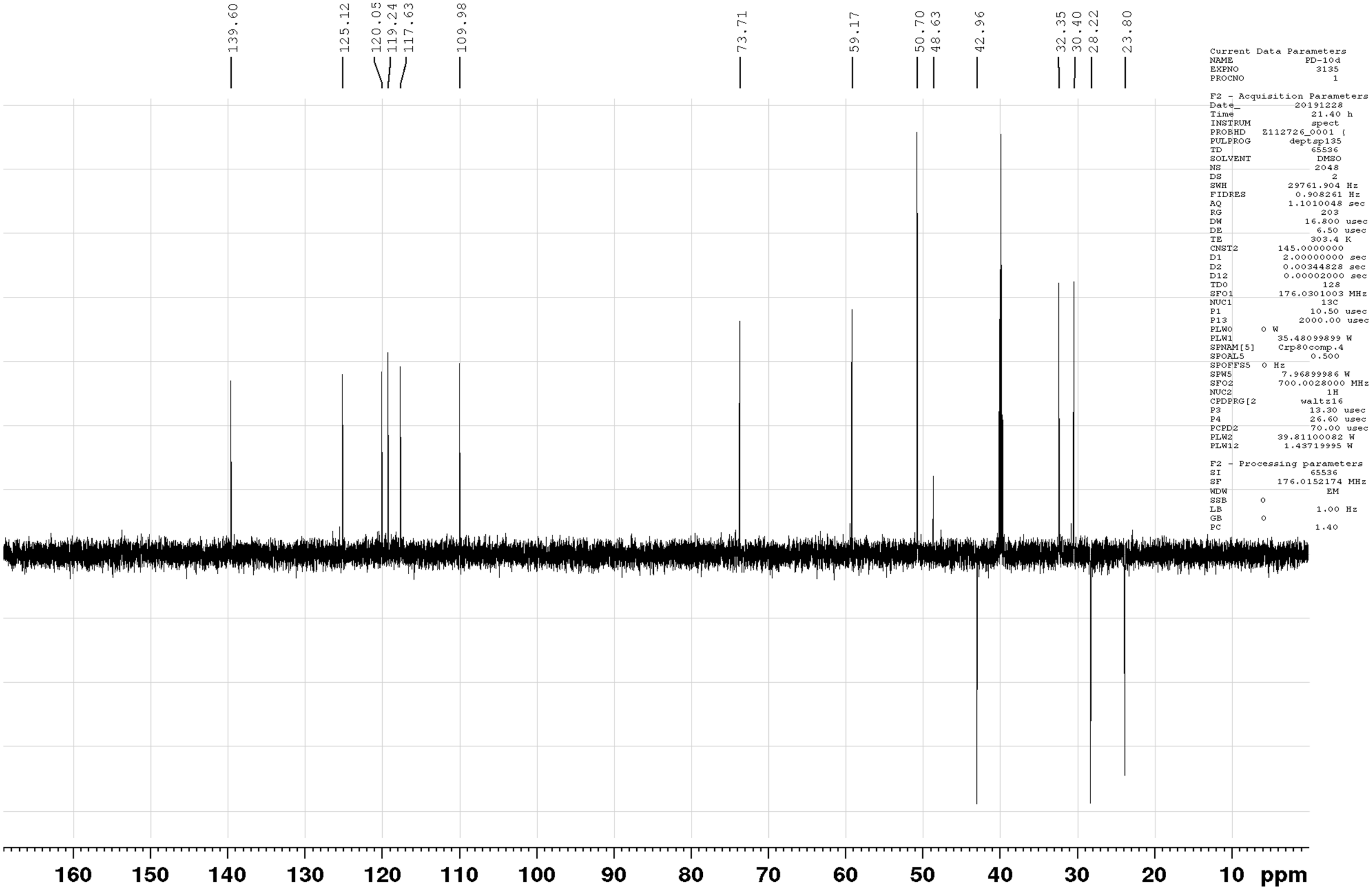

Figure S30. DEPT-135 NMR spectrum (176 MHz, DMSO-d6) of **2**

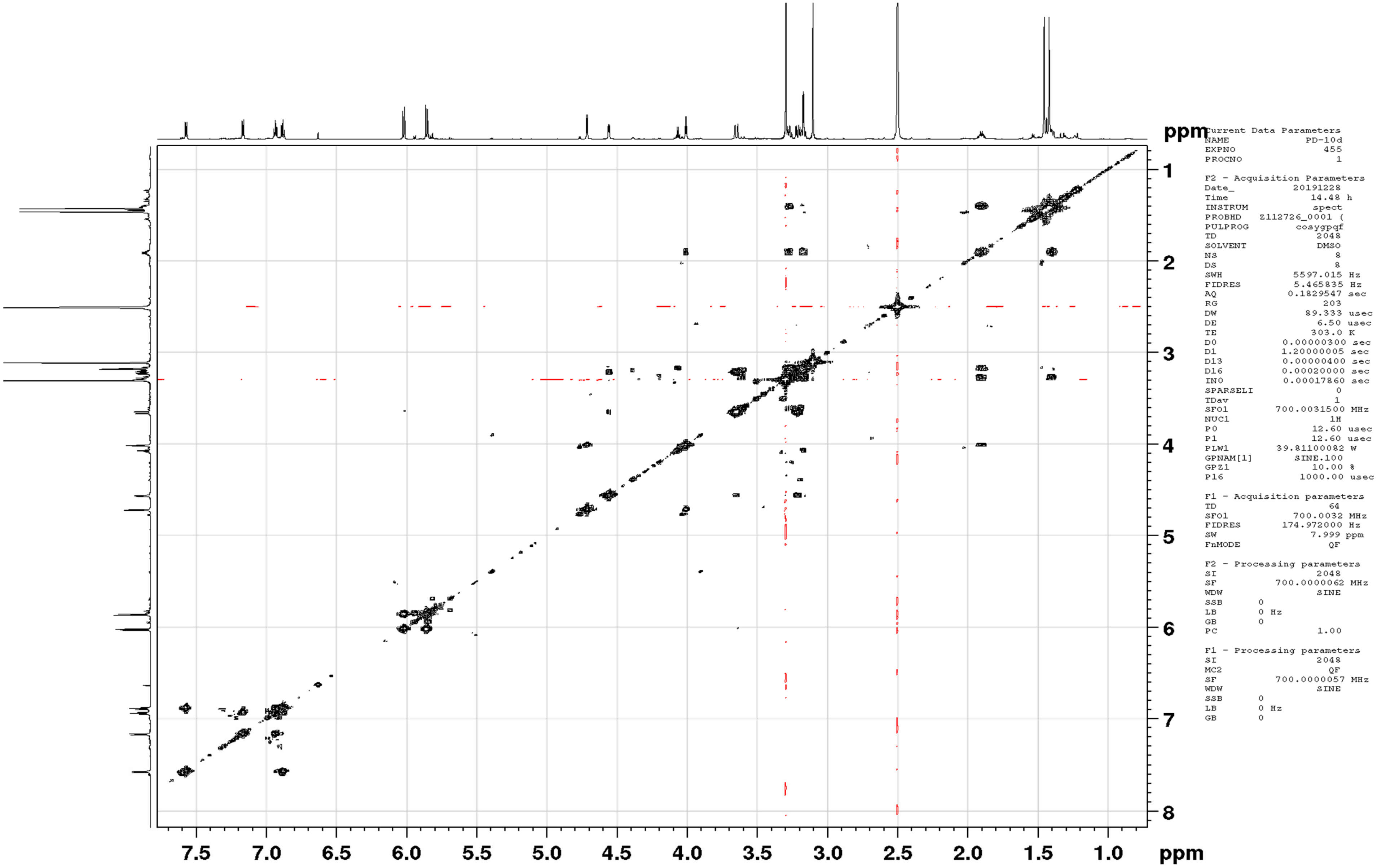

Figure S31. COSY-45 spectrum (700 MHz, DMSO-d6) of **2**

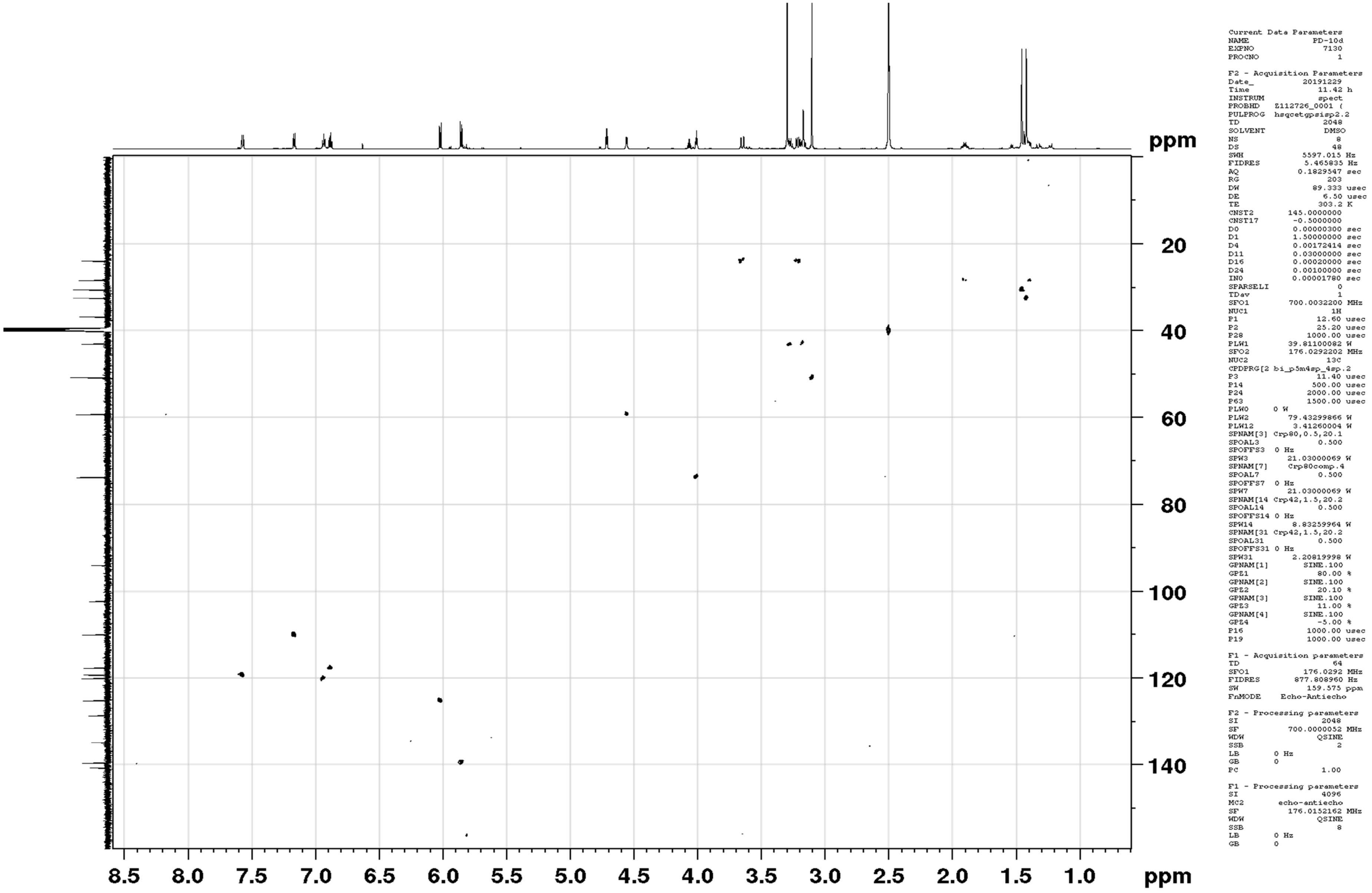

Figure S32. HSQC spectrum (700 MHz, DMSO-d6) of **2**

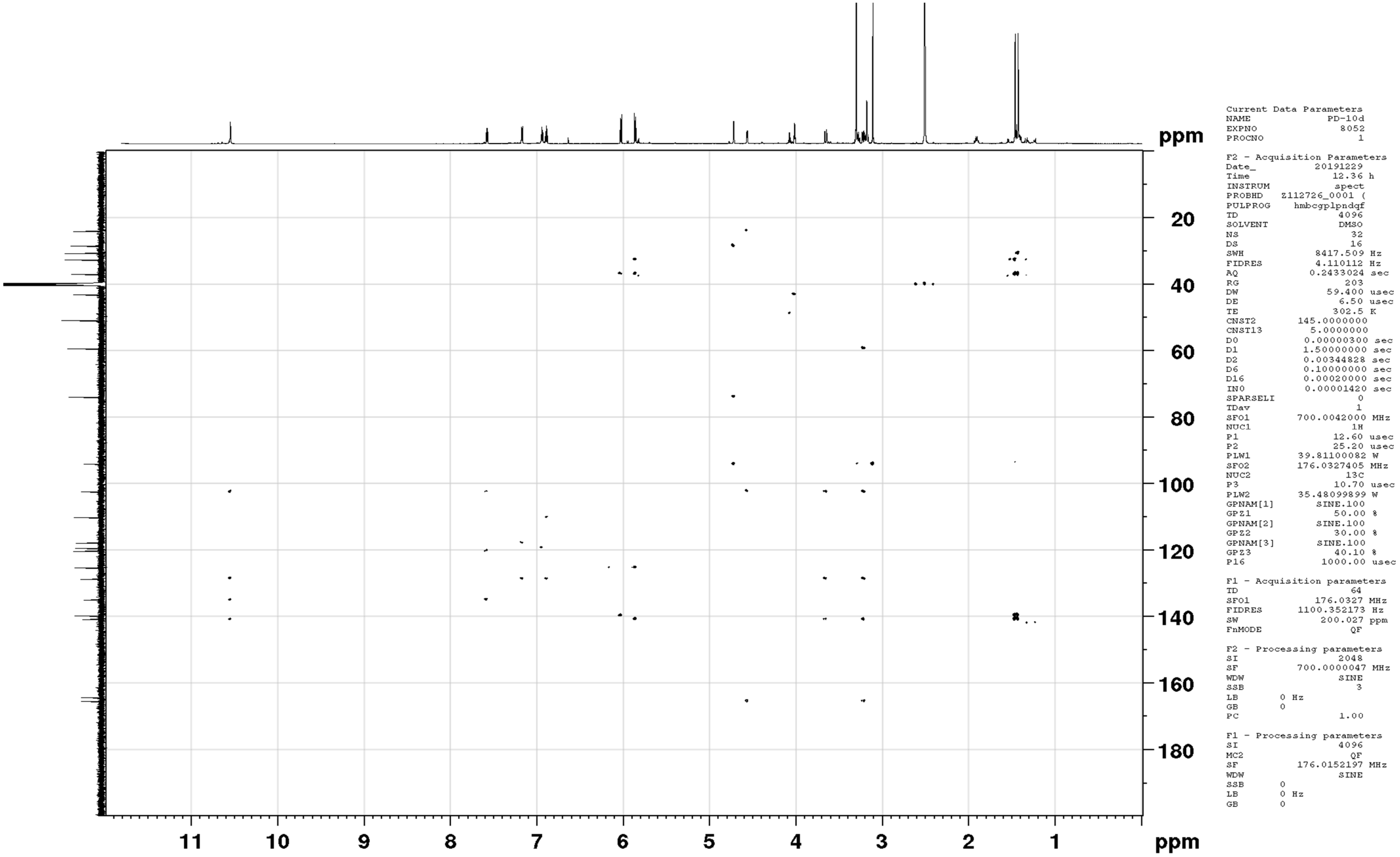

Figure S33. HMBC spectrum (700 MHz, DMSO-d6) of **2**

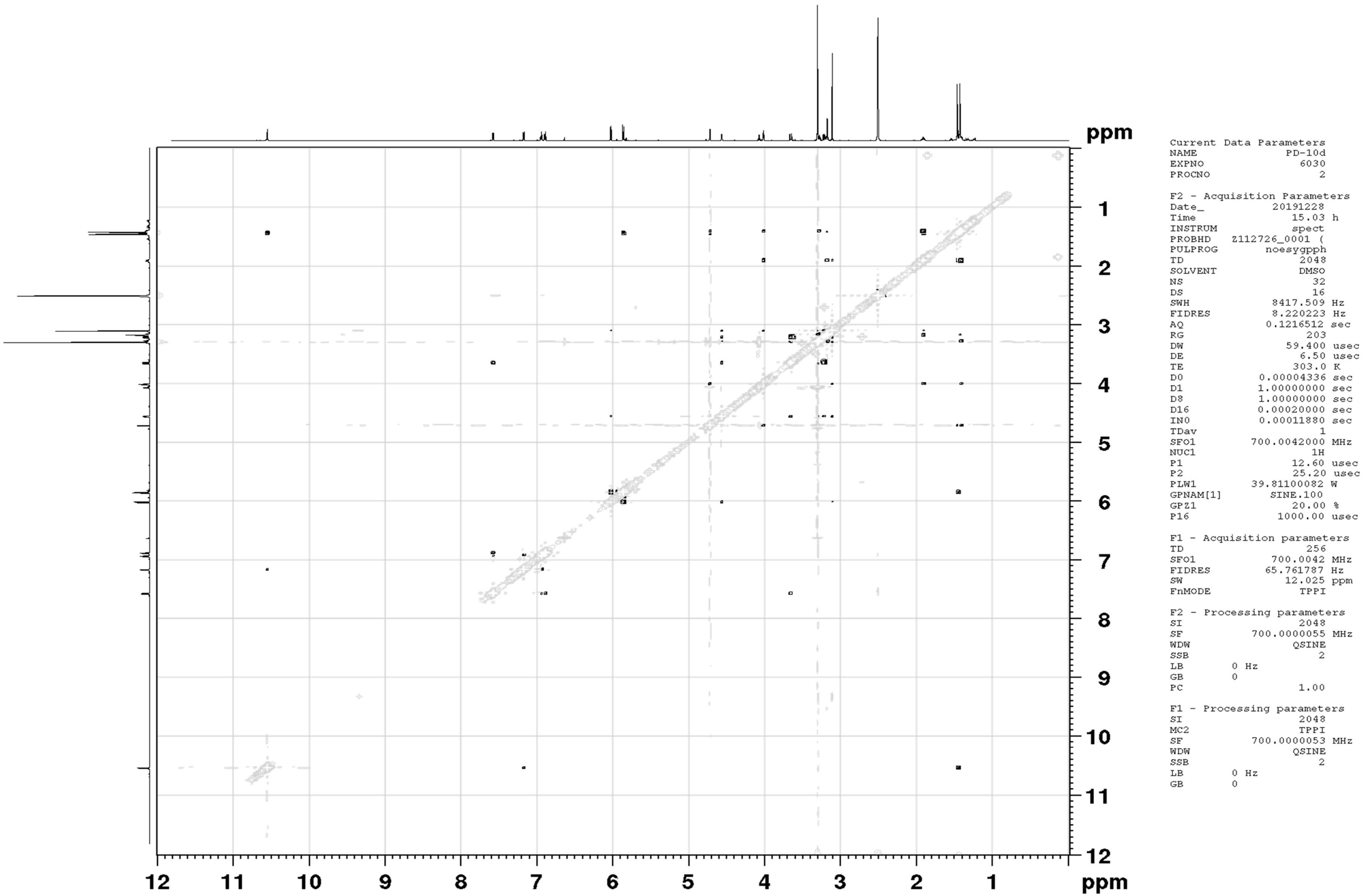

Figure S34. NOESY spectrum (700 MHz, DMSO-d6) of **2**

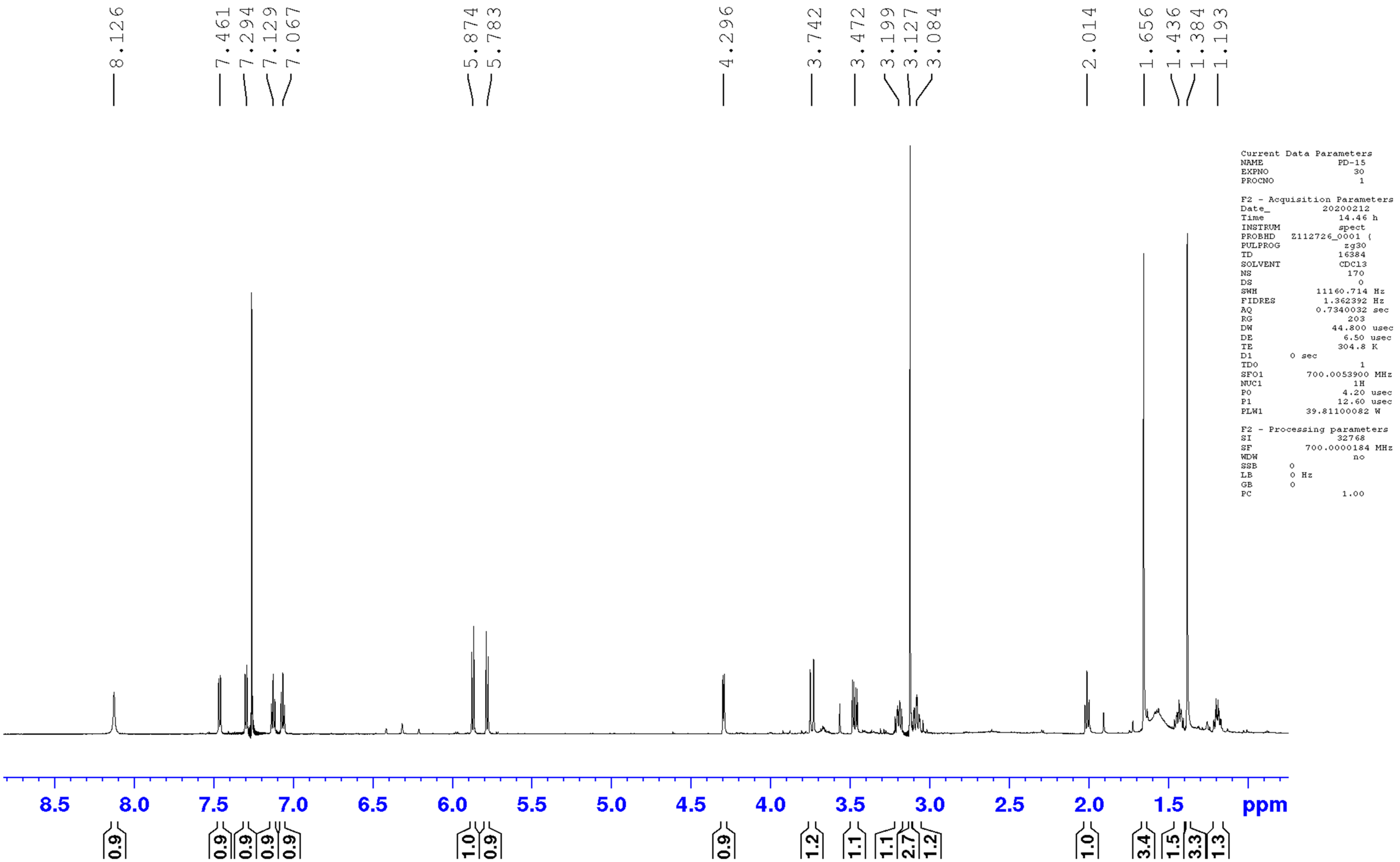

Figure S35. ^{1}H NMR spectrum (700 MHz, CD_3OD) of **3**

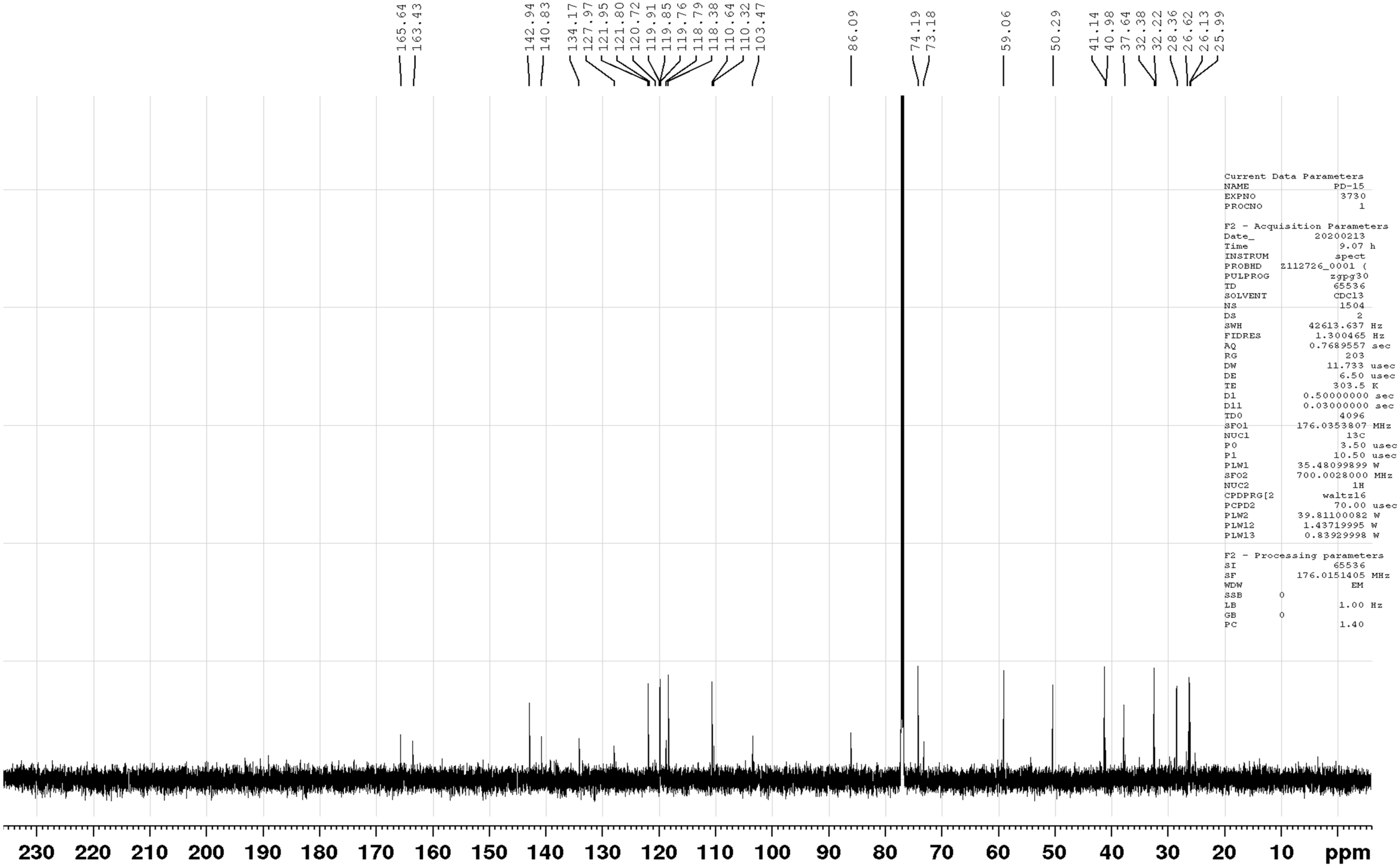

Figure S36. ^{13}C NMR spectrum (176 MHz, CD_3OD) of **3**

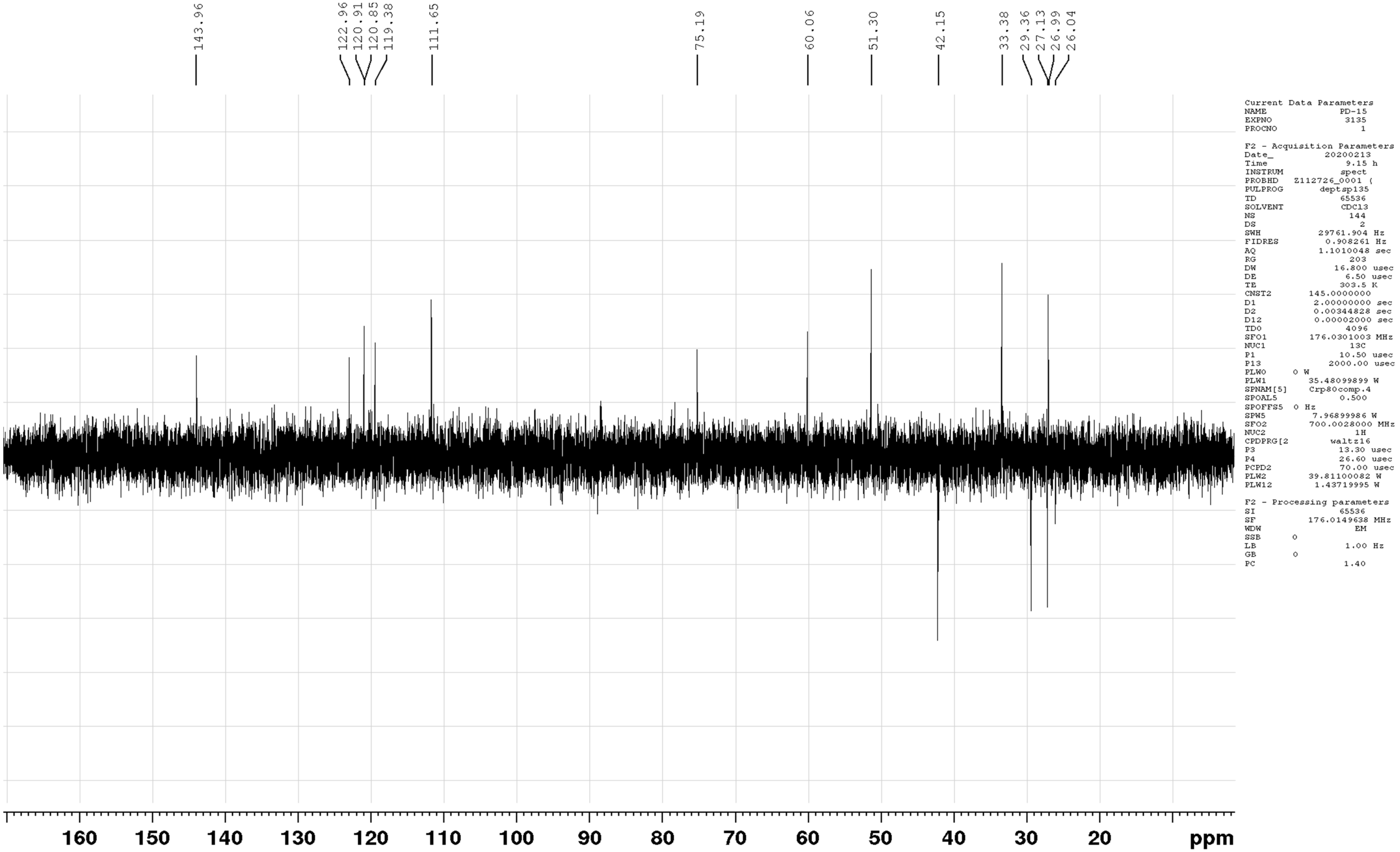

Figure S37. DEPT-135 NMR spectrum (176 MHz, CD_3OD) of **3**

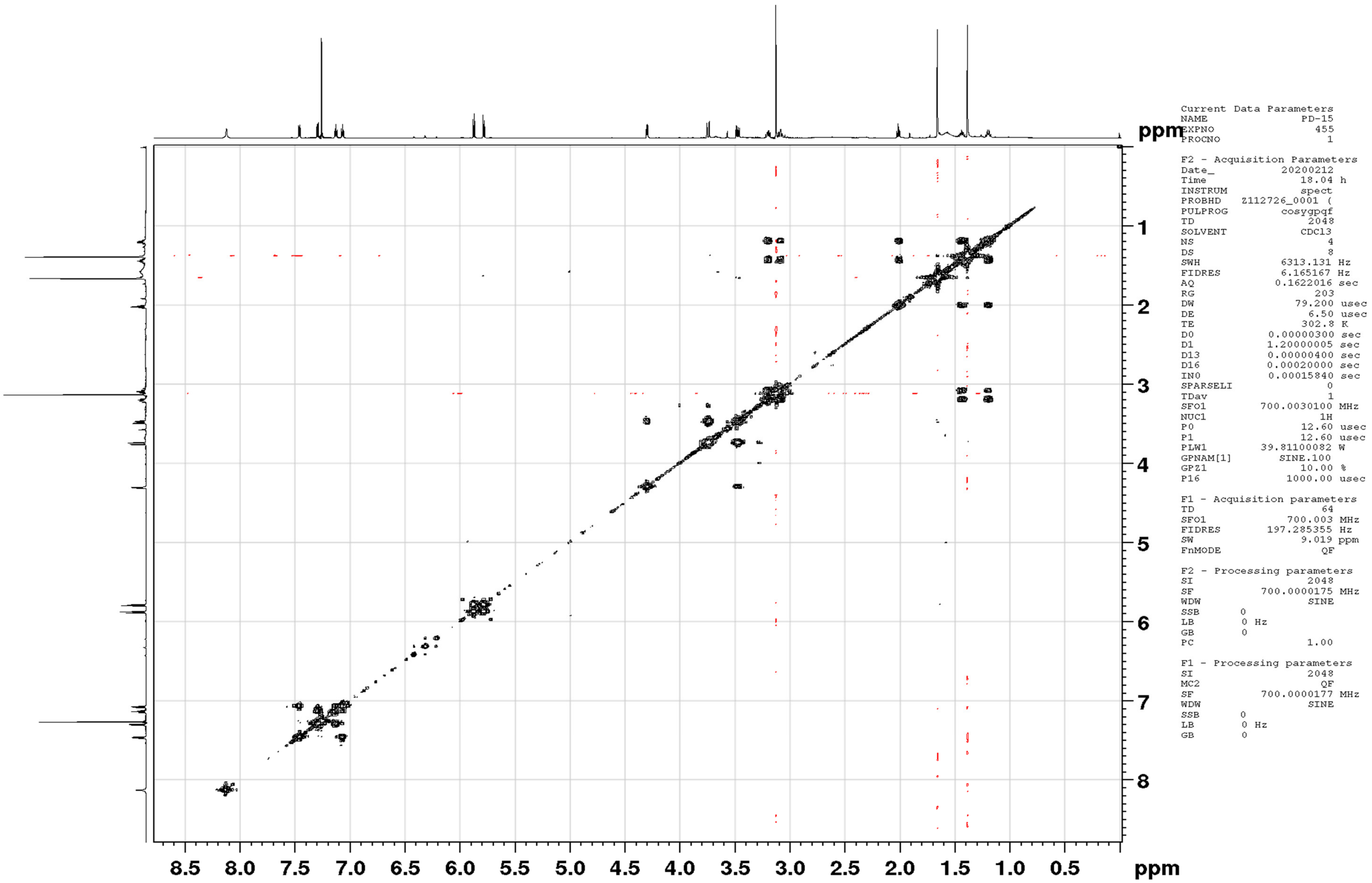

Figure S38. COSY-45 spectrum (700 MHz, CD_3OD) of **3**

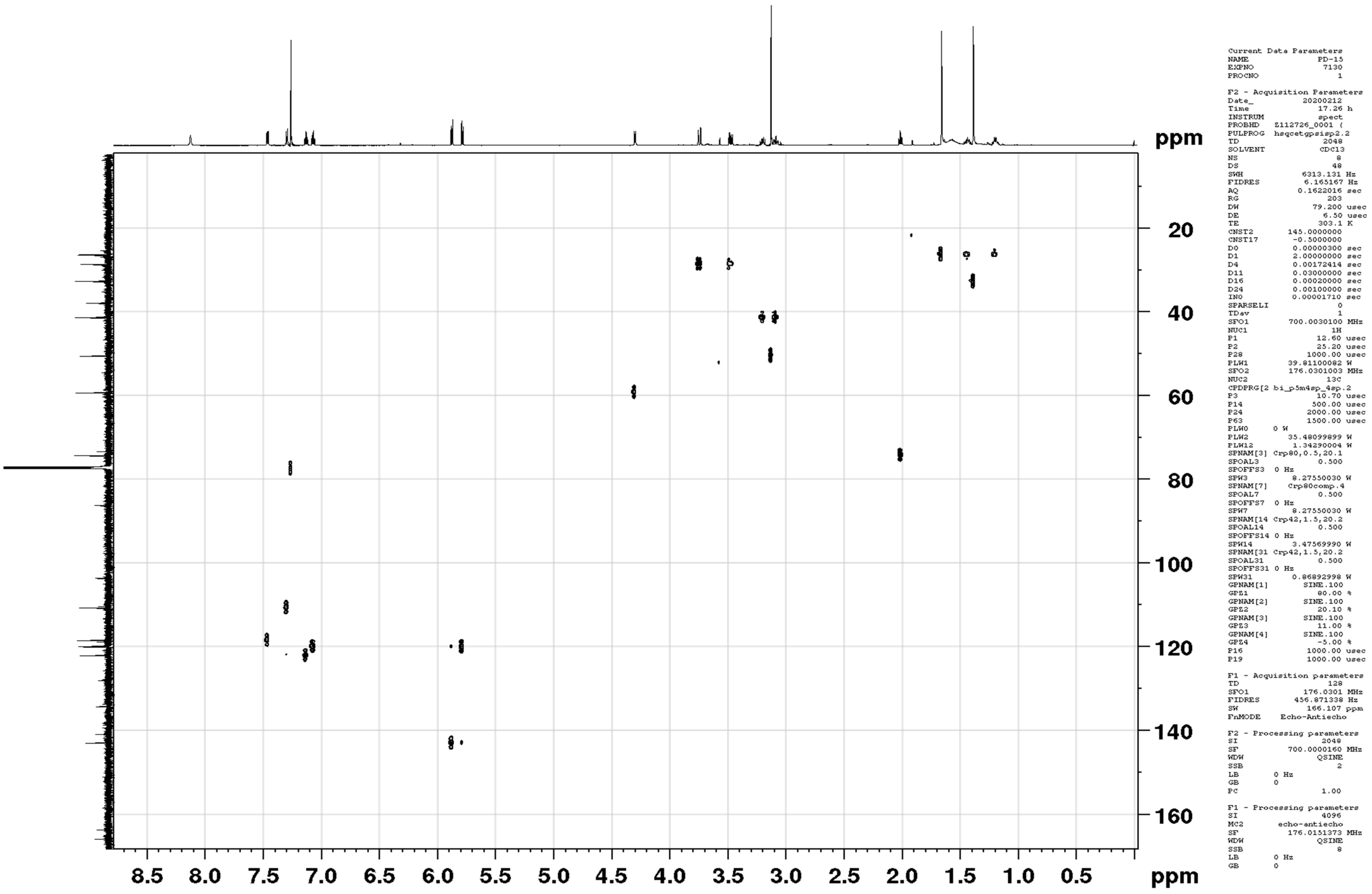

Figure S39. HSQC spectrum (700 MHz, CD_3OD) of **3**

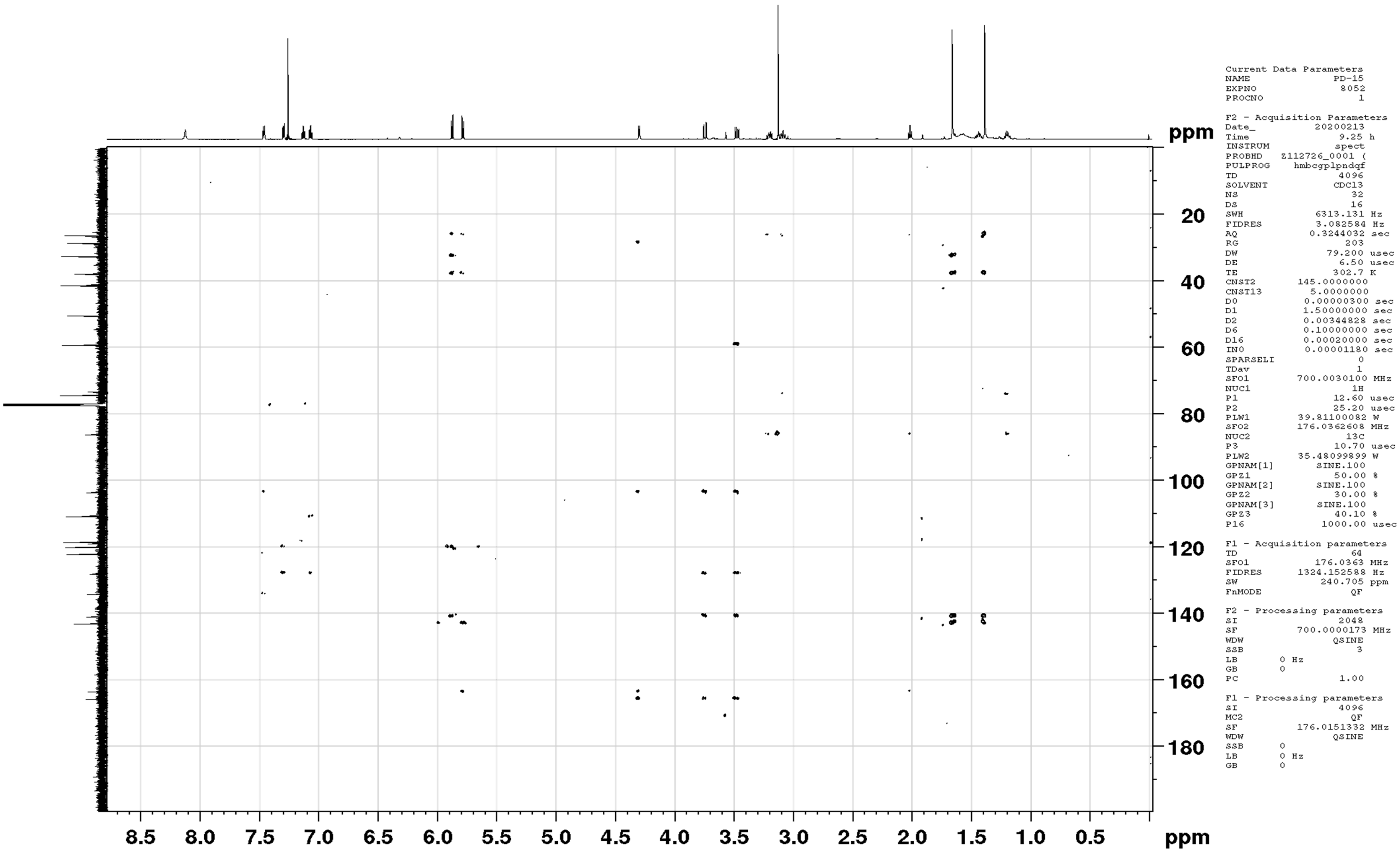

Figure S40. HMBC spectrum (700 MHz, CD_3OD) of **3**

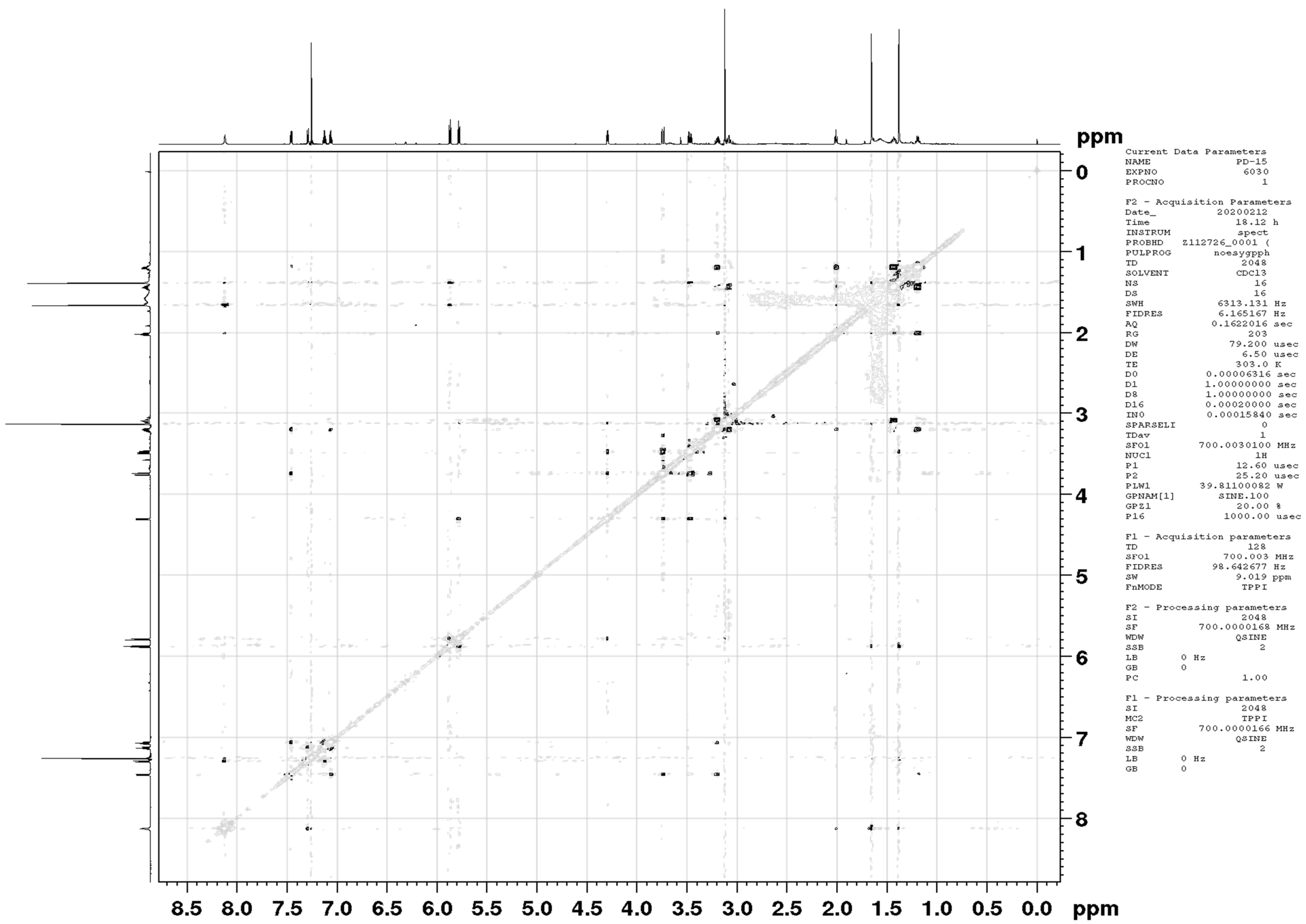

Figure S41. NOESY spectrum (700 MHz, CD_3OD) of **3**

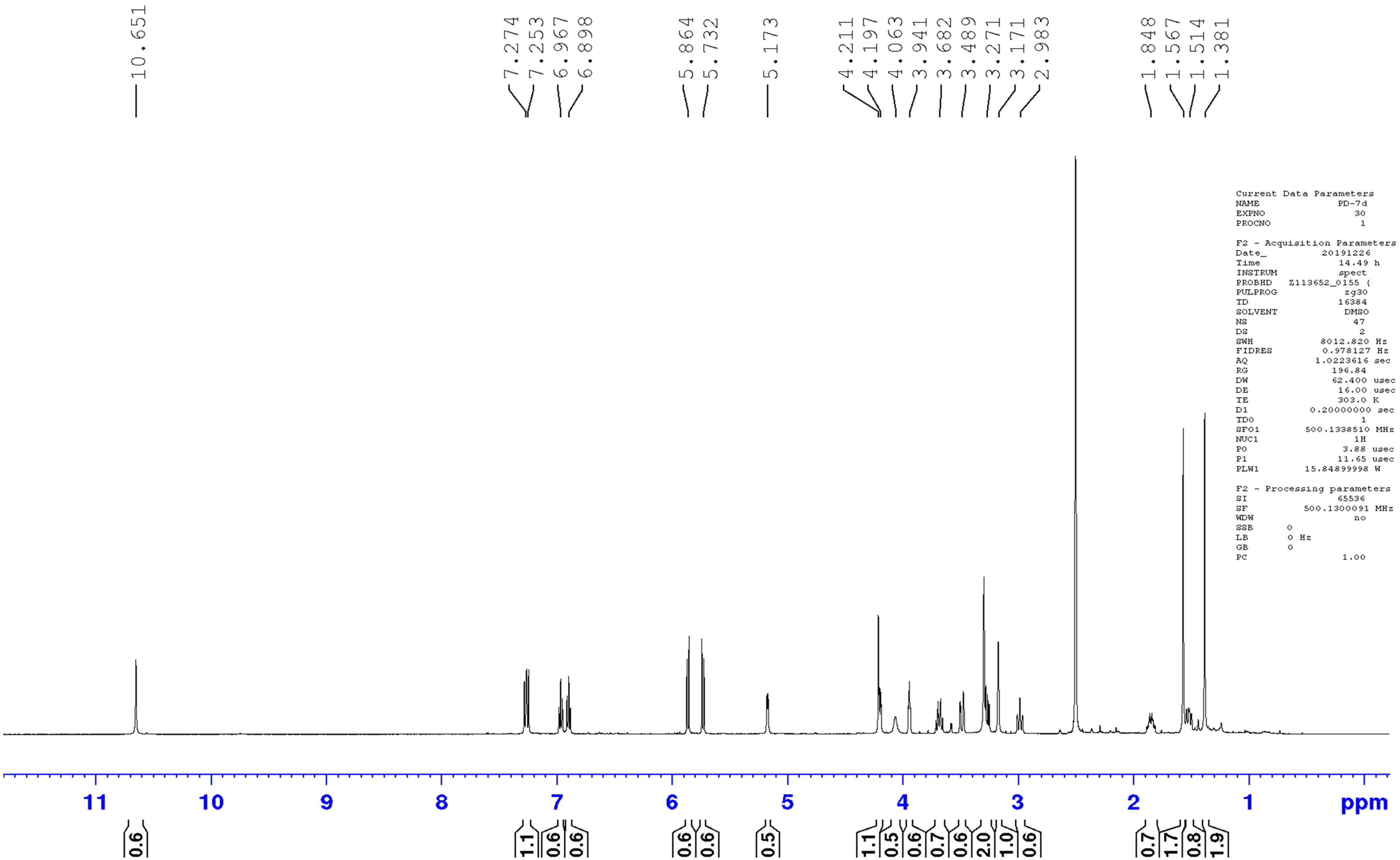

Figure S42. ^{1}H NMR spectrum (500 MHz, DMSO-d_6) of **4**

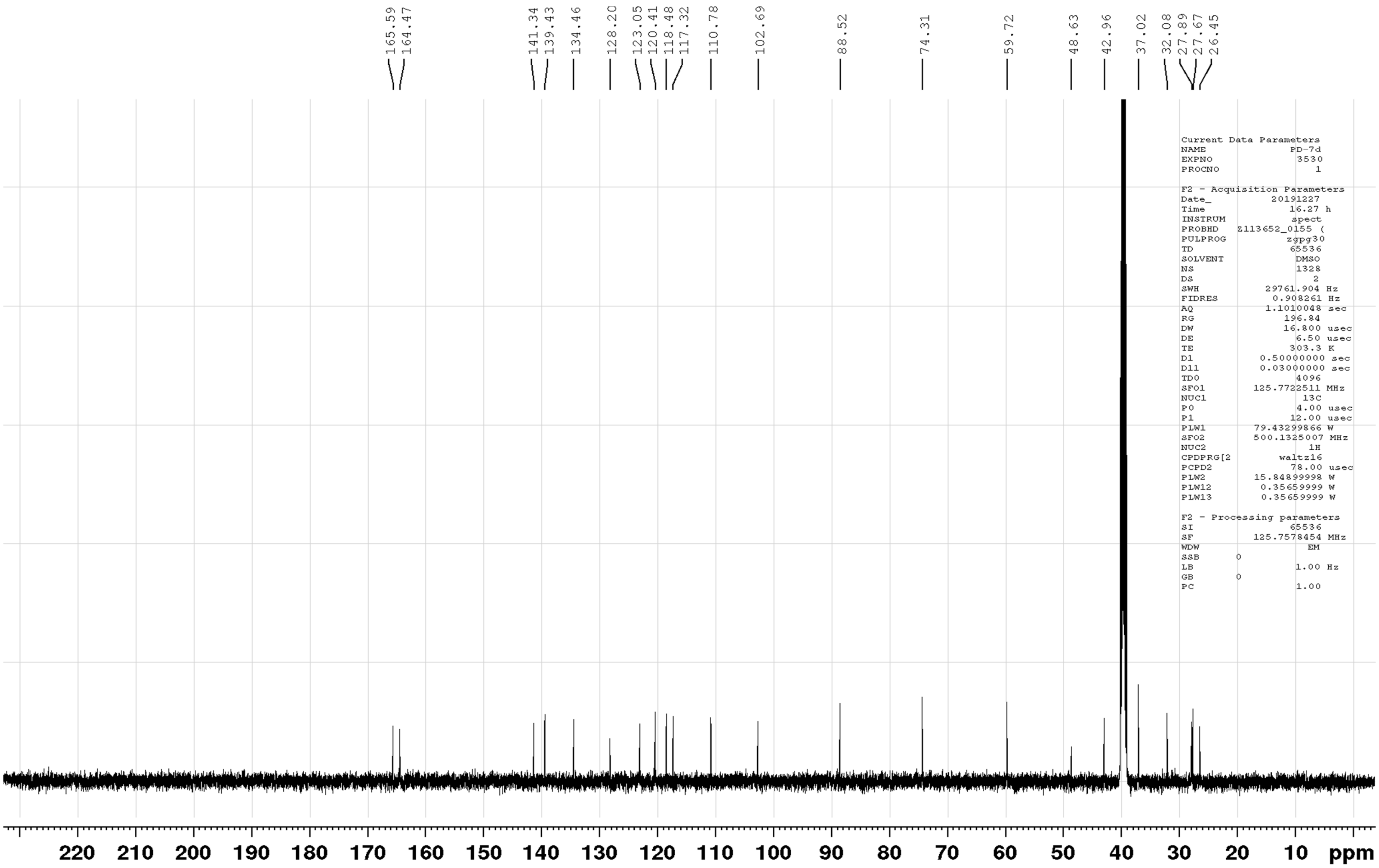

Figure S43. ^{13}C NMR spectrum (125 MHz, DMSO-d6) of **4**

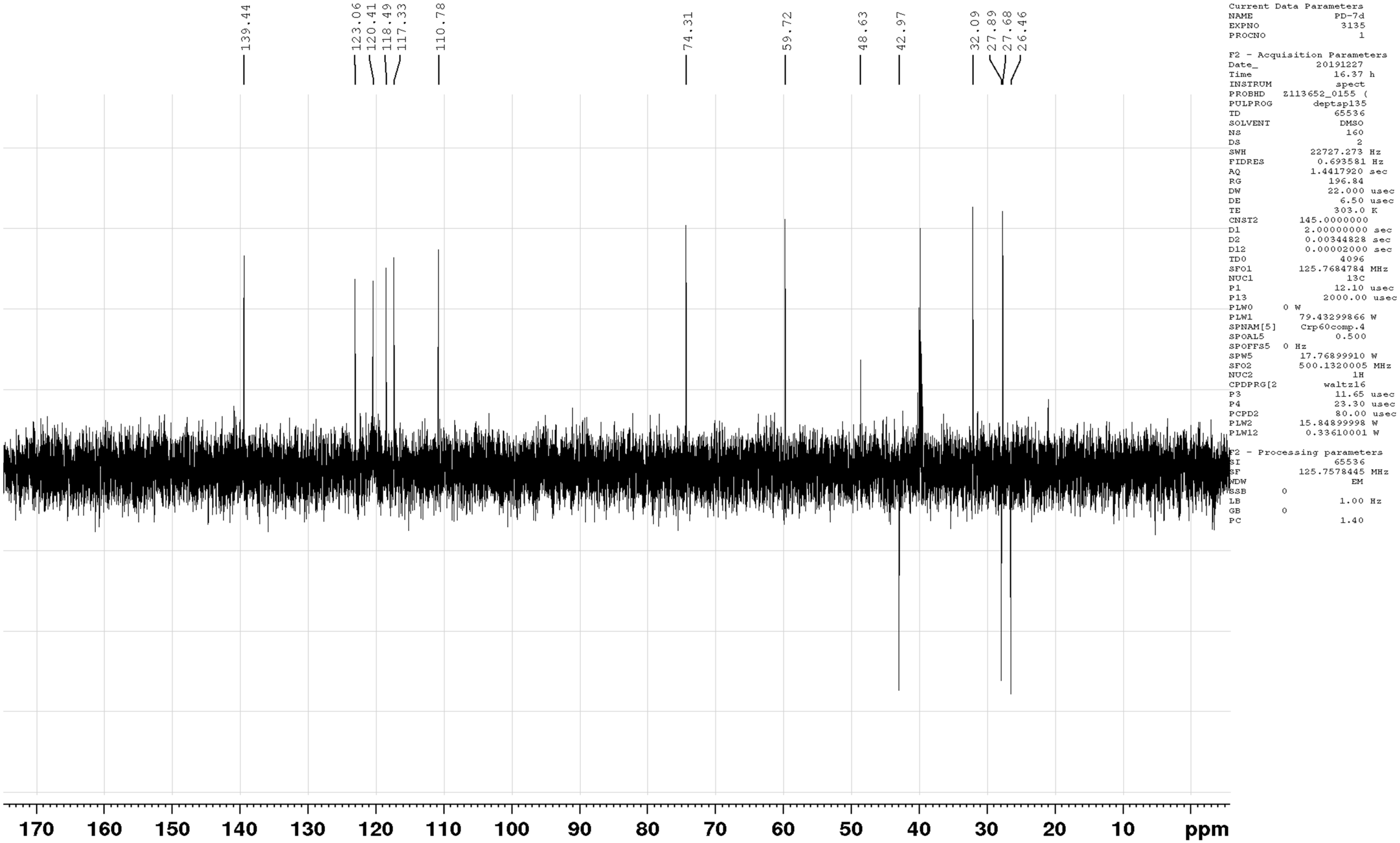

Figure S44. DEPT-135 NMR spectrum (125 MHz, DMSO-d_6) of **4**

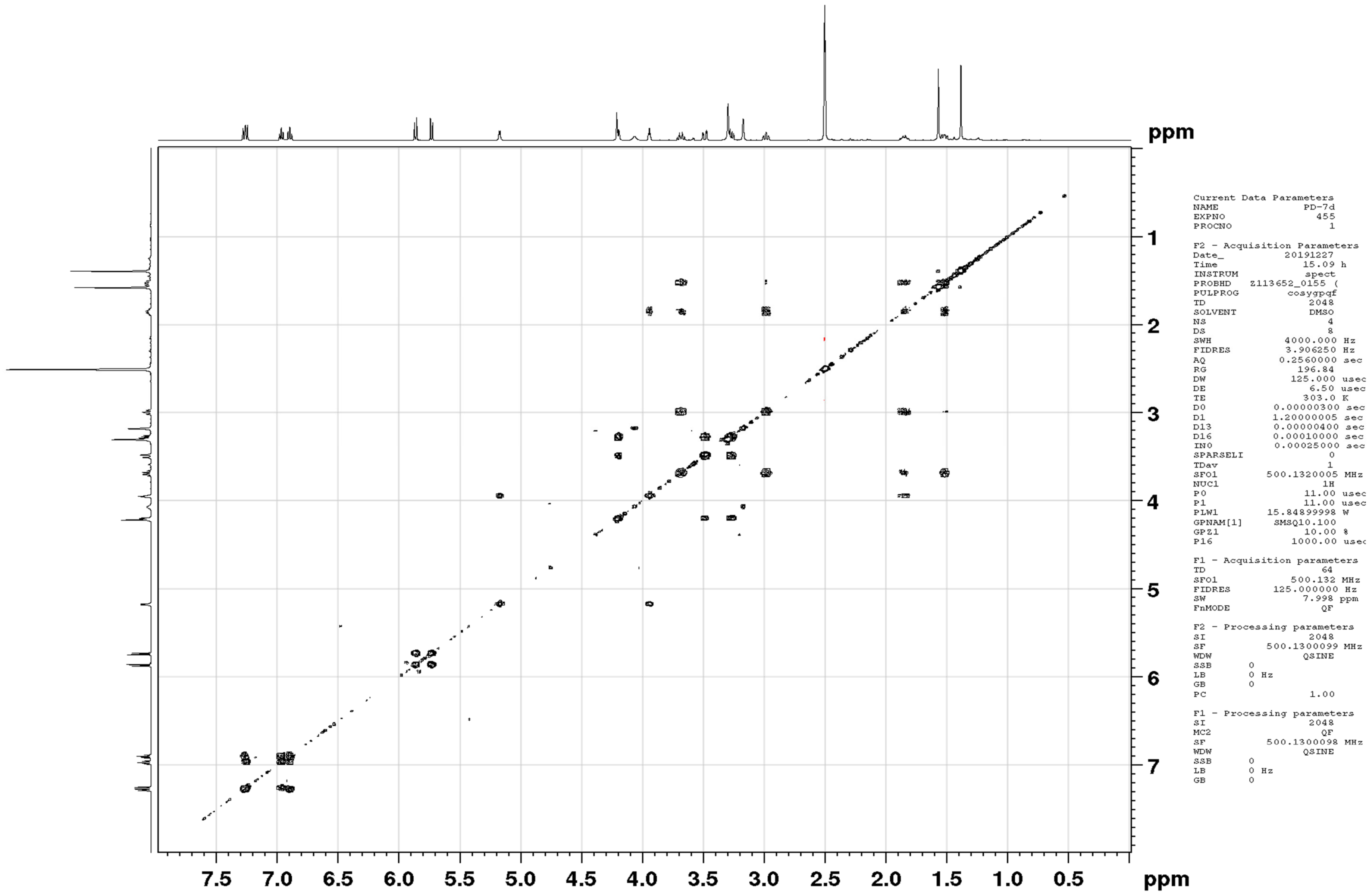

Figure S45. COSY-45 spectrum (500 MHz, DMSO-d_6) of **4**

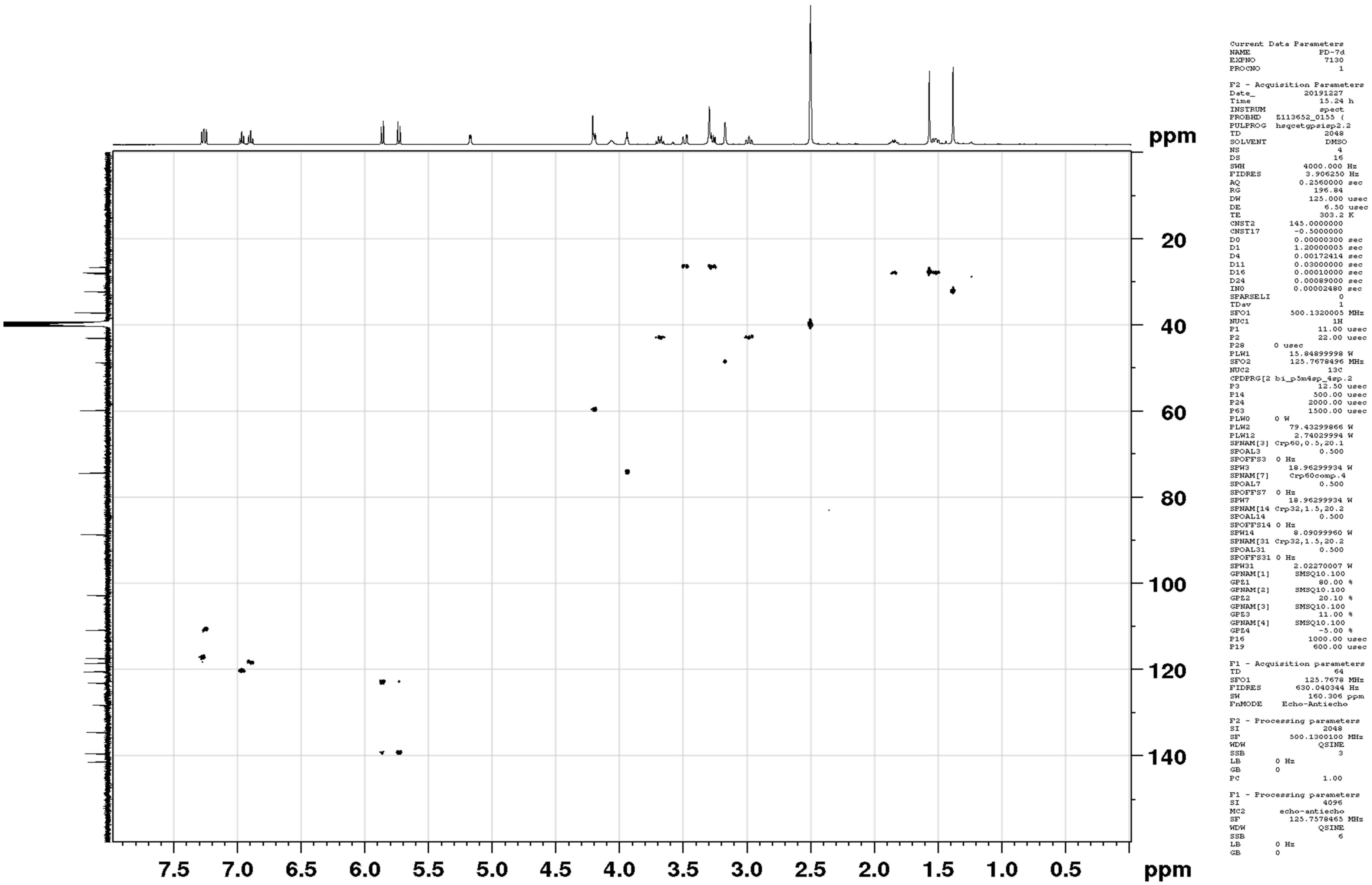

Figure S46. HSQC spectrum (500 MHz, DMSO-d_6) of **4**

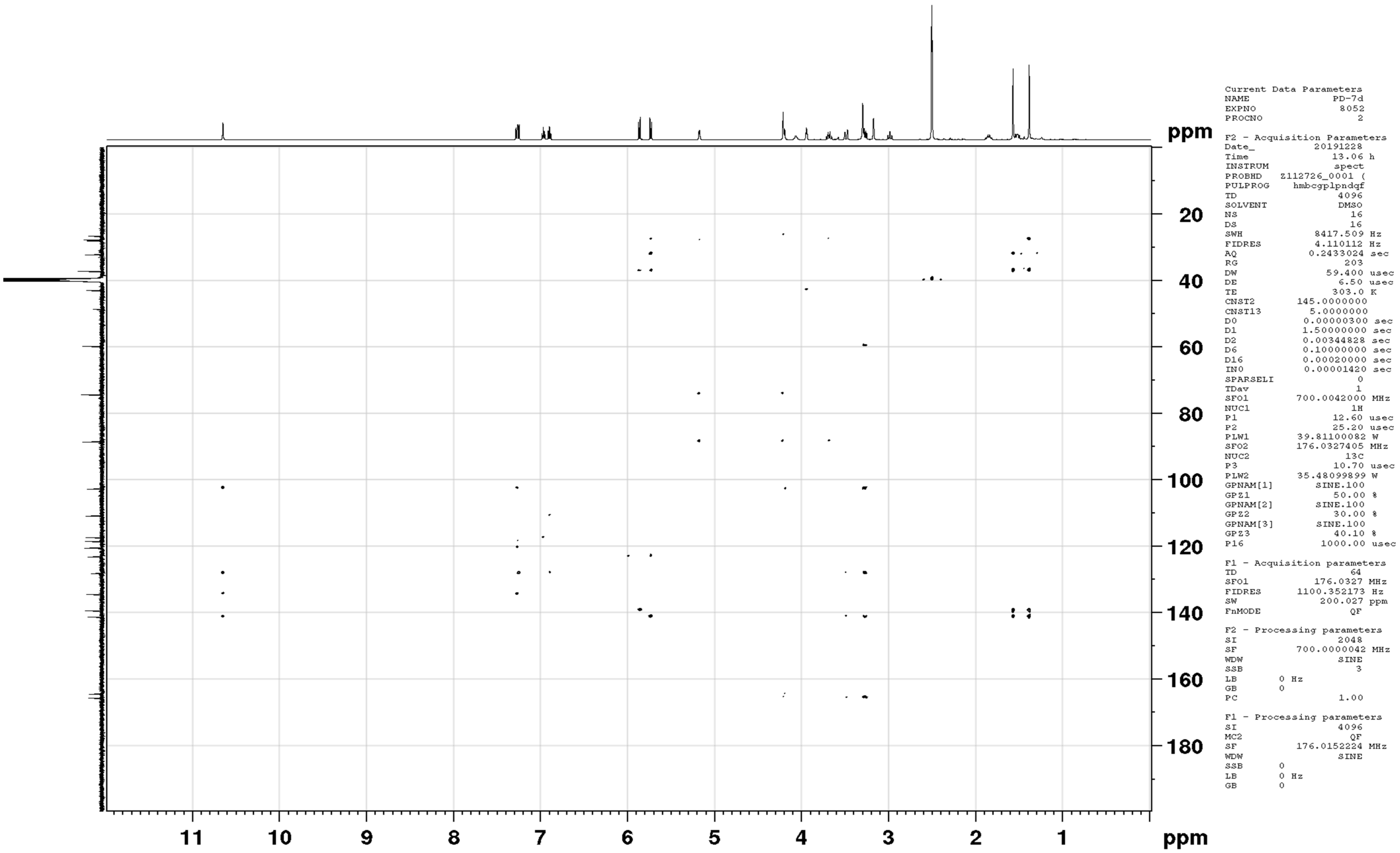

Figure S47. ^{1}H-^{13}C HMBC spectrum (700 MHz, DMSO-d_6) of **4**

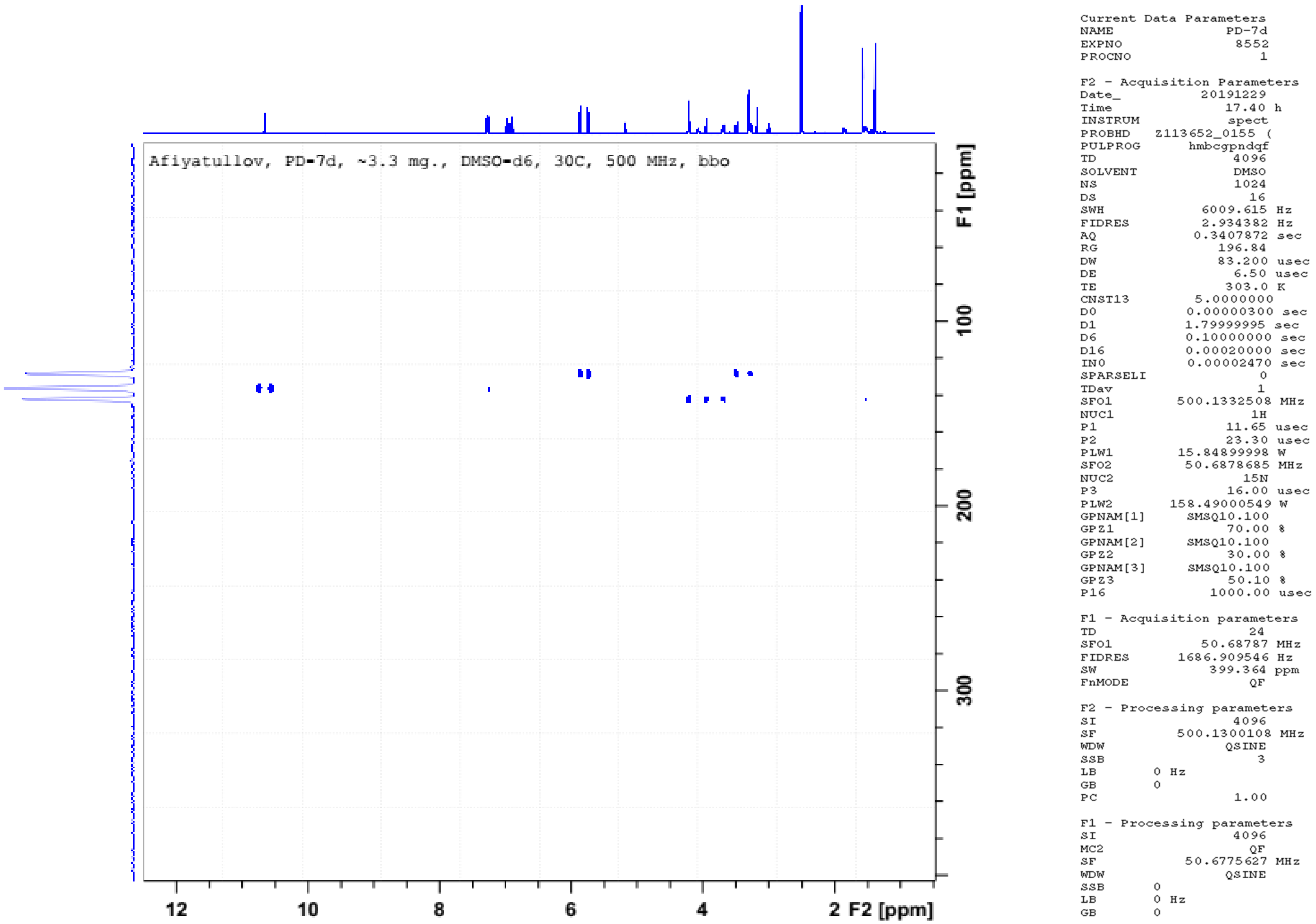

Figure S48. ^{1}H-^{15}N GHMBC spectrum (50 MHz, DMSO-d$_6$) of **4**

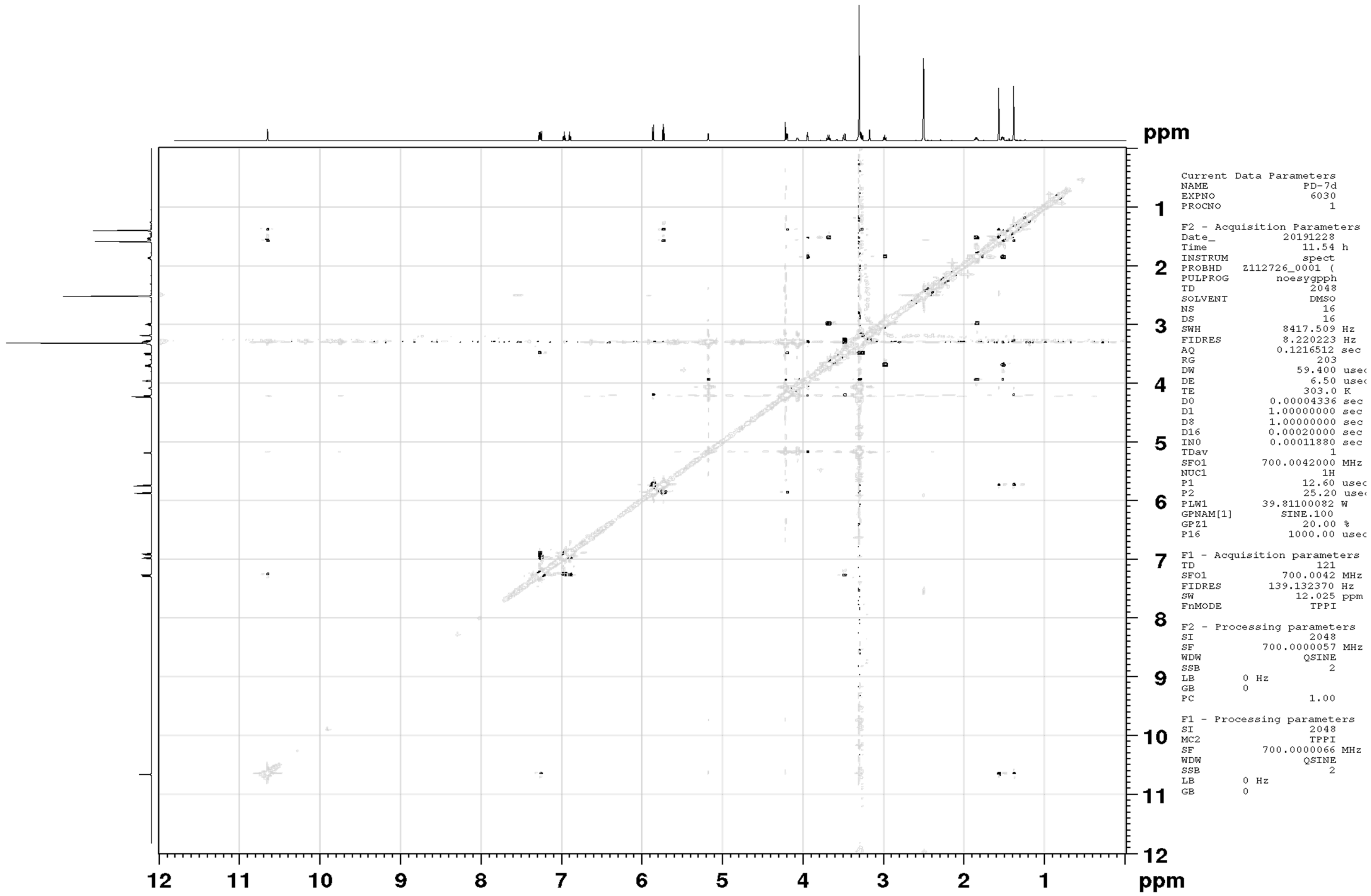

Figure S49. NOESY spectrum (700 MHz, DMSO-d_6) of **4**

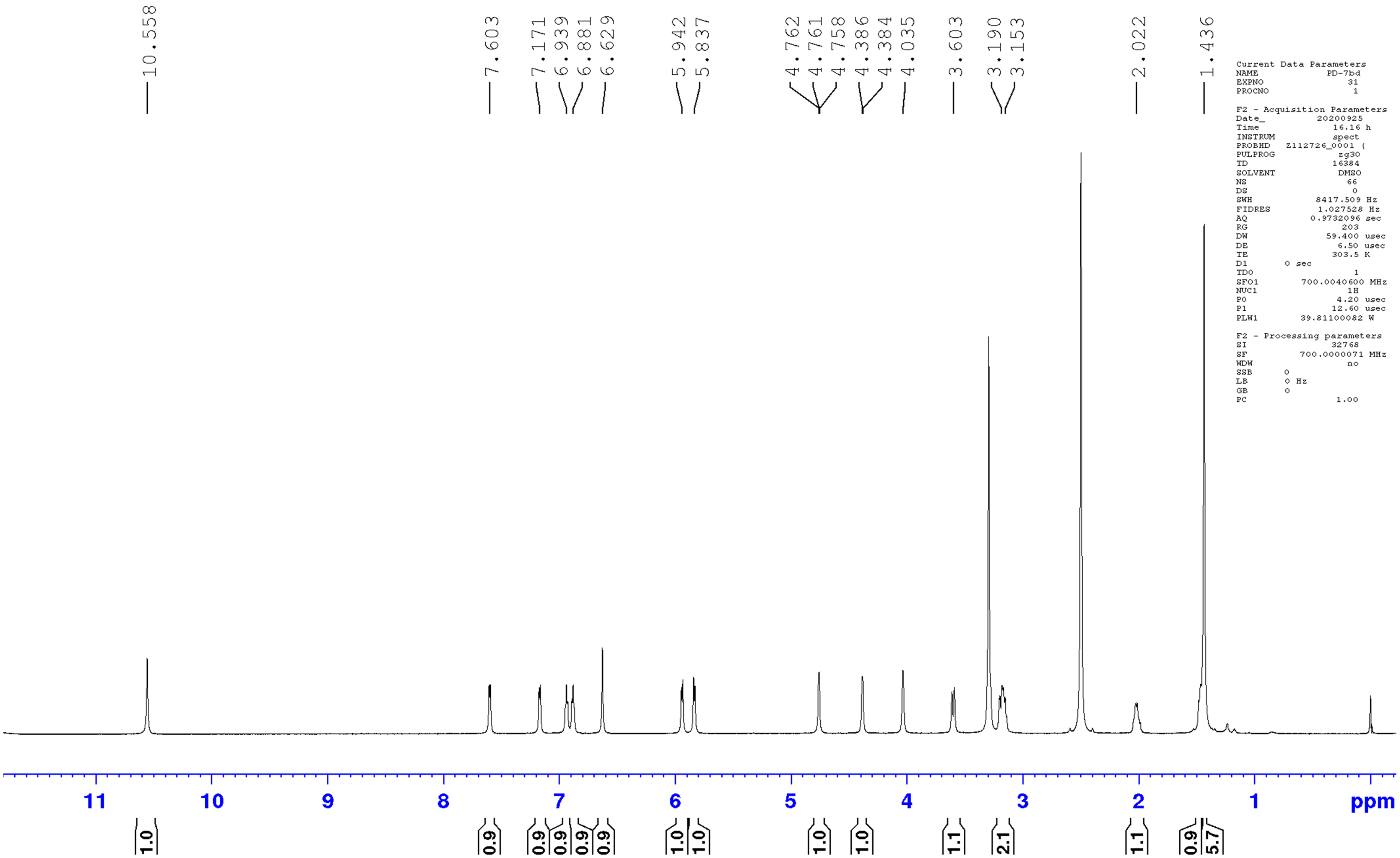

Figure S50. ^{1}H NMR spectrum (700 MHz, DMSO-d6) of **5**

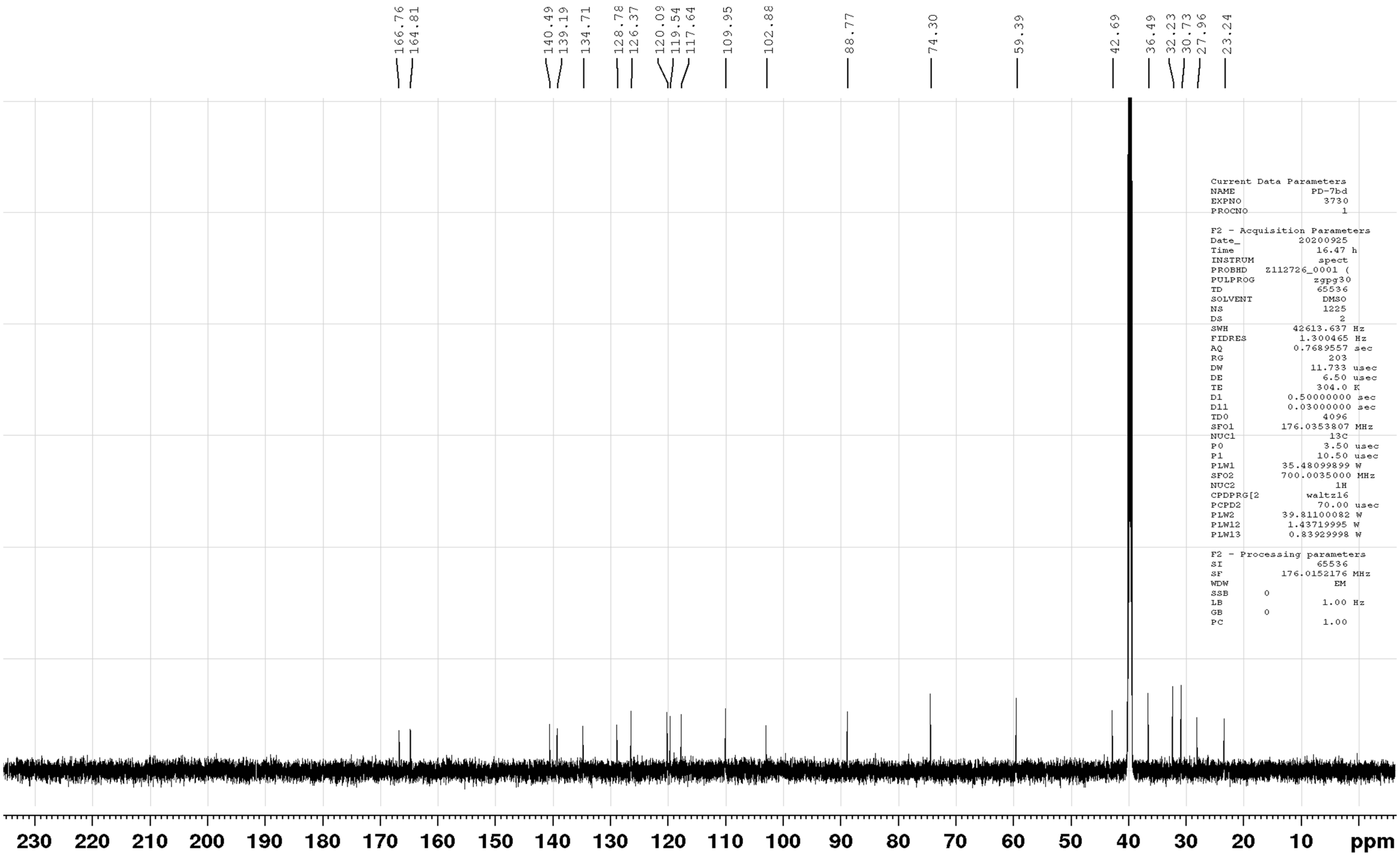

Figure S51. ^{13}C NMR spectrum (176 MHz, DMSO-d6) of **5**

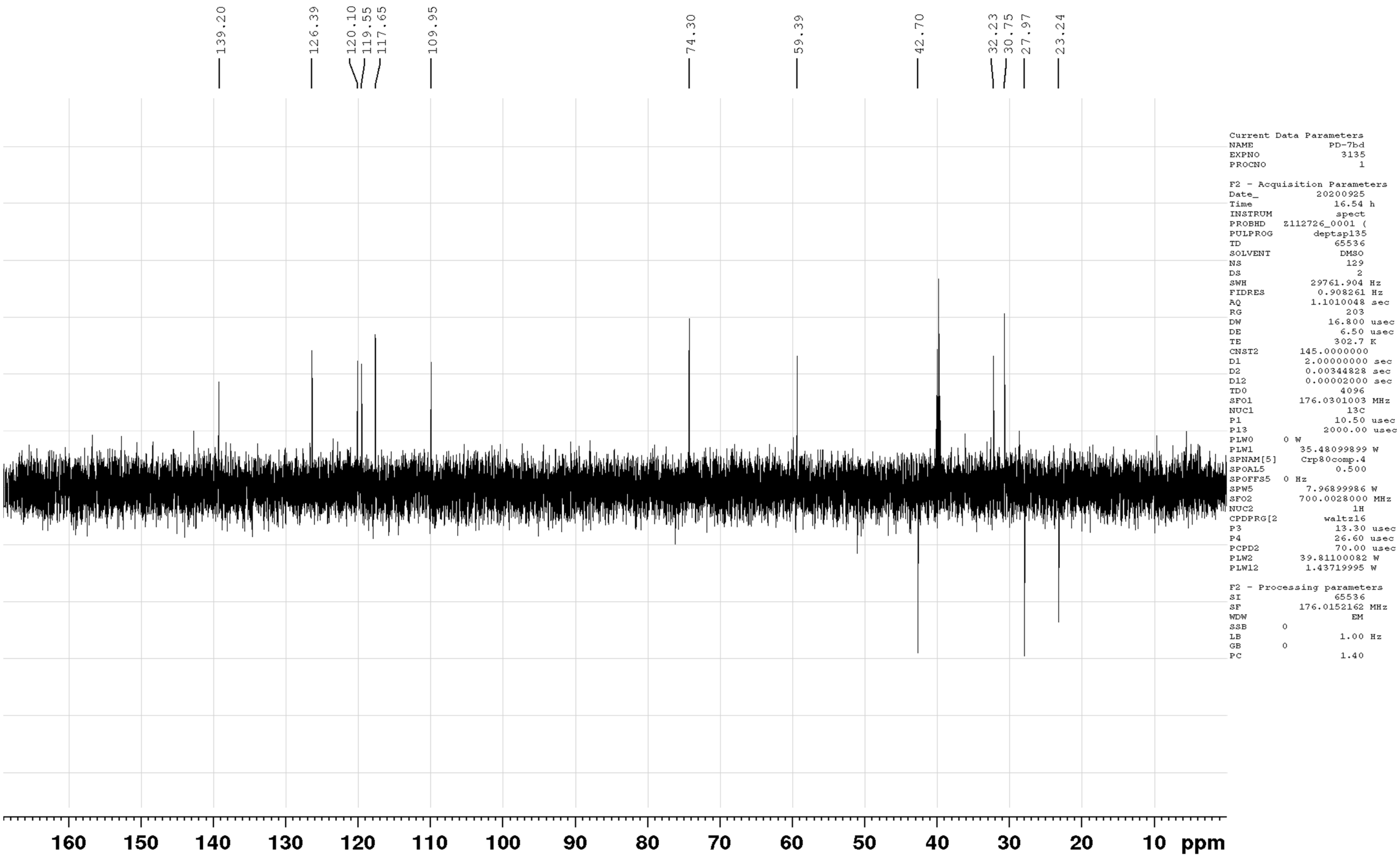

Figure S52. DEPT-135 NMR spectrum (176 MHz, DMSO-d6) of **5**

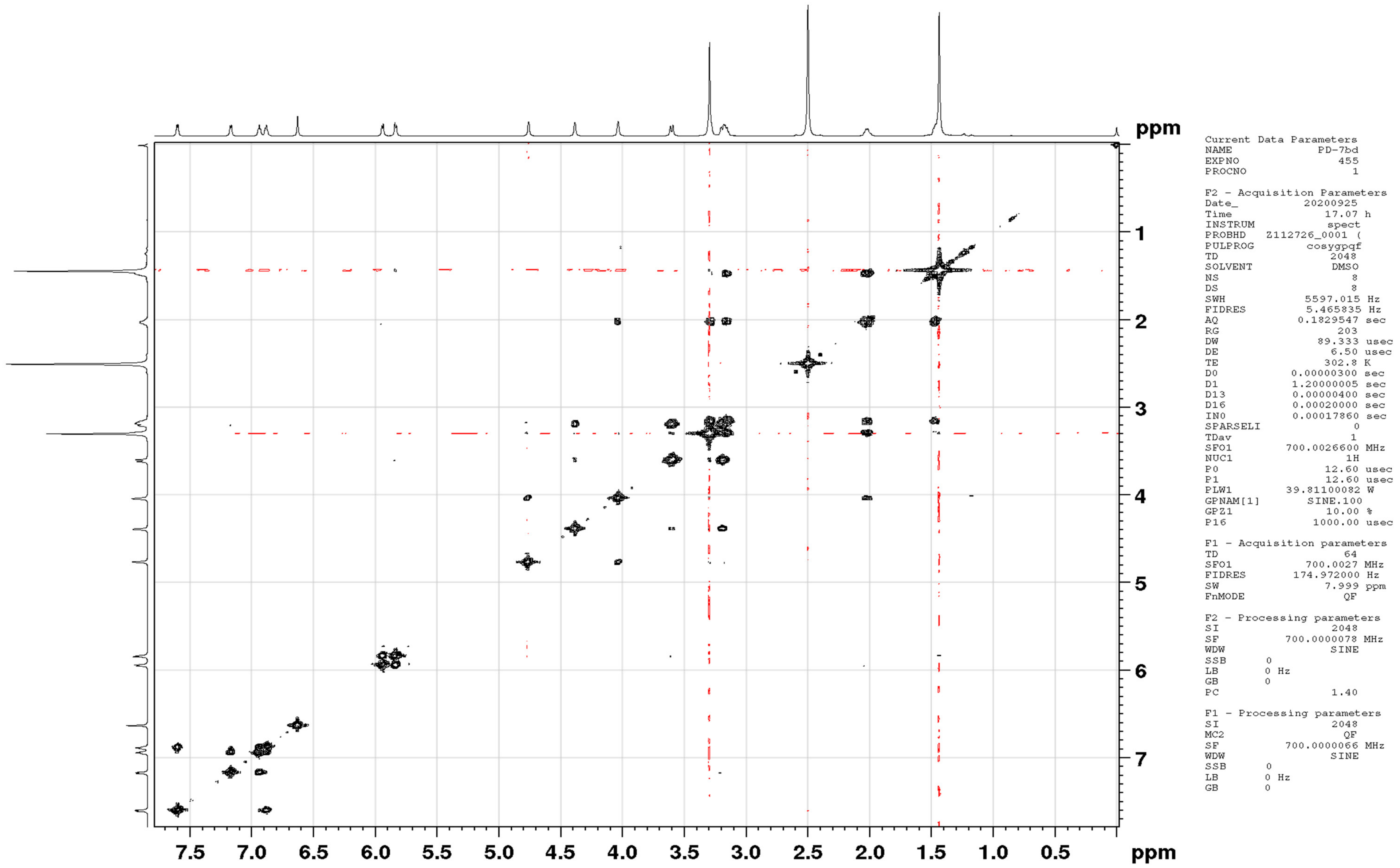

Figure S53. COSY-45 spectrum (700 MHz, DMSO-d6) of **5**

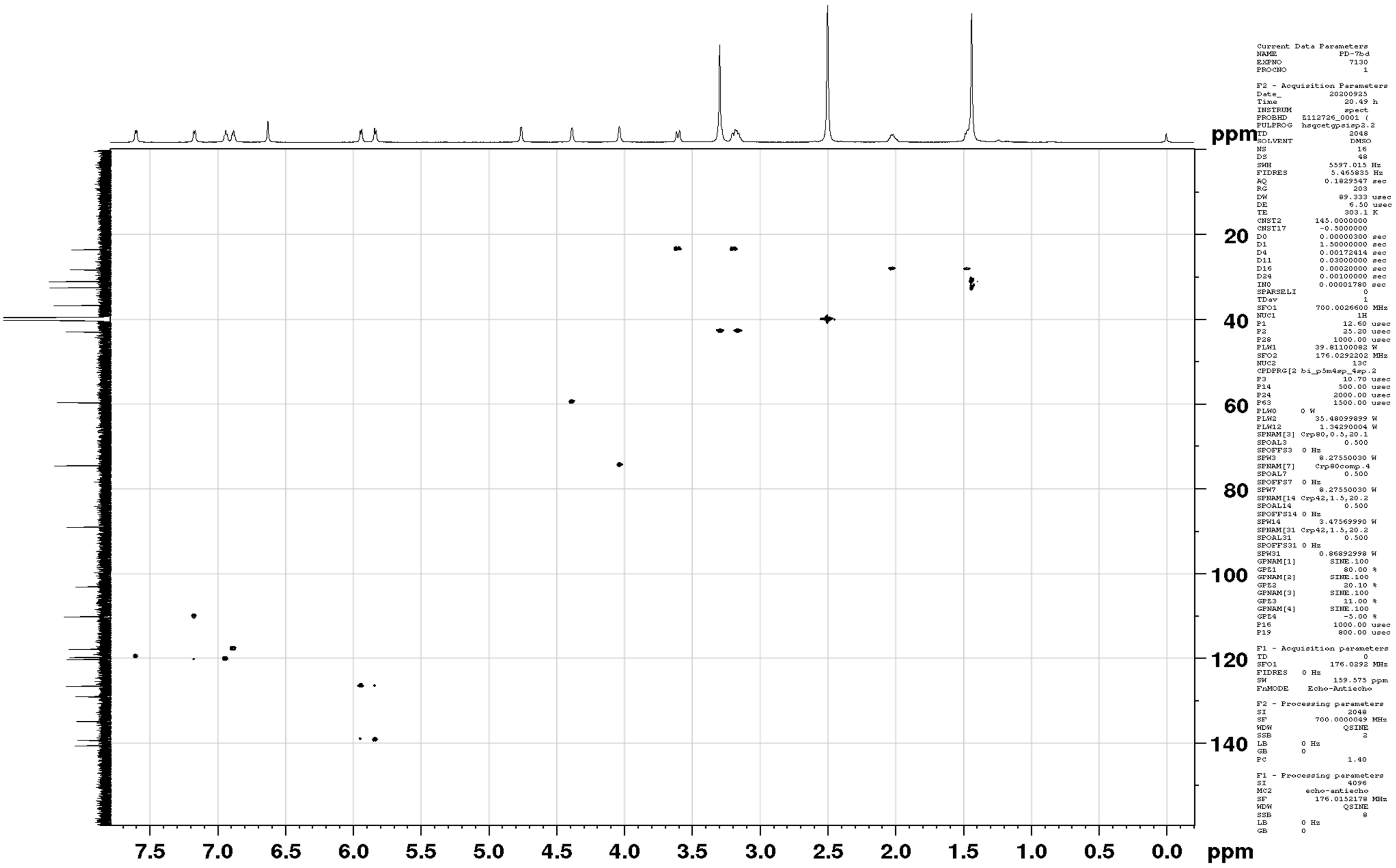

Figure S54. HSQC spectrum (700 MHz, DMSO-d6) of **5**

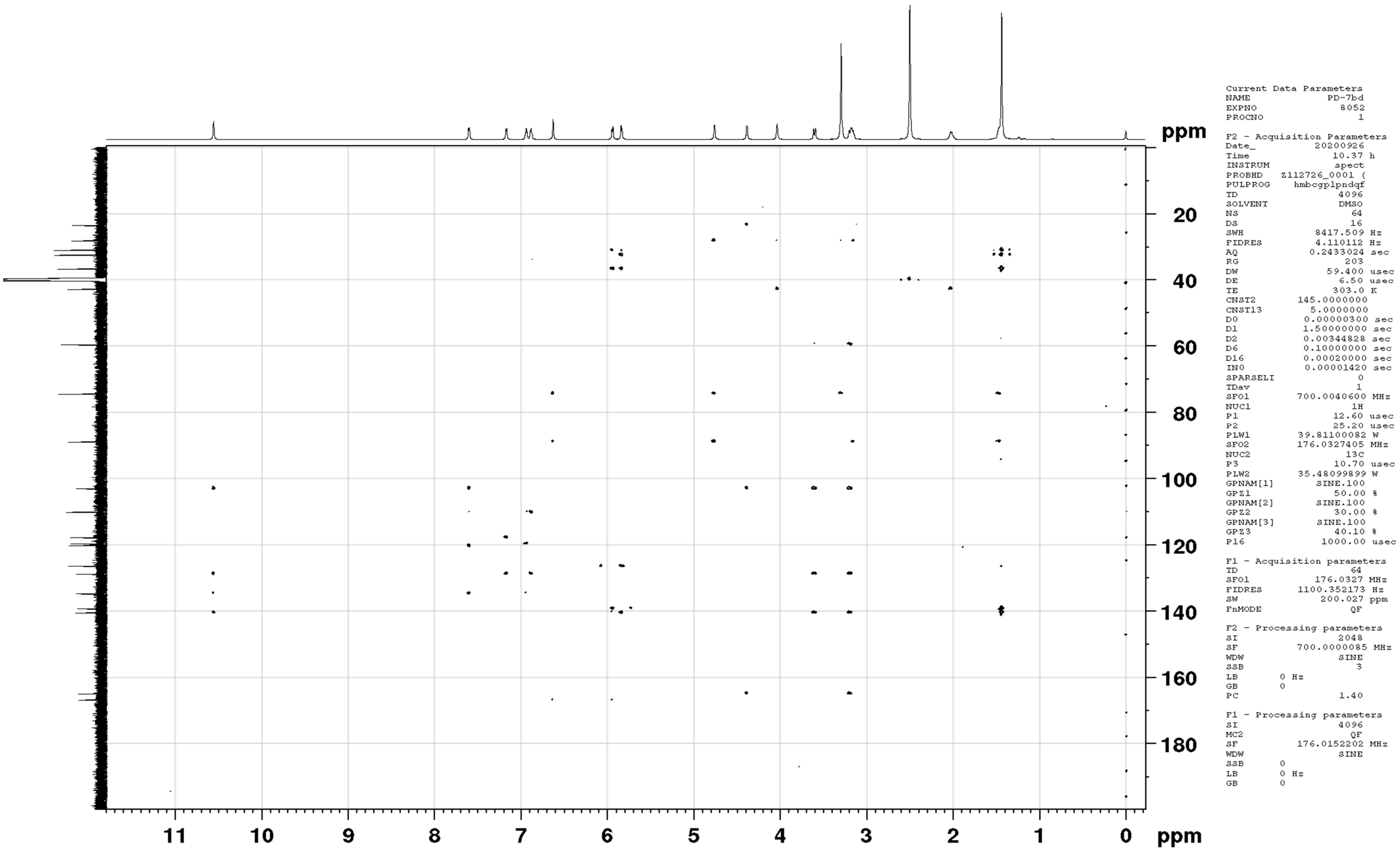

Figure S55. HMBC spectrum (700 MHz, DMSO-d6) of **5**

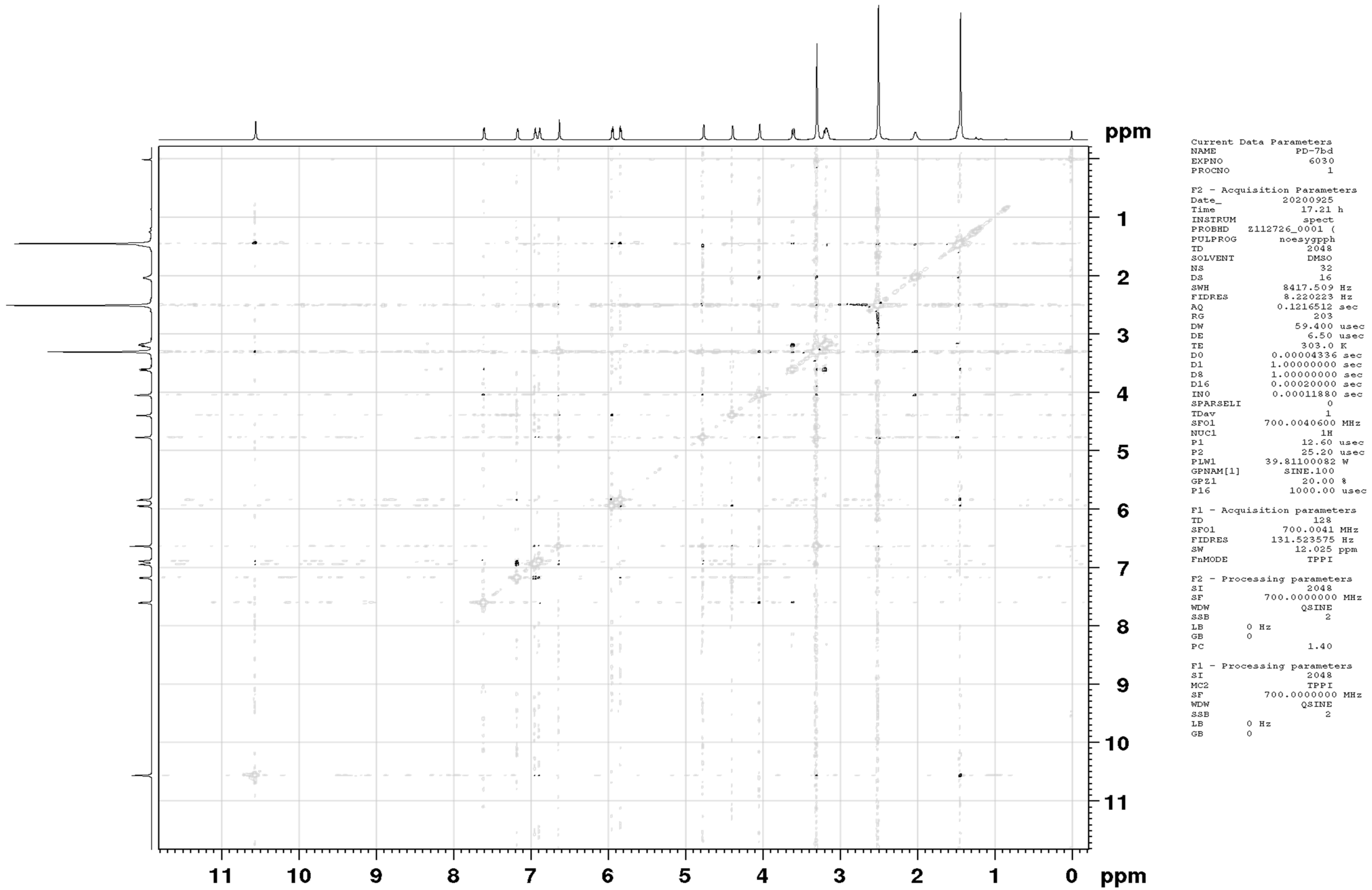

Figure S56. NOESY spectrum (700 MHz, DMSO-d6) of **5**

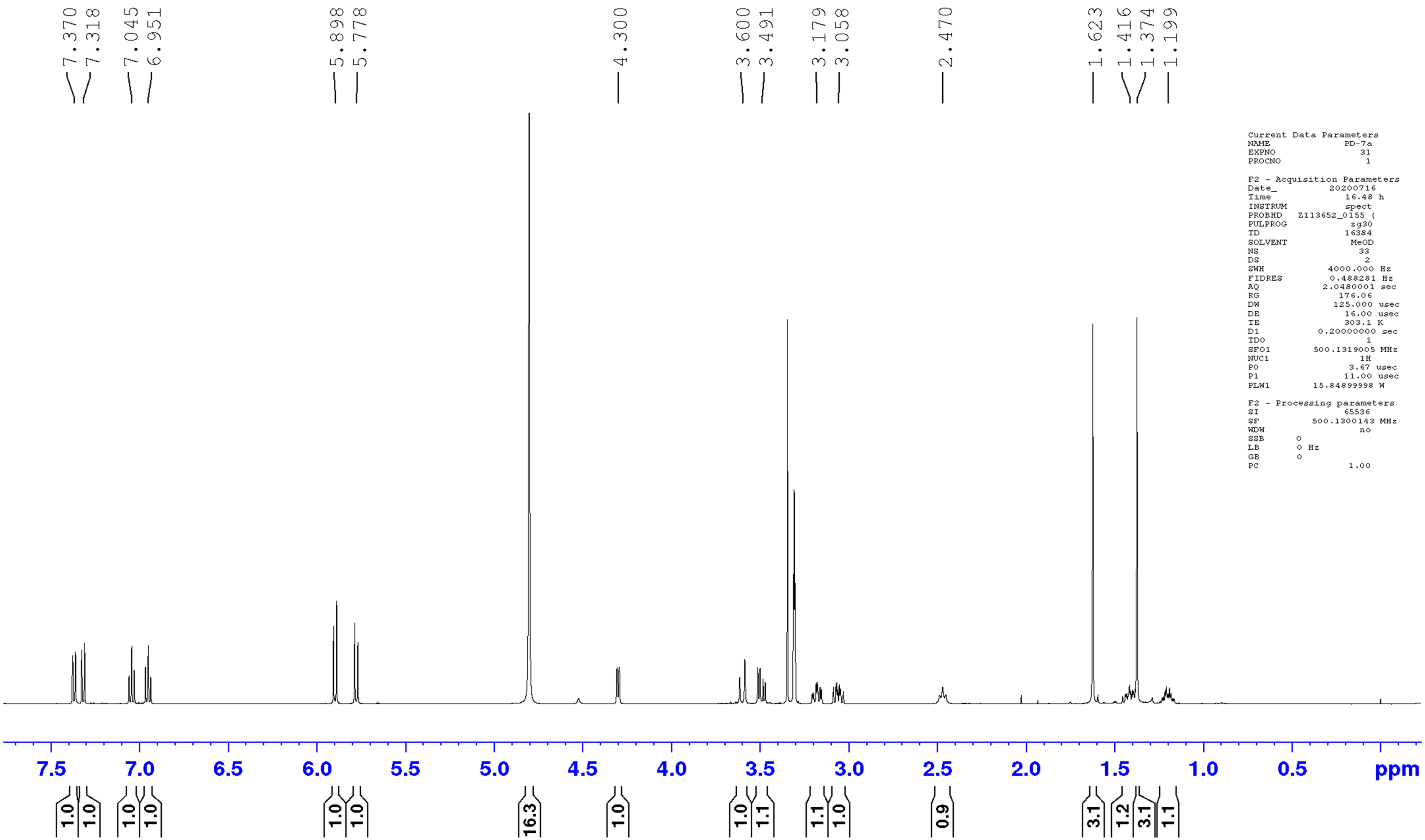

Figure S57. ^{1}H NMR spectrum (500 MHz, CD_3OD) of **6**

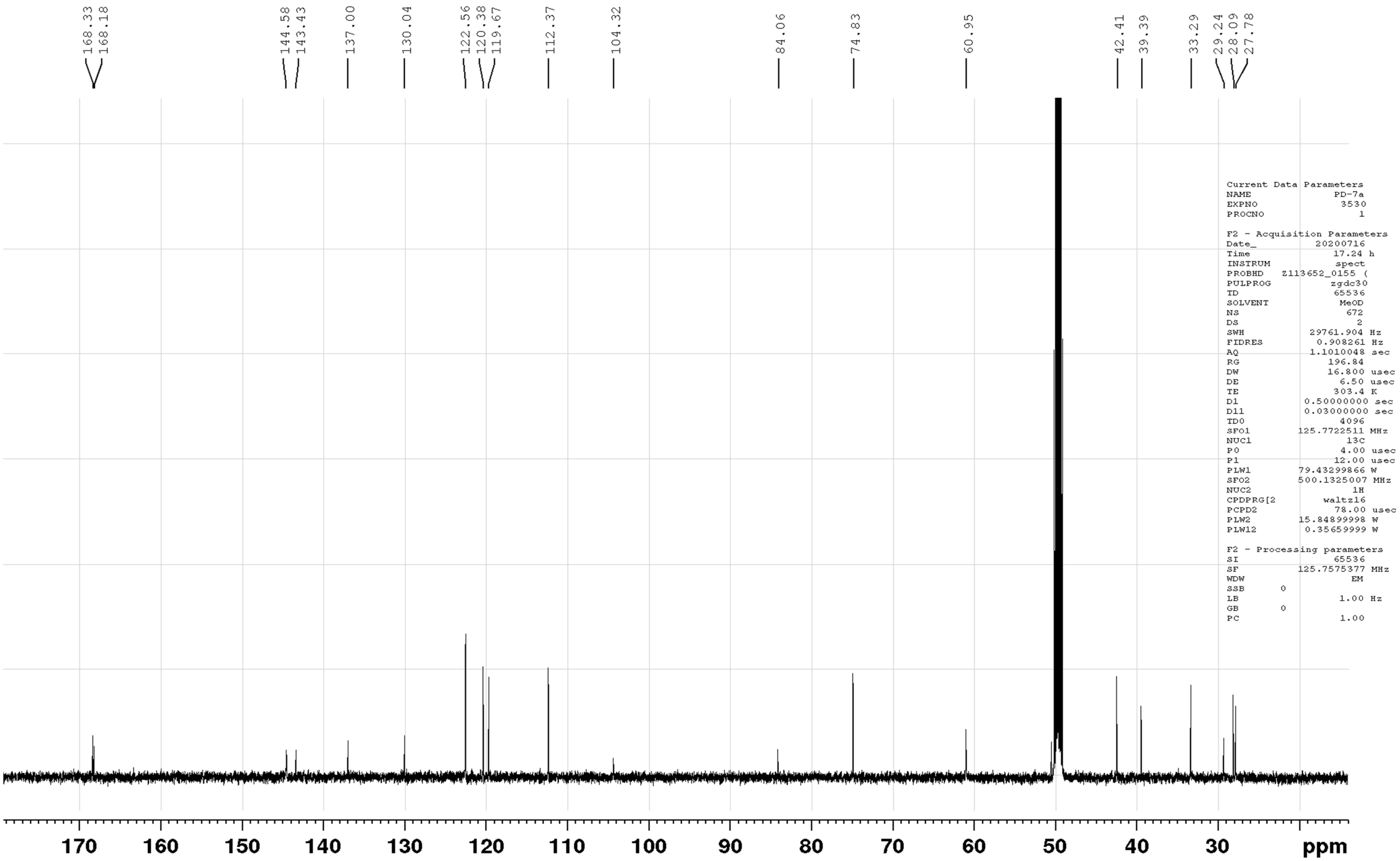

Figure S58. ^{13}C NMR spectrum (125 MHz, CD_3OD) of **6**

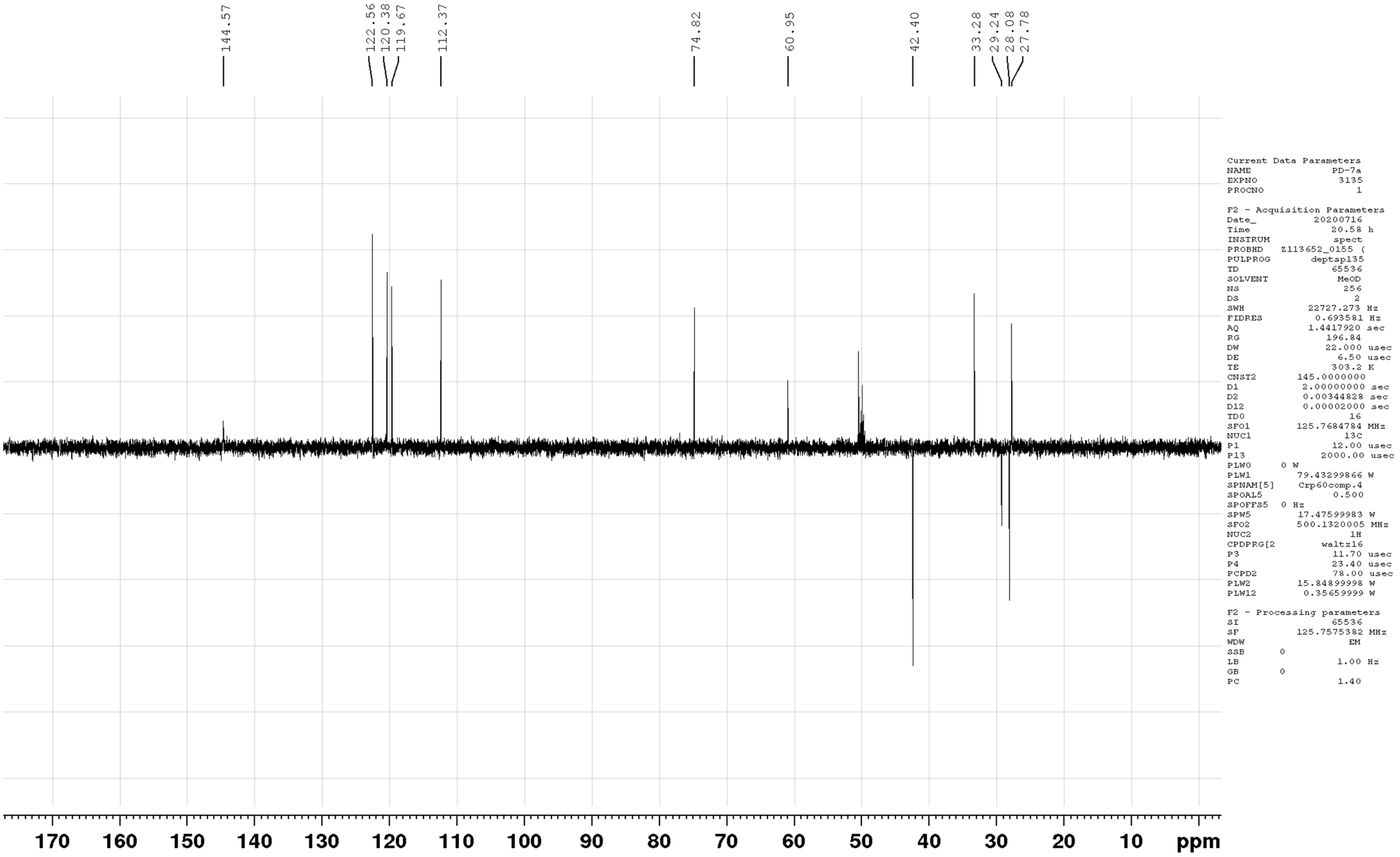

Figure S59. DEPT-135 NMR spectrum (125 MHz, CD_3OD) of **6**

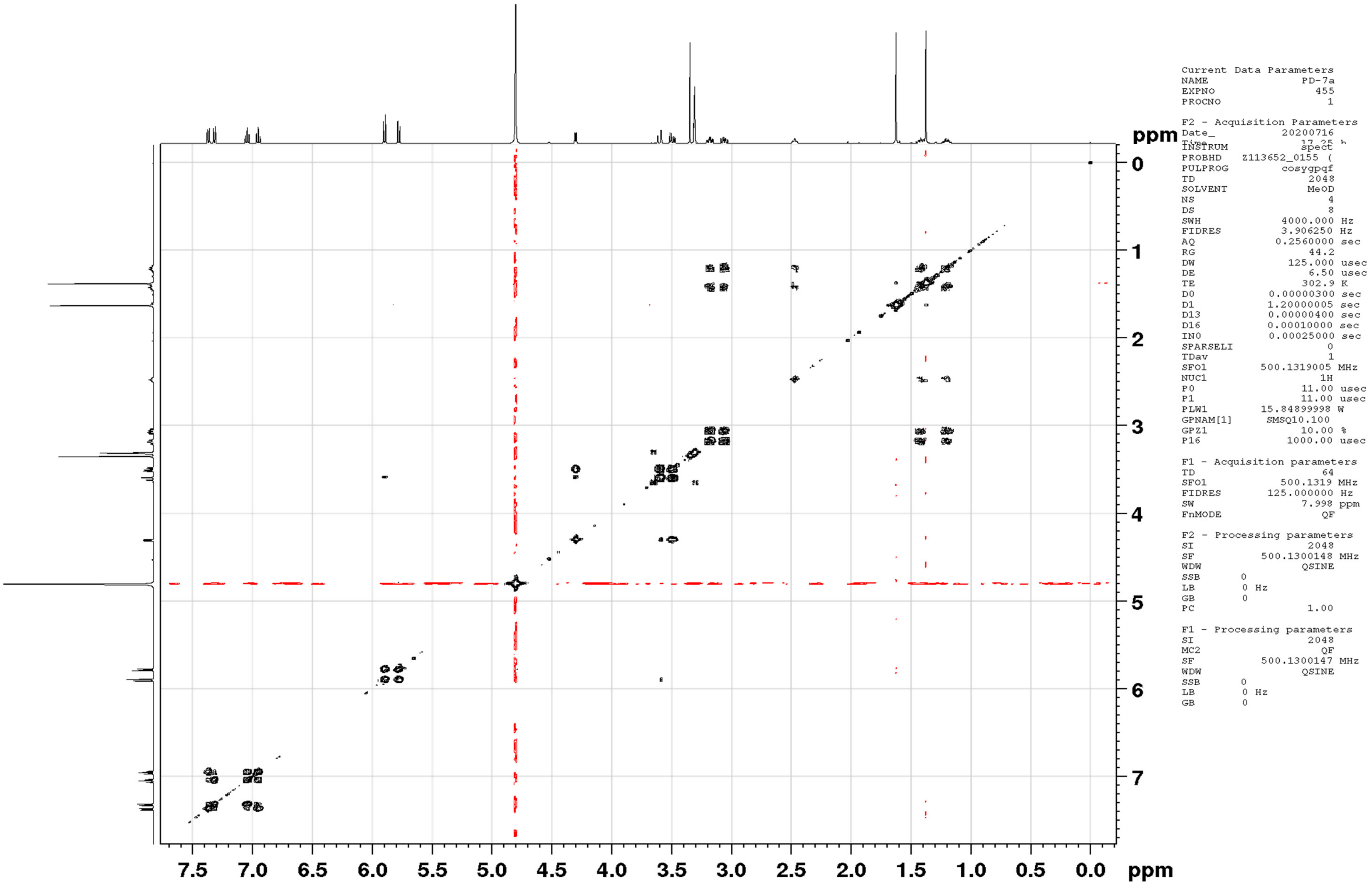

Figure S60. COSY-45 spectrum (500 MHz, CD_3OD) of **6**

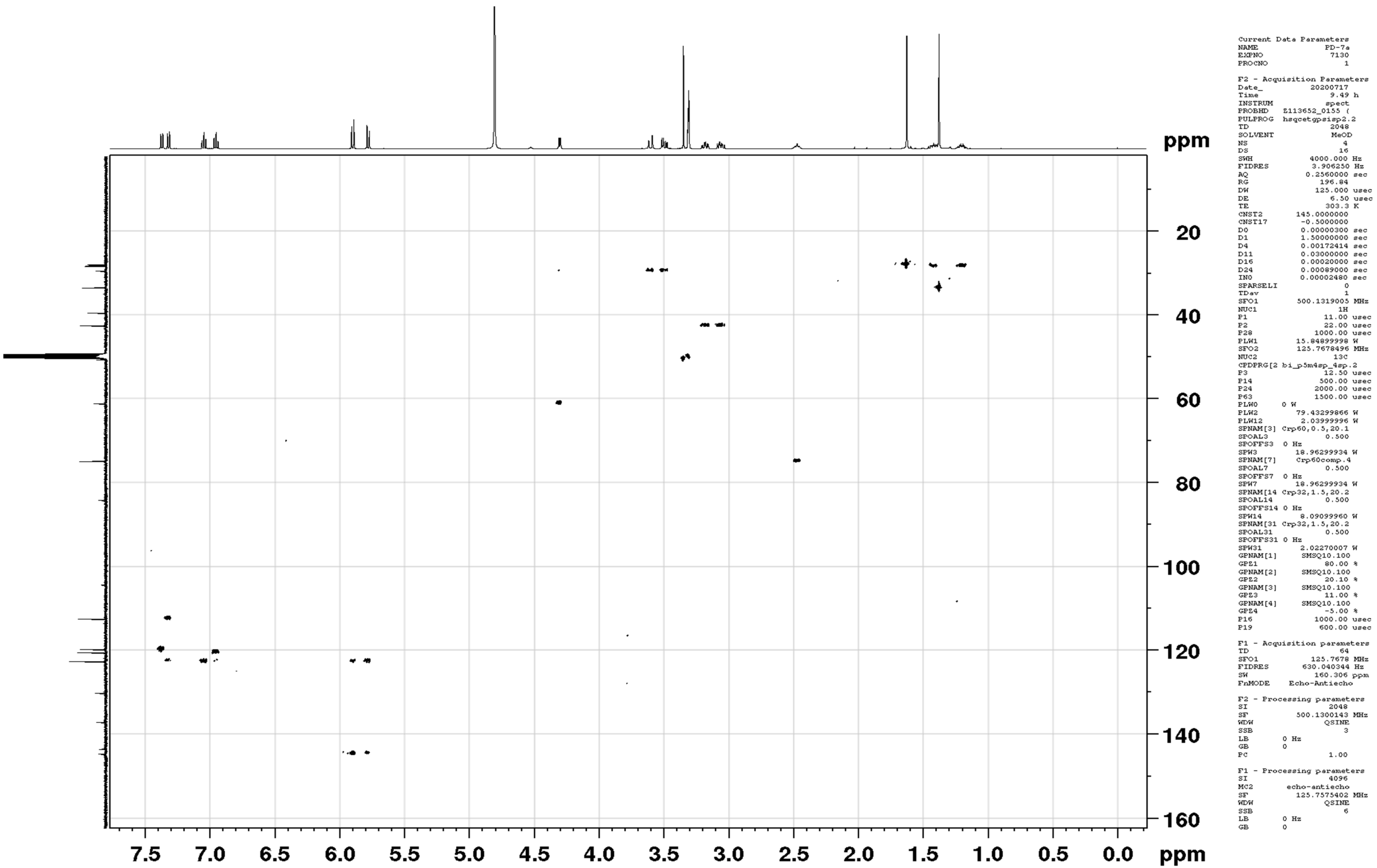

Figure S61. HSQC spectrum (500 MHz, CD_3OD) of **6**

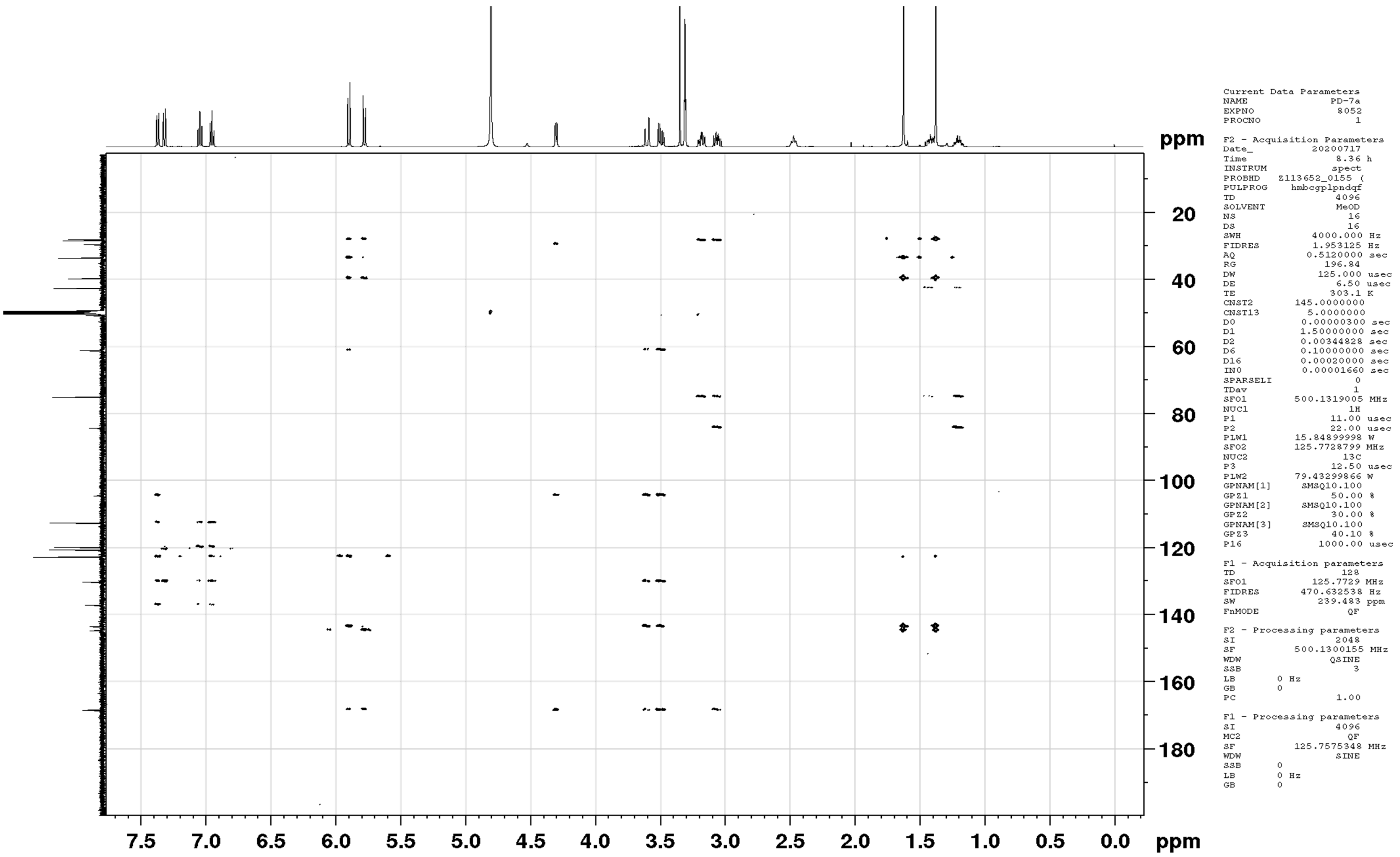

Figure S62. HMBC spectrum (500 MHz, CD_3OD) of **6**

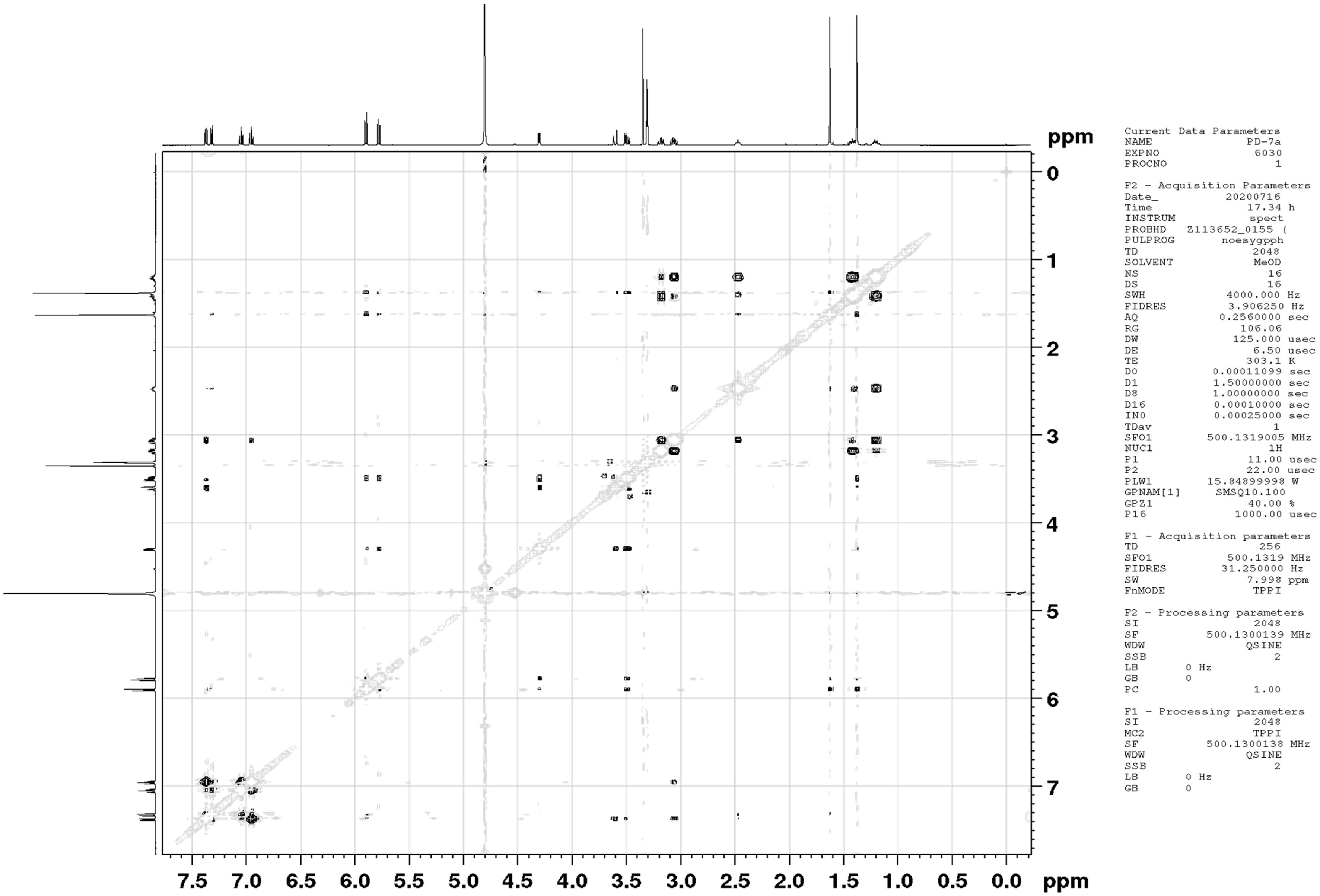

Figure S63. NOESY spectrum (500 MHz, CD_3OD) of **6**

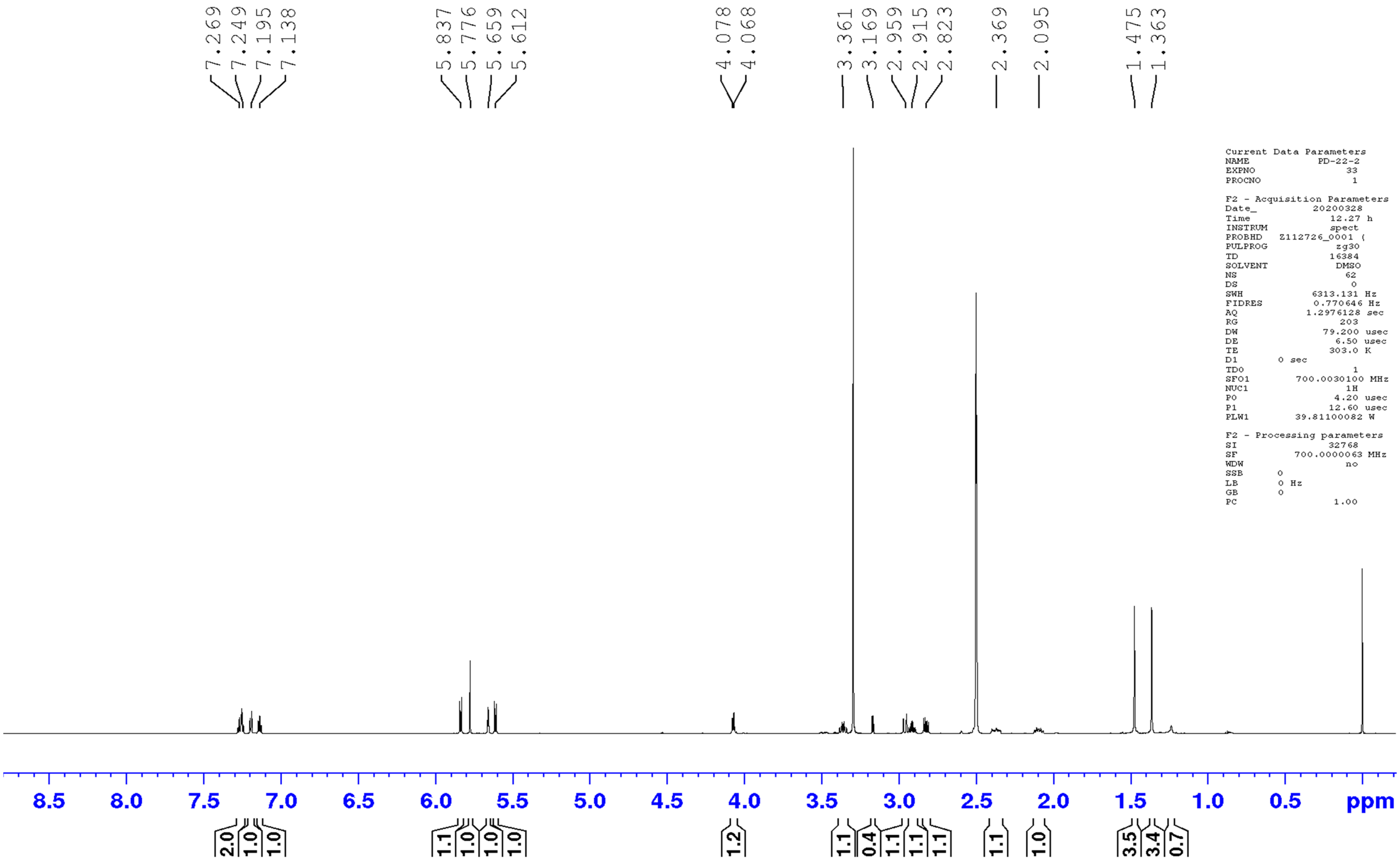

Figure S64. ^{1}H NMR spectrum (700 MHz, DMSO-d6) of **7**

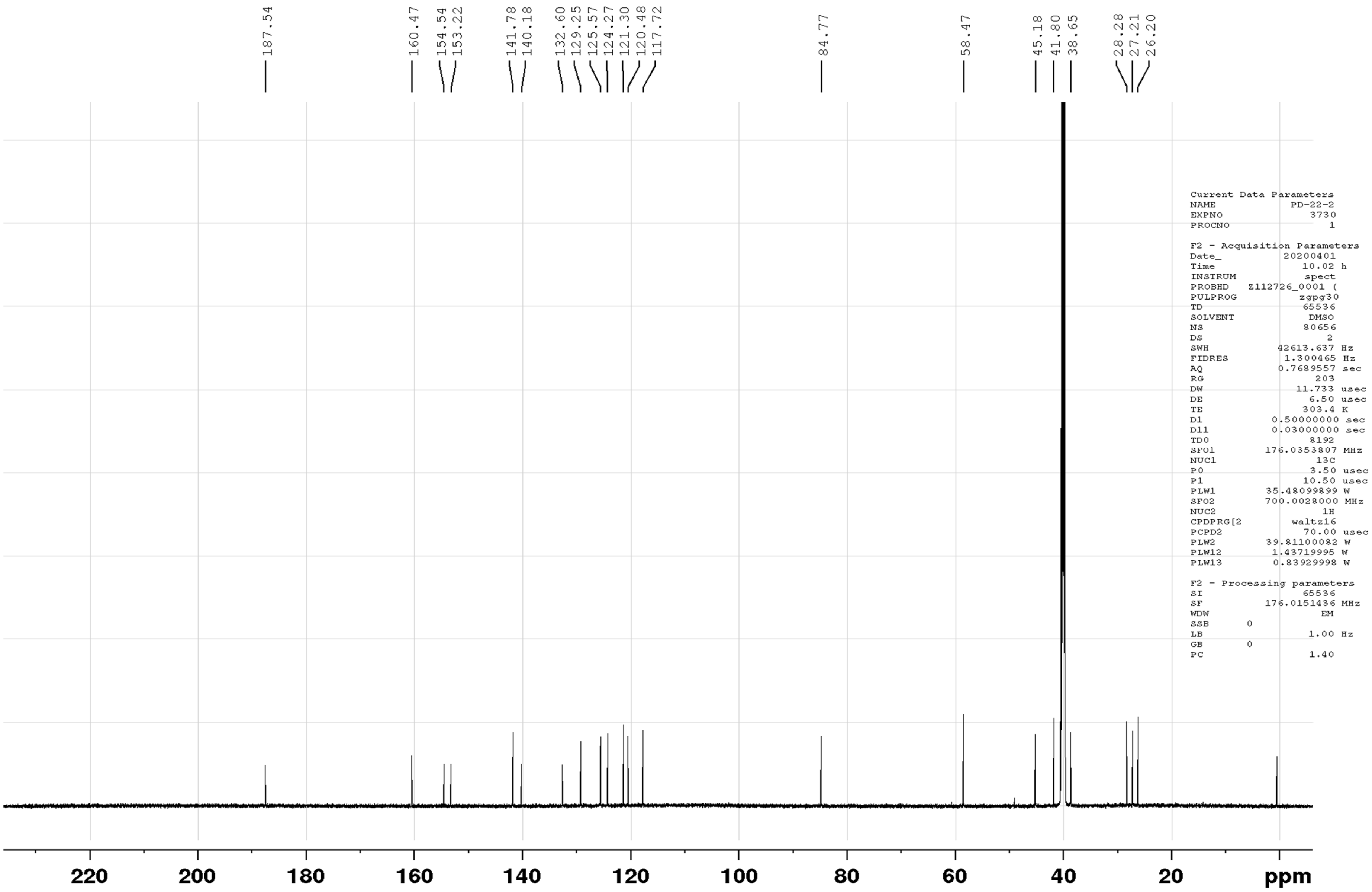

Figure S65. ^{13}C NMR spectrum (176 MHz, DMSO-d_6) of **7**

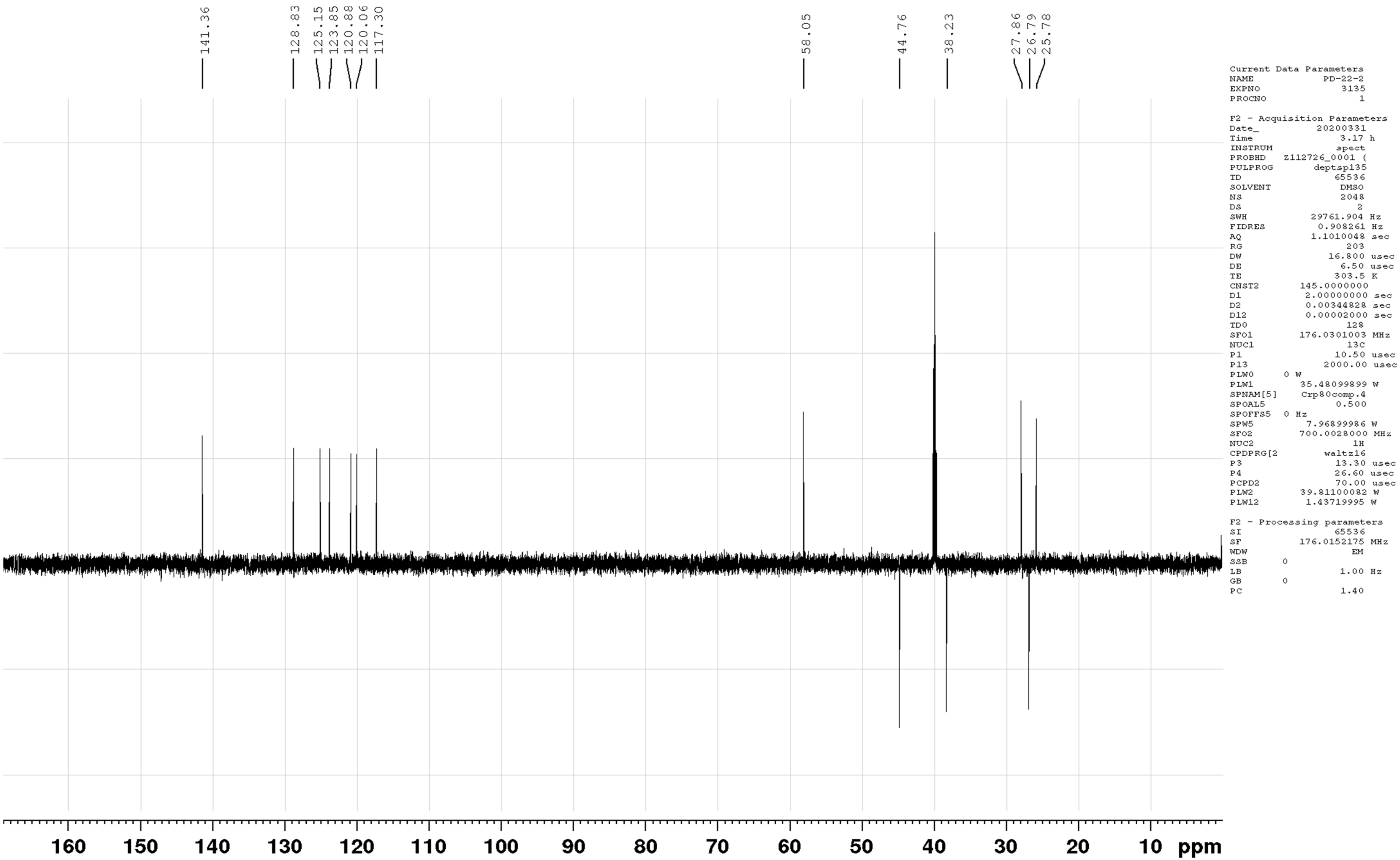

Figure S66. DEPT-135 NMR spectrum (176 MHz, DMSO-d₆) of **7**

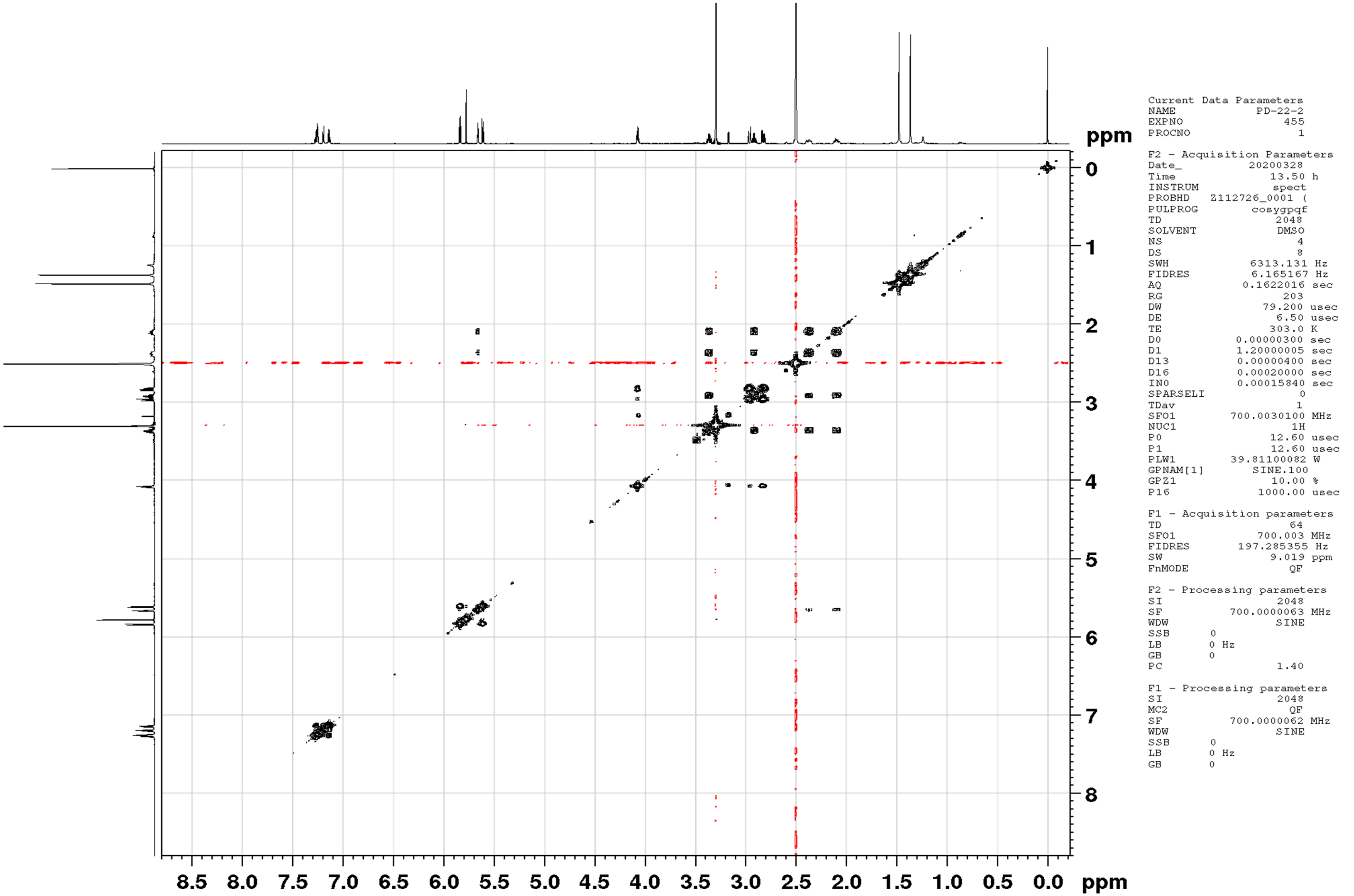

Figure S67. COSY-45 spectrum (700 MHz, DMSO-d6) of **4**

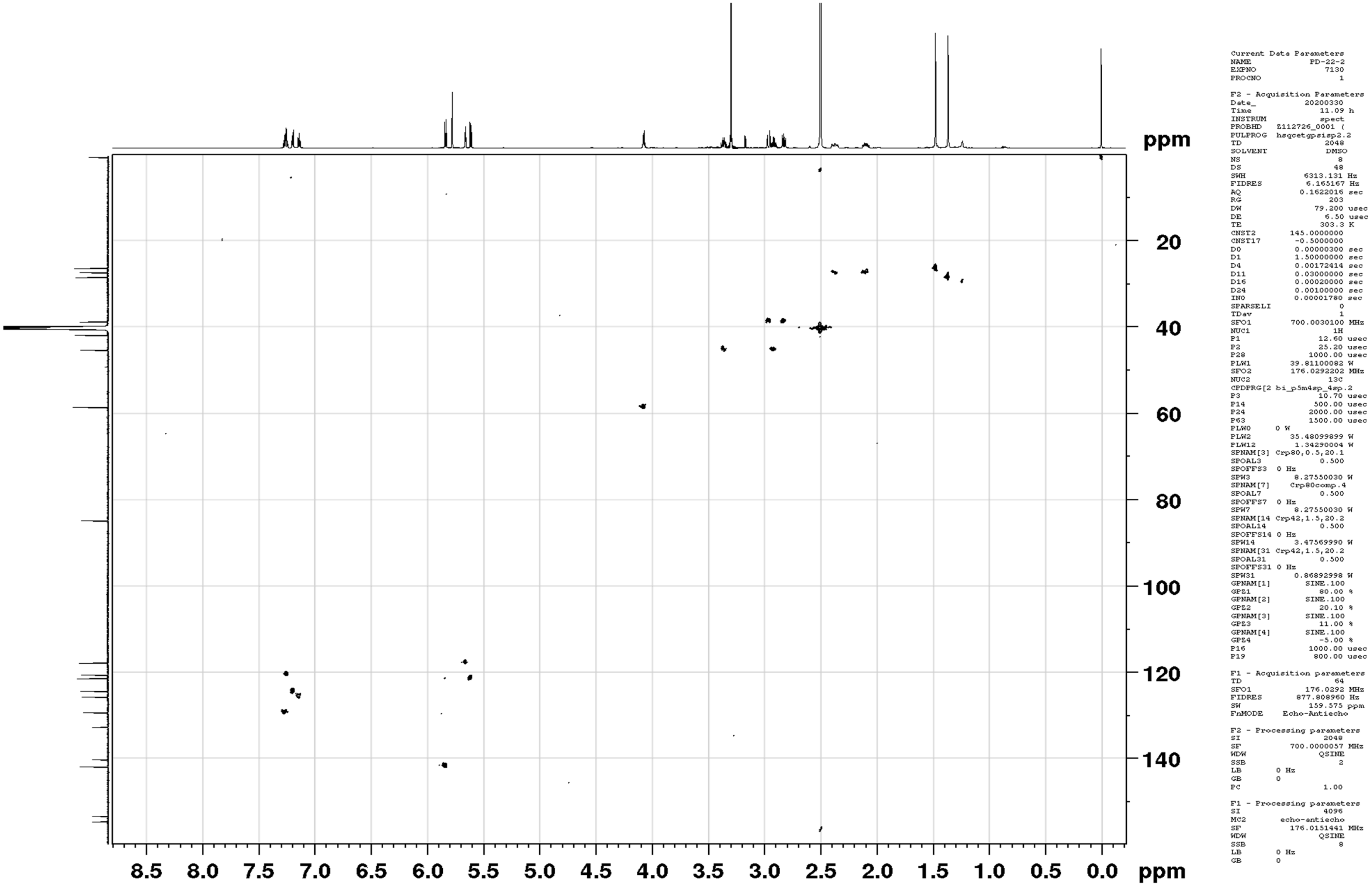

Figure S68. HSQC spectrum (700 MHz, DMSO-d6) of **7**

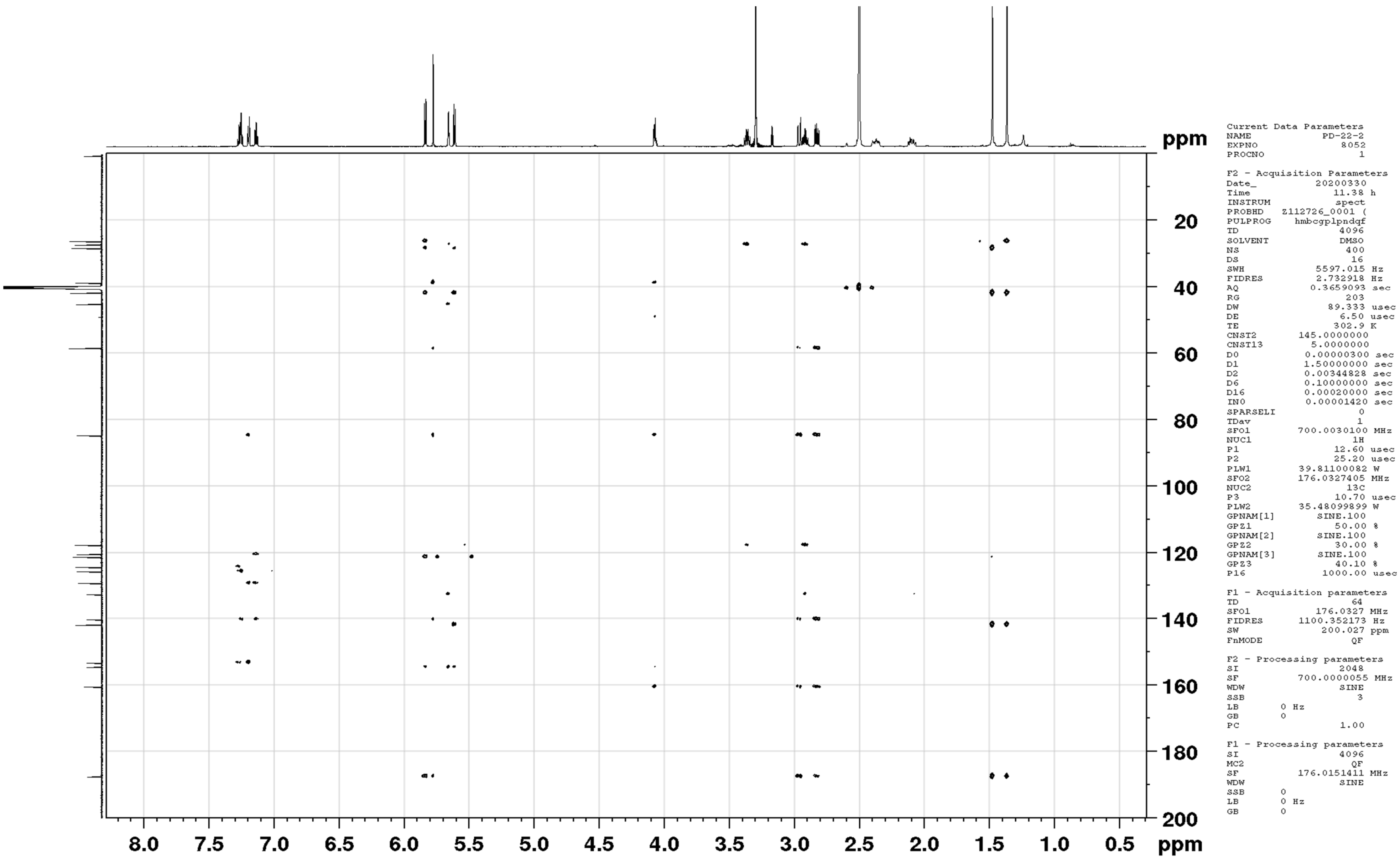

Figure S69. HMBC spectrum (700 MHz, DMSO-d_6) of **7**

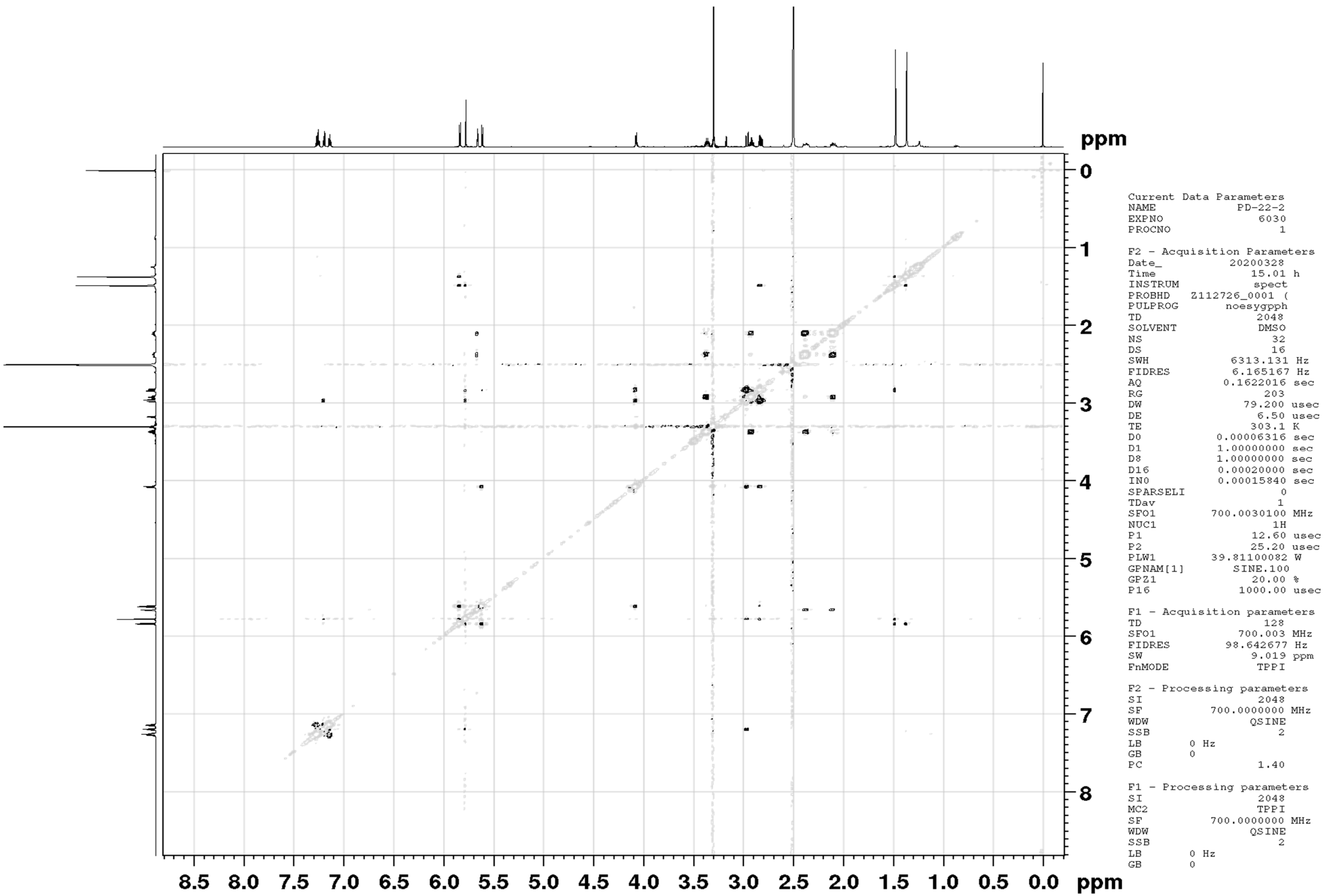

Figure S70. NOESY spectrum (700 MHz, DMSO-d6) of 7

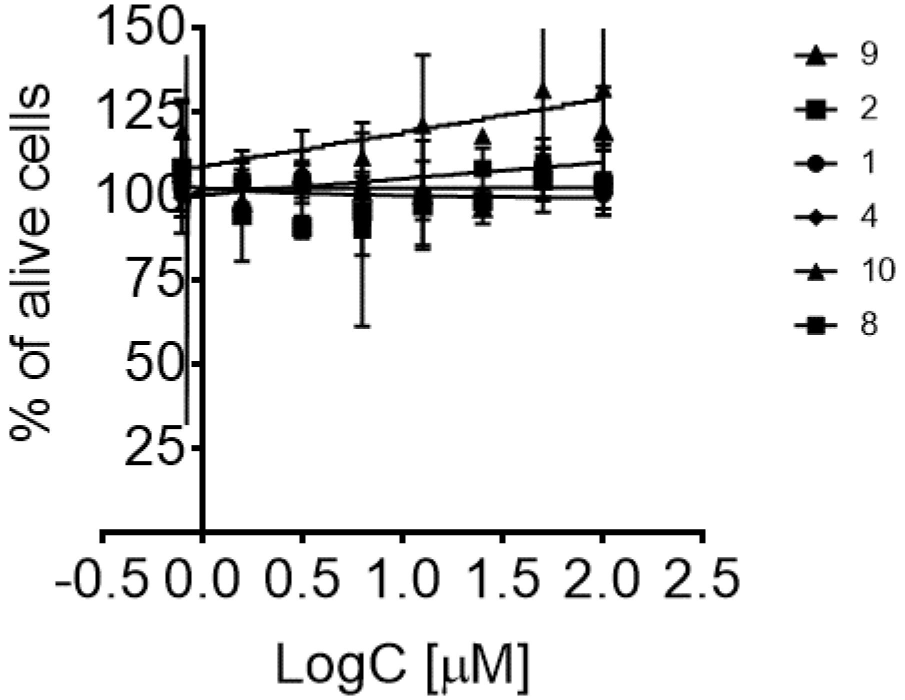

Figure 71. Viability of human prostate PNT2 cells treated with the investigated compounds for 48 h. No significant cytotoxicity was observed for the concentrations of the drugs up to 100 μM.

Table S1 Selected crystal data and refinement parameters for structure **1.**

Formula weight	395.45
Temperature (K)	298(2)
Radiationtype	Mo K_α
Space group	$P2_12_12_1$
Unit cell dimensions (Å)	a = 7.5507(3),<br>b = 12.1354(6),<br>c = 21.985(1)
V ($Å^3$) / Z	2014.5(2), 4
D_{calc}(g/cm^3)	1.304
μ, mm^{-1}	0.091
F(000)	840
Crystal size (mm)	0.45 × 0.41 × 0.28
θ range (°)	1.853 - 32.041
Range of *h*, *k* and *l*	-11<=h<=7, -16<=k<=18, -32<=l<=32
Reflections measured/ unique / with $I>2\sigma(I)$	35597/6978/6055<br>R_{int} = 0.0205
GooF	1.030
Final R indices [I>2sigma(I)]	R1 = 0.0406, wR2 = 0.1116
R indices (all data)	R1 = 0.0484, wR2 = 0.1179
$\Delta\rho_{min}$, $\Delta\rho_{max}$ (e/$Å^3$)	-0.207, 0.208

Table S2 Selected bond lengths (d, Å) in the structures **1**.

O(1)—C(4)	1.224(2)
O(2)—C(9)	1.231(2)
O(3)—C(6)	1.415(2)
O(4)—C(5)	1.402(2)
O(4)—C(22)	1.417(2)
N(1)—C(4)	1.344(2)
N(1)—C(3)	1.437(2)
N(1)—C(10)	1.464(2)
N(2)—C(9)	1.335(2)
N(2)—C(5)	1.456(2)
N(2)—C(8)	1.476(2)
N(3)—C(18)	1.373(2)
N(3)—C(19)	1.379(2)
C(1)—C(2)	1.504(3)
C(1)—C(19)	1.515(2)
C(1)—C(20)	1.534(3)
C(1)—C(21)	1.555(3)
C(2)—C(3)	1.323(3)
C(4)—C(5)	1.525(2)
C(5)—C(6)	1.531(2)
C(6)—C(7)	1.529(3)
C(7)—C(8)	1.535(3)
C(9)—C(10)	1.518(2)
C(10)—C(11)	1.539(2)
C(11)—C(12)	1.491(2)
C(12)—C(19)	1.373(2)
C(12)—C(13)	1.429(3)
C(13)—C(14)	1.405(3)
C(13)—C(18)	1.406(3)
C(14)—C(15)	1.382(5)
C(15)—C(16)	1.387(6)
C(16)—C(17)	1.369(5)
C(17)—C(18)	1.391(3)

Table S3 Hydrogen bonds for structure **1**.

D—H...A	d(D—H)	d(H...A)	d(D...A)	<(DHA)
N(3)—H(3A)···O(1)^{i}	0.94(3)	2.01(3)	2.909(2)	159(2)
O(3)—H(3)···O(2)^{ii}	0.87(3)	1.97(3)	2.825(2)	170(3)

Symmetry transformations used to generate equivalent atoms:
(ii)–x,y–1/2,–z+1/2;(iii) x–1/2,–y+3/2,–z
